# MicroRNA profiling provides insights into post-transcriptional regulation of gene expression in chickpea root apex under salinity and water deficiency

**DOI:** 10.1038/s41598-017-04906-z

**Published:** 2017-07-05

**Authors:** Hitaishi Khandal, Sabiha Parween, Riti Roy, Mukesh Kumar Meena, Debasis Chattopadhyay

**Affiliations:** National Institute of Plant Genome Research, Aruna Asaf Ali Marg, New Delhi, 110067 India

## Abstract

Activity of root apical meristem (RAM) at the root apex is critical for stress-mediated modulation of root-architecture. Chickpea, like other legumes, possesses a basic open root meristem. Deep sequencing was used to perform microRNA expression profiling in root apex of chickpea (*Cicer arietinum* L.) in order to investigate post-transcriptional regulation of gene expression in this tissue in response to salinity and water deficit. Five small RNA libraries prepared from chickpea root apices at different stages of stress treatments were sequenced to obtain 284 unique miRNA sequences including 60 novel miRNAs belonging to total 255 families. Two hundred and fiftynine miRNAs were differentially expressed in stress. Six hundred and nine mRNA targets involved in diverse cellular processes were predicted for 244 miRNAs. Stress-responsive expression patterns of selected miRNAs, inverse expression patterns of their target genes and the target-cleavage sites were validated. Three candidate miRNA-target gene relationships were validated in transient expression system in chickpea. The miRNA expression profiling under salinity and water deficiency in a legume root apex and the reported function of their target genes suggested important roles of miRNA-mediated post-transcriptional regulation of gene expression involved in re-patterning of root hair cells, lateral root formation and high-affinity K^+^-uptake under these stresses.

## Introduction

Decrease in ground water level and increase in salinity limit plant growth and eventually yield in all agricultural ecosystem. Variation in adaptability of a plant in changing soil conditions largely depends on distribution of its root system, commonly referred to as root system architecture (RSA), which is primarily determined by cell division and elongation in primary and lateral roots^1–3^. Cell division in root occurs at a defined region in the root tip, called root apical meristem (RAM), activity of which regulates developmental plasticity of root^4, 5^. The plant hormone auxin is involved in growth of nearly all the plant organs including root. A balance between auxin and another phytohormone cytokinin determines the rate of cell division in RAM^3, 6^. Root is the first plant organ to sense decrease in water and increase in salt concentration in soil^7^ and root growth is highly inhibited by salt mainly due to reduced activity of RAM^8–10^. Phytohormone abscisic acid (ABA) plays a major role in response to water deficit and salinity stresses. Interplay of hormone signaling regulate gene expression and ultimately, determines plant’s response to stress. MicroRNAs (miRNAs), a class of small RNAs (mostly of 20–24 nucleotide), repress gene expression before or after transcription and functions in non-cell-autonomous regulation of plant development by moving between cells within a short distance^11–13^.

Regulation of root growth by miRNAs has been intensely studied. MicroRNA 393 (miR393) and miR160 target *TIR1* (*TRANSPORT INHIBITOR RESPONSE 1*), an auxin receptor, and *AUXIN RESPONSIVE FACTOR 10* (*ARF10*) and *ARF16*, respectively^14^. Overexpression of miR160 caused uncontrolled cell division, loss of gravity-sensing at the root-tip and regulate primary root development^15^. miR164 targeted a transcription factor *NAC1* to downregulate auxin signaling for lateral root initiation^16^. miR167 targeted *ARF6* and *ARF8*, which are positive regulators of adventitious root development^17^. Comparative miRNA expression profiling between the root tip and elongation zone, and between the root-forming callus and non-root forming callus of *Medicago truncatula* (*Medicago*) identified 107 miRNAs, belonging to 44 families, expressing in these tissues and predicted conservation of some of the miRNA/target relationship shown in other species^18^. MiR396 overexpression in *Medicago* root cause cell-cycle gene repression and limited root growth^19^. miRNA expression profiling in normal soybean root and comparative miRNA expression profiling between phosphate-starved and phosphate-sufficient soybean roots identified some novel miRNA/target relationships^20^.

An early study showed upregulation of miR393, miR397b and miR402 expression under dehydration and salt stress in *Arabidopsis* ^21^. miR159 overexpression caused reduction in *MYB33* and *MYB101* transcript accumulation resulting in ABA-hyposensitive *Arabidopsis* plant^22^. miR169a targets nuclear factor *YA5* to reduce its expression and causes enhanced leaf water loss resulting in drought sensitivity^23^. Global analysis of miRNA expression demonstrated important role of miRNA in modulating root growth under abiotic stresses^24–26^. Expression of miR398a/b and miR408 are strongly upregulated in *Medicago* root under drought stress^27^. In rice, miR169g expression was enhanced in root under drought stress^28^. miRNA165/166 targets transcripts of leucine-zipper family proteins to regulate root growth in *Arabidopsis* ^29^. Overexpression of miR160 affected root growth and nodule number in *Medicago* ^30^. Recently, an analysis of small RNA population from root tips of soybean seedlings under normal and salt stress conditions identified total 71 miRNA candidates belonging to 59 known and novel miRNA families. Among them 66 were found to be salt responsive and most of them contained auxin-responsive *cis*-elements in their promoter regions, which indicates that these miRNAs may be regulated by auxin. It was suggested that auxin signaling plays a crucial role in regulation of the miRNAs and root development in soybean^31^. All these information highlight a major role of miRNA in shaping root RSA by regulating gene expression in root and, thereby, RAM activity.

Chickpea (*Cicer arietinum*), an economically important crop ranks fourth among legumes in terms of production (FAOSTAT, 2009). Drought and salinity stresses are among the major reasons that attenuate its production. Chickpea is highly sensitive to salinity, even a tolerant cultivar dies within 75 days when exposed to 40 mM sodium chloride^32^. Recently, draft genome assemblies of chickpeas have been made available^33, 34^. Small RNA libraries prepared from seven different tissues of chickpea identified 440 conserved and 178 novel miRNAs based on sequence similarity^35^. A miRNA profiling under wilt and salt stress in chickpea through high throughput sequencing identified 122 conserved miRNAs belonging to 25 different families and 59 novel miRNAs^36^. Sequencing of small RNA population from leaves and inflorescence identified 157 miRNA loci for 96 conserved known miRNA homologs that belong to 38 miRNA families in chickpea^37^. Unlike a closed RAM structure in *Arabidopsis*, RAM of leguminous plants exhibits a basic-open structure^38^. We report here the dynamics of miRNA expression in chickpea root apex at different stages of water deficit and salinity stresses by high throughput sequencing of small RNA libraries to understand regulation of gene expression by miRNA in this tissue under these stresses.

## Results

### Histogram of chickpea root apex

**Figure 1:**
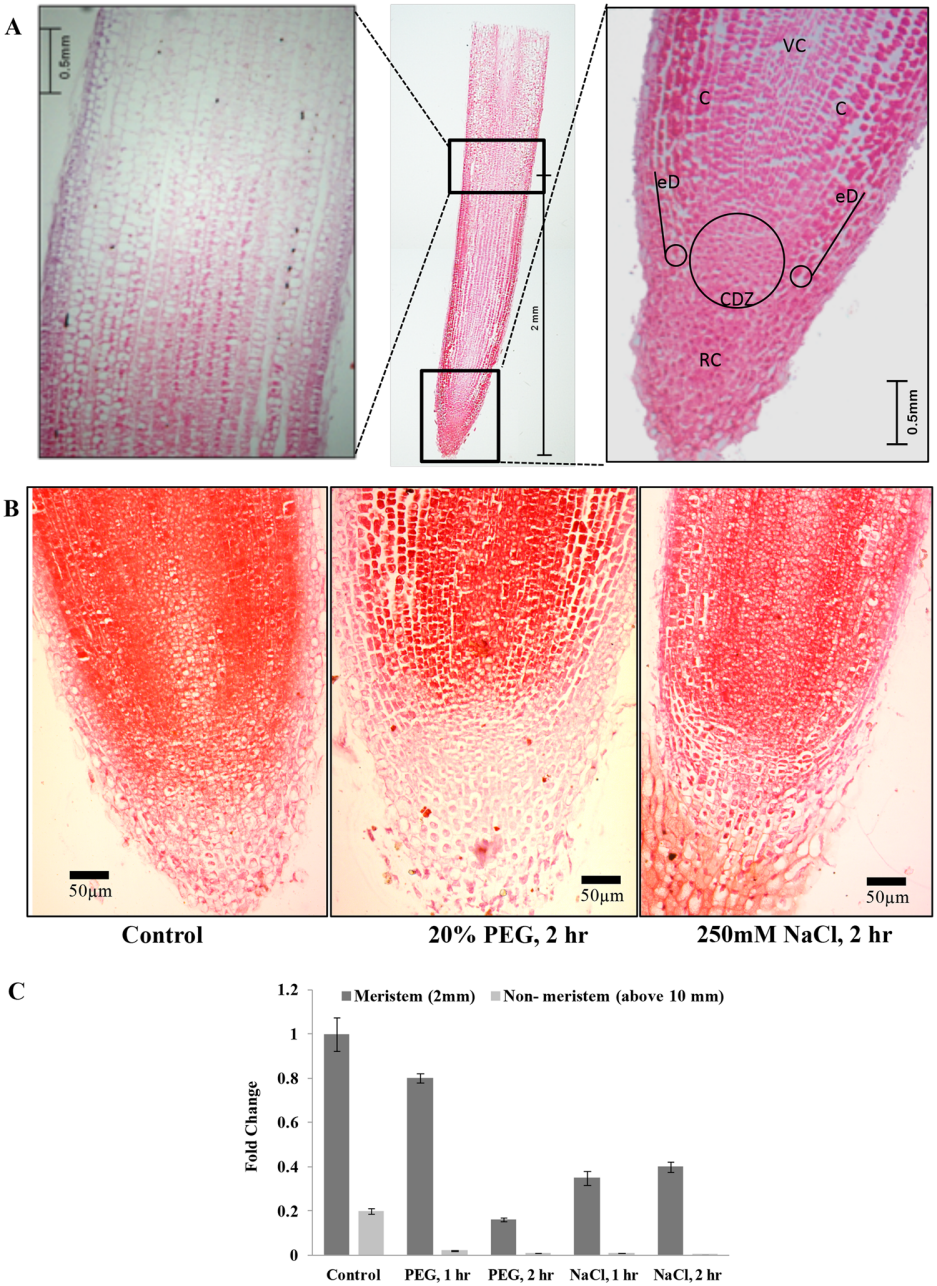
Chickpea root apex histogram. (**A**) A median longitudinal section (6 µm) of 3 mm root tip of 6dpg chickpea stained with saffrainin showing structure of root cell division zone. (**VC**- Vascular cylinder, **C**- Cortex, **eD**- Epidermis, **CDZ**- Cell division zone, **RC**- Root cap). The root apical meristem (RAM) and the upper cell division zone were shown as magnified images. (**B**) A comparison of chickpea RAM treated with water, 20% solution of polyethylene glycol (PEG) and 250 mM sodium chloride for 2 hours. (**C**) Expression level of *CYCLINA2*, estimated by qRT-PCR, in 2 mm region of root apex and in the same size of tissue above 10 mm of root apex. Chickpea elongation factor 1 α (*CaEF1α*) was used as internal control. Standard deviations of three replicates were shown.

A median longitudinal section of 3 mm in length and 6 μm in thickness and stained with saffrainin (Fig. 1A) displayed a basic-open organization of root tip of 6 dpg chickpea seedlings. Unlike *Arabidopsis* root tip, which shows a closed organization, initial cells in chickpea root tip are not clearly distinguishable within the tightly packed mass of dividing cells. The magnified view of root tip showed a large number of densely packed cells. Instead of root cap cells organized in layers as in *Arabidopsis*, chickpea root cap cells were more in numbers and did not peel-off in layers. 3 mm section showed undifferentiated densely packed cells surrounded by border cells and root cap. Endodermis is composed of evenly-sized cells. Root hairs were absent in this section and initiation of vascular bundle appeared above 2.5 mm from the root tip as an average of more than thirty samples viewed. Similar observation has been reported for *Medicago truncatula* roots^39^. The root tips treated with 20% PEG or 250 mM sodium chloride for two hours showed deep-stained blocks of relatively separated cells indicating loss of water from the cells (Fig. 1B). Therefore, we sampled 2 mm of root tips of 6 dpg chickpea seedlings as representatives of cell division zone in chickpea root apex to study miRNA expression under water-deficit and salinity stresses for one and two-hour time periods. The status of cell division at root tip before and after stresses were assessed by comparing expression level of a cell division marker gene *CYCLINA2* by qRT-PCR (Fig. 1C). Expression level of *CYCLINA2* was expectedly lower in the differentiation zone of root tissue than in the cell division zone at normal growth condition. Upon exposure to PEG and salt for two hours, the expression level was substantially reduced in the root apex suggesting reduction in rate of cell division. Representative semi-quantitative RT-PCR has been shown in Supplementary Figure S1.

### Expression of genome-annotated chickpea miRNAs in root apex

**Figure 2:**
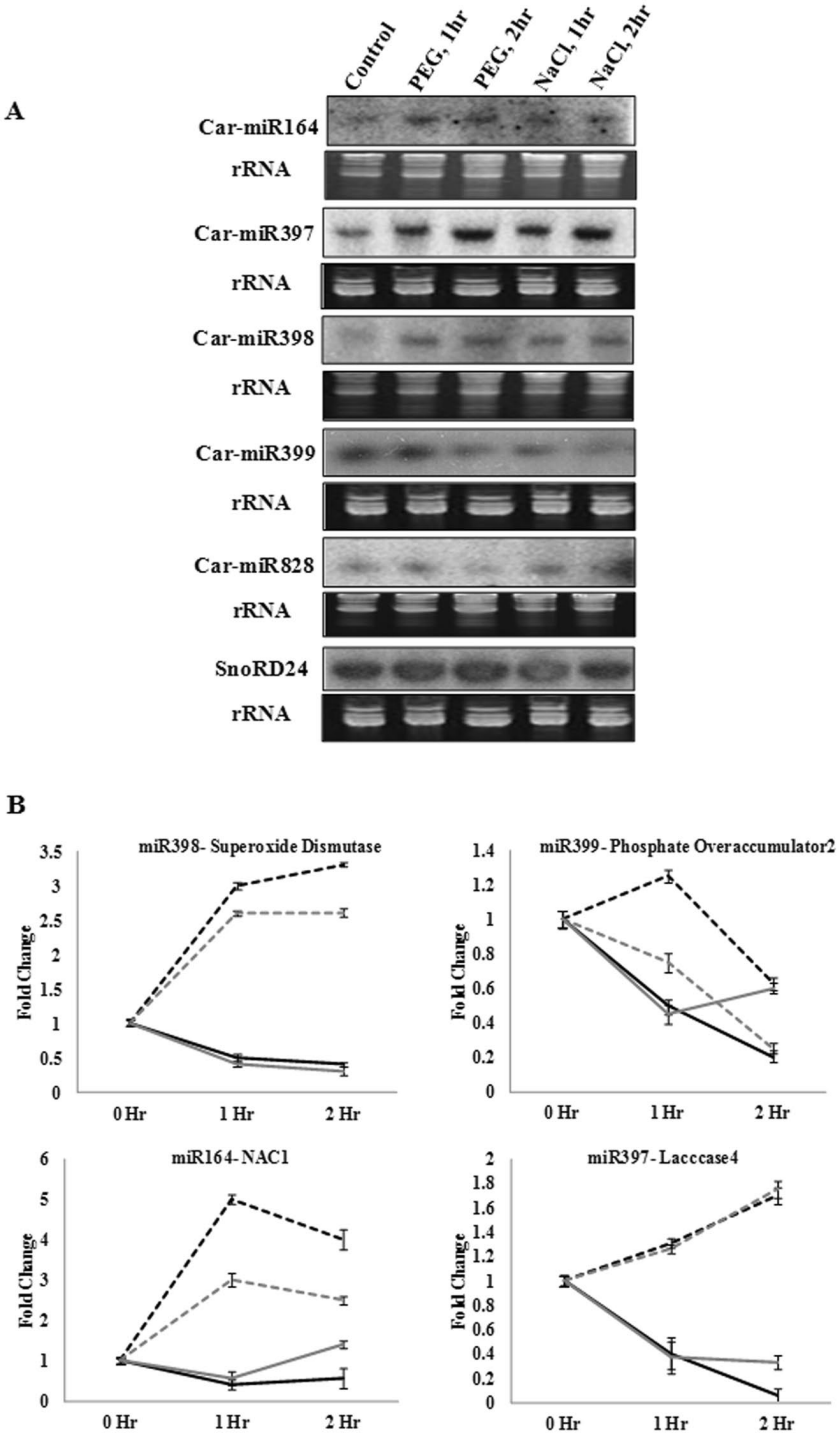
Expression analysis of genome-annotated conserved miRNAs in chickpea root apex under PEG and salt treatment. (**A**) Northern blot was performed to analyse expression levels of miRNAs at 2 mm region of chickpea root apex after 1 hr and 2 hrs of PEG and salt treatments. 15 µg of enriched small RNA from control and treated samples was loaded on denaturing (7 M urea) polyacrylamide (15%) gel. Radiolabeled antisense probes were used for hybridization. Ethidium bromide-stained small RNAs were shown for equal loading. SnoRD24 was used as control. (**B**) Expression pattern of the miRNAs shown in Fig. 2A and their predicted target genes in response to the same treatments as assessed by qRT-PCR. Standard deviations of three replicates were shown. *CaEF1α* was used as internal control for normalization. Grey and black lines represent salt and water deficit stress treatments, respectively. While dotted and solid lines represent miRNA and target gene, respectively.

Of total 34 miRNA families annotated in two draft genome assemblies of chickpea^33, 34^, sixteen families were in common. Expression of one representative member from each of these miRNA families in root and shoot tissue were validated by northern blotting (Supplementary Figure S2). Expression of these miRNAs in the root apex with and without stresses was assessed (Fig. 2A and not shown). Expression levels of miR397, miR398 and miR164 were upregulated in the PEG- and salt-treated samples, while that of miR399 was downregulated in both the time periods. Expression of miR828 was increased at early time points, however, was subsequently decreased by the prolonged exposures to PEG and salinity. Other miRNAs showed negligible differences in expression by the stress treatments. Similar expression patterns of miRNA164, miRNA399 and miR172 in chickpea and soybean^31^ under salt stress suggests common function of these miRNAs under stress in root apex. Target genes were predicted for these conserved miRNAs (Supplementary Table S1). The predicted targets of miR397, miR398, miR164 and miR399 were *LACCASE4* (XP_004490949.1), *COPPER SUPEROXIDE DISMUTASE* (Cu-*SOD*) (NP_001296637.1), *NAC1* (XP_004488843.1) and *PHO2/UBC24* (XP_004485781.1), respectively. Expression profiles of these target genes in the same stress conditions showed negative correlations with the expression profiles of the corresponding miRNAs suggesting possible miRNA-target relationship (Fig. 2B). Laccase is a group of polyphenoloxidoreductases involved in lignin metabolism^40^ and was reported as a target of miR397^21^. Copper SUPEROXIDE DISMUTASE (Cu-SOD), a scavenging enzyme of reactive oxygen species (ROS)^41^, was shown to be regulated by miR398^42^. miR164 targeted *NAC1* in *Arabidopsis* and *Zea mays* and suppressed lateral root formation^16, 43^. PHO2 is a ubiquitin conjugating enzyme and a negative regulator of phosphate accumulation in low phosphate condition^44^. Expression profiles of these miRNAs and their target genes suggested activation of miRNA-mediated regulation of suppression of lignin metabolism and root development in the root apex during water deficit and high salinity.

### High throughput sequencing of chickpea small RNAs from chickpea root apex under water deficit and salinity stresses

For high throughput miRNA expression profiling during exposure of the root apex to water deficit and high salinity, five small RNA libraries were constructed for two different time periods (1 hr and 2 hrs) of each treatment and the control sample using concatenated enriched smRNAs method to increase throughput^45^. These five chickpea small RNA libraries were sequenced by Roche/ 454 pyrosequencing platform. Due to the concatenation strategy, a total of 1,106,081 fragmented individual reads were obtained from 803,259 high quality sequencing reads after adapter removal (Supplementary Figure S3), demonstrating a 37.5% increase in throughput. A total of 108,242 smRNA (9.8% of total) reads of size range 18–24 nts were obtained from five libraries. The largest fraction of those was of 24 nt in length (25.17%) followed by 20 nt (19.24%), 21 nt (14.07%), 22 nt (8.8%) and 23 nt (8.6%). Remaining 23.04% of all sequences in this size range was of 18–19 nts in length (Supplementary Figure S4A). Thus, almost 75.96% of the small RNAs were within the range of 20–24 nt. This size distribution of the smRNA reads agreed with the previously published reports^46–48^.

Identification of miRNAs was performed by following an analysis pipeline described in the methods (Supplementary Figure S5). Sequences with single read count were discarded. The tRNA/rRNA/snoRNAs sequences were filtered out and rest of sequences were mapped on chickpea draft genome assembly resulting in 395 unique sequences showing perfect match. These were screened for sequence similarity with the reference dataset derived from miRBase v21^49^ following a criterion of maximum 2 nts mismatch with maximum 2 nts gap and end extension resulting in 224 miRBASE-matched unique sequences. The putative precursor sequences of the other 171 smRNAs were checked for secondary structure prediction with the following criteria; the characteristic stem-loop structure, one arm of miRNA having less than six mismatches with the opposite arm, no larger loop or break in the miRNA structure, high negative value for folding free energy. Sixty smRNAs satisfied these criteria and deemed novel (Supplementary Table S2). The numbers of novel miRNAs were designated according to their subsequent position on chromosomes. The miRNA* abundance was low in the libraries. Both miRNA and miRNA* sequences for 102 miRNAs were obtained, of which 31 are for novel miRNAs, suggesting occurrence of novel miRNAs in chickpea (Supplementary Table S2).

154 of total 284 predicted miRNA sequences were of 21 nt in size, while 90 miRNAs were represented by 22 and 24 nt-long reads and 40 miRNA sequences were derived from 19 and 20 nt-long reads (Supplementary Figure S4B). 162 miRNA sequences started with a 5′-uridine as the characteristic feature of DCL1 cleavage and AGO1 association^46, 50–52^. The average GC content of mature miRNAs in chickpea (45.40%) in our study was similar to that previously reported for chickpea (44%)^35^.

Physical positions of these identified miRNAs in the chromosomes were determined using the genomic co-ordinates of draft genome assembly^33^. Out of 284 miRNA sequences, 277 could be mapped on this genome assembly and 235 (68.31%) were present on eight chromosomes. All the miRNAs were evenly distributed in all the eight chromosomes without any clustering according to their positions and sequence (Supplementary Figure S6). Majority (267) of them were located in intergenic regions, while 17 miRNAs were of genic origin, of which three were from 3′UTR, eight were from intron and the remaining six were from CDS (coding sequence) (Supplementary Table S3). 284 miRNA sequences could be mapped on the genome assemblies of soybean (*G. max*), *Medicago* and poplar (*P. trichocarpa*). 241 miRNAs were present in all of them, 267 in soybean, 258 in *Medicago* and 255 were present in poplar with a maximum of 3 mismatches over the total length (Supplementary Figure S7).

### High throughput expression analysis of miRNAs

**Figure 3:**
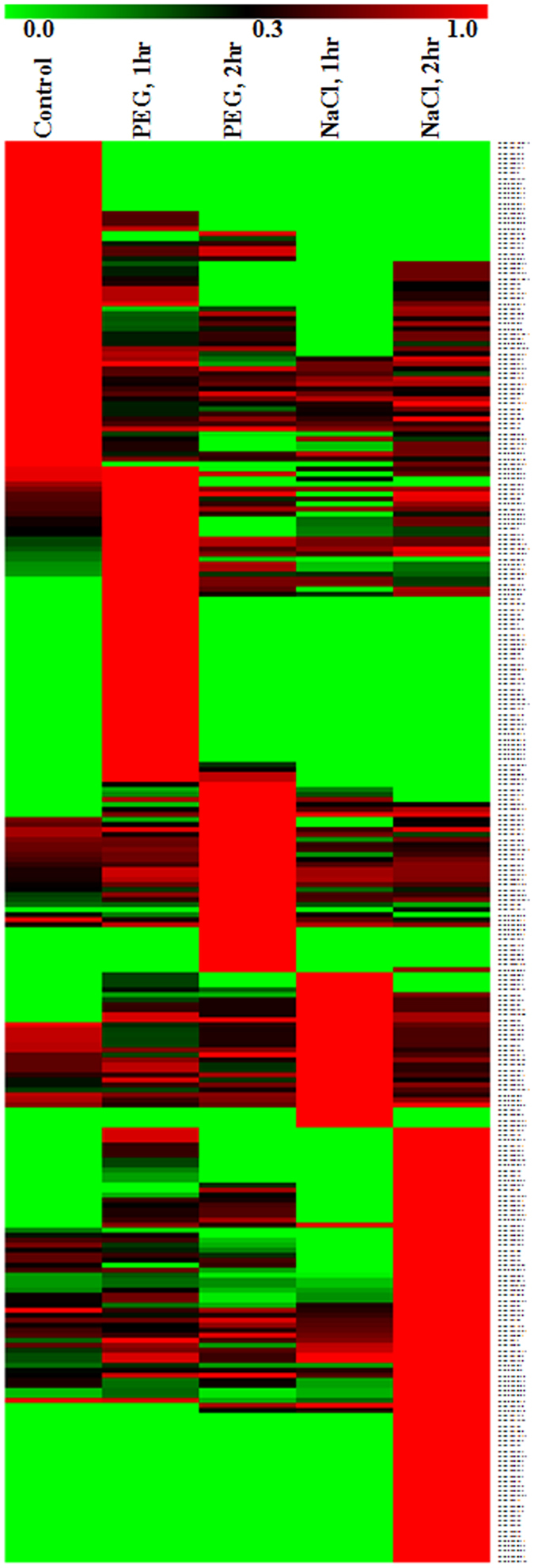
Expression profiling of miRNAs at different stages of treatments with PEG and salt. Heat map representing fold expression of 284 miRNAs based on their normalized reads per million (RPM) values. RPM values of a miRNA were normalized by the highest RPM value of that particular miRNA in any library to have fold expression value of a miRNA at a time point. The miRNAs were clustered according to their normalized expression.

Fold expression of miRNAs were expressed in normalized RPM (reads per million) values. RPM values of a miRNA were normalized by the highest RPM value of that particular miRNA in any library to have fold expression value of a miRNA at a time point. Expression heat map was generated with the fold expression values for all the 284 miRNA sequences (Fig. 3). A minimum of two-fold change in expression was considered as altered expression and accordingly, 63 miRNAs did not show any significant change upon stress treatments. Expressions of 11 miRNAs showed upregulation only in the PEG-treated samples, while 22 miRNAs were downregulated. Forty-six miRNAs were present only in the PEG-treated samples and, therefore, were considered as PEG-upregulated. Similarly, expressions of 12 miRNAs was upregulated in the salt-treated samples, while 13 miRNAs showed downregulation. Thirty-four miRNAs were present only in the salt-treated samples and, therefore, were considered as salt-upregulated. Expression of 56 miRNAs exhibited upregulation in both the stress-treated samples, while 23 miRNAs showed downregulation upon both of these treatments. Four miRNAs namely, Car-miR7635, Car-miR96a, Car-miR7330 and Car-miR7687.1 exhibited upregulation in one treatment and downregulation in the other. Relative expression values of the miRNAs were presented as heat maps with respect to their positions in each chromosome (Supplementary Figure S6). Some genomic regions harbouring miRNAs showing notably altered expression under stress treatments were delineated. Such clusters were present in all the chromosomes (Chm) except in Chm 1. Chm 4 and Chm 7 had two such expression clusters.

### Target gene prediction and inverse correlation of expressions of miRNAs and target genes

Six hundred and nine mRNA targets were predicted for 244 miRNAs using the tool psRNATarget^53^. The number of predicted targets for each miRNA varied from one to seven. Five hundred and fifty target mRNAs were predicted to be regulated through cleavage and the rest were through translational repression (Supplementary Table S4). According to gene ontology, majority of the target mRNAs encoded proteins involved in molecular functions related to binding, catalytic activity, transporter activity and transcription factors (Supplementary Figure S8). Out of 284 predicted miRNAs, 37 miRNA candidates from 32 miRNA families have been previously annotated in various plants and their target genes were either predicted or well-studied in plant systems. Although, the target genes of conserved miRNAs were similar in different model systems, their functions in a particular system may vary. There were also some conserved miRNAs, which target different genes in different systems due to minute sequence variations^50, 54, 55^.

**Figure 4:**
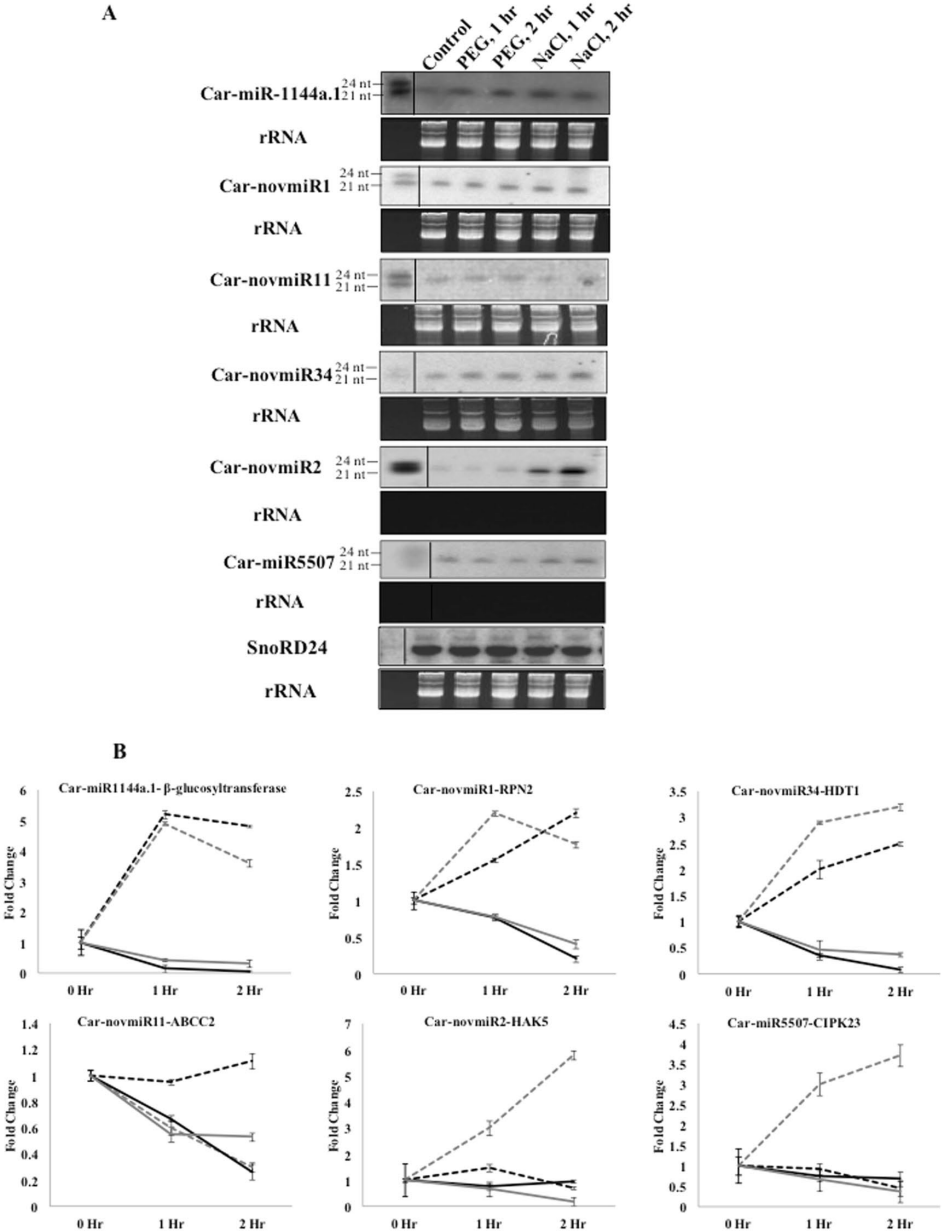
Expression patterns of selected miRNAs obtained by high throughput sequencing and their target genes. (**A**) Northern blot was performed to analyse expression levels of miRNAs at 2 mm region of chickpea root apex after 1 hr and 2 hrs of PEG and salt treatments. 15 µg of enriched small RNA from control and treated samples was loaded on denaturing (7 M urea) polyacrylamide (15%) gel. Radiolabeled antisense probes were used for hybridization. Ethidium bromide-stained small RNAs were shown for equal loading. SnoRD24 was used as control. Size markers (24 nt and 21 nt) were electrophoresed together with the experimental samples and separated before hybridization with marker-specific probes. (**B**) Expression pattern of the miRNAs shown in Fig. 6A and their predicted target genes in response to the same treatments as assessed by qRT-PCR. Standard deviations of three replicates were shown. *CaEF1α* was used as internal control for normalization. Grey and black lines represent salt and water deficit stress treatments, respectively. While dotted and solid lines represent miRNA and target gene, respectively.

**Figure 5:**
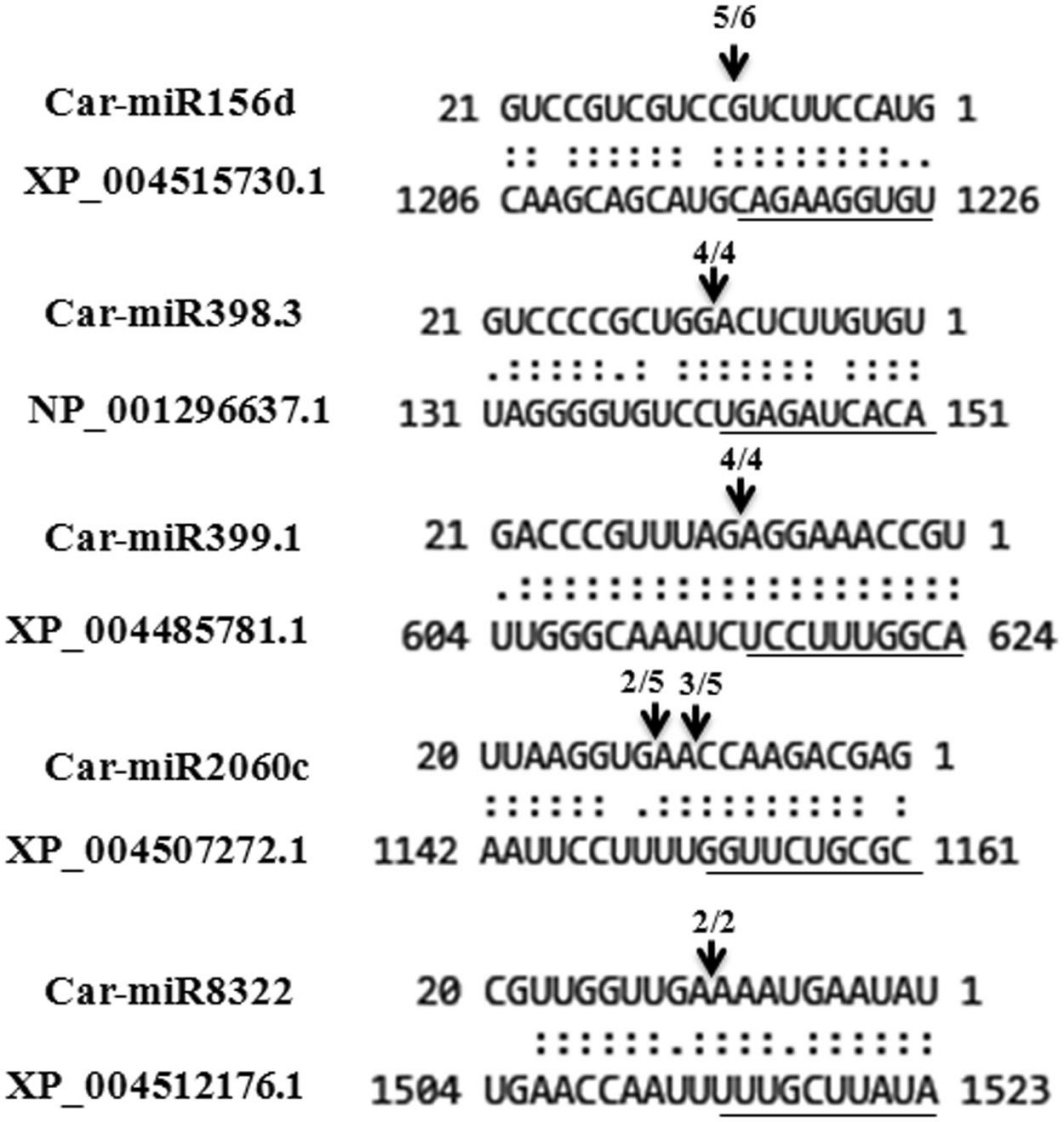
Determination of target gene cleavage site by degradome sequencing. Cleavage sites of six miRNA-target gene combinations are shown by aligning the corresponding mRNA ans miRNA sequences. Arrows mark the cleavage sites as determined by the sequencing of the 5′RLM-RACE products shown below in each case.

Based on high read counts, six miRNAs were selected for validation of expression profile by northern blots. Those are Car-novmiR1, Car-novmiR2, Car-novmiR11, Car-novmiR34, Car-miR1144a.1, Car-miR5507 (Fig. 4A). Their predicted targets were the mRNAs that encode *RPN2* (*HAPLESS6*) (XP_004495085.1), High affinity K +-transporter *HAK5* (XP_004508966.1), ABC transporter C2 (*ABCC2*) (XP_012568314.1), *HISTONE DEACETYLASE*1 (*HDT1*) (XP_004506131.1), 3′-O-beta*-GLUCOSYLTRANSFERASE* (XP_004512424.1) and CBL-interacting protein kinase 23 (*CIPK23*) (XP_004488520.1), respectively. Expression patterns of these target genes in the same samples, were assessed by quantitative RT-PCR (qRT-PCR). Expression patterns of all the six miRNAs in different stress conditions followed the same course of their digital expression patterns. Expression patterns of their target genes showed inverse correlations (Fig. 4B), suggesting that these miRNAs may affect the cellular processes regulated by these target genes during water deficit and high salinity. Chickpea gene expression profiles, as derived from previously reported root transcriptome in response to dehydration and salinity stresses^56^ were analyzed and compared with the expression profiles of the corresponding miRNAs. Expression profiles of transcripts corresponding to 242 target genes predicted in this study were obtained from that data. Of those, 203 transcripts showed inverse correlation of expression patterns to their corresponding miRNAs demonstrating a significant corroboration of the miRNA expression profile and the target genes predicted in this study (Supplementary Table S5). MicroRNAs cleave their target mRNAs at specific sites based on sequence complementarity. Accordingly, miRNA-target gene relationship can further be verified through sequencing of cleaved miRNAs or degradome. A limited degradome of 2 hr salt-treated root apex was amplified by 5′-RNA Ligase Mediated-RACE followed by sequencing of individual clones. Forty-one cleaved products belonging to 12 unique mRNAs were obtained and five of them corresponded with the target sites predicted in this study (Fig. 5). Those are miR156d-*GLABRA2* (XP_004515730.1), miR398-*SUPEROXIDE DISMUTASE* (*SOD*) (NP_001296637.1), and miR399-PHOSPAHTE OVERACCUMULATOR (*PHO2*) (XP_004485781.1), miR8322-*ASPARTIC PROTEINASE-LIKE Protein 1* (XP_004512176.1) and miR2060c-*Sodium-coupled Neutral AMINO ACID TRANSPORTER 6* (XP_004507272.1).

383 target mRNAs were predicted for 171 miRNAs, showing altered fold expression under stress treatments, out of which genes corresponding to 152 target mRNAs could be mapped on eight chromosomes. Maximum 38 genes were mapped on Chm 3, followed by Chm 1 (22) and Chm 4 (21). Targets genes of water deficit-responsive miRNAs were mostly clustered in chromosomes 4 and 7, while targets of salt-responsive miRNAs were mostly clustered on Chm 6 (Supplementary Figure S9).

### Validation of candidate miRNA-target genes in chickpea

**Figure 6:**
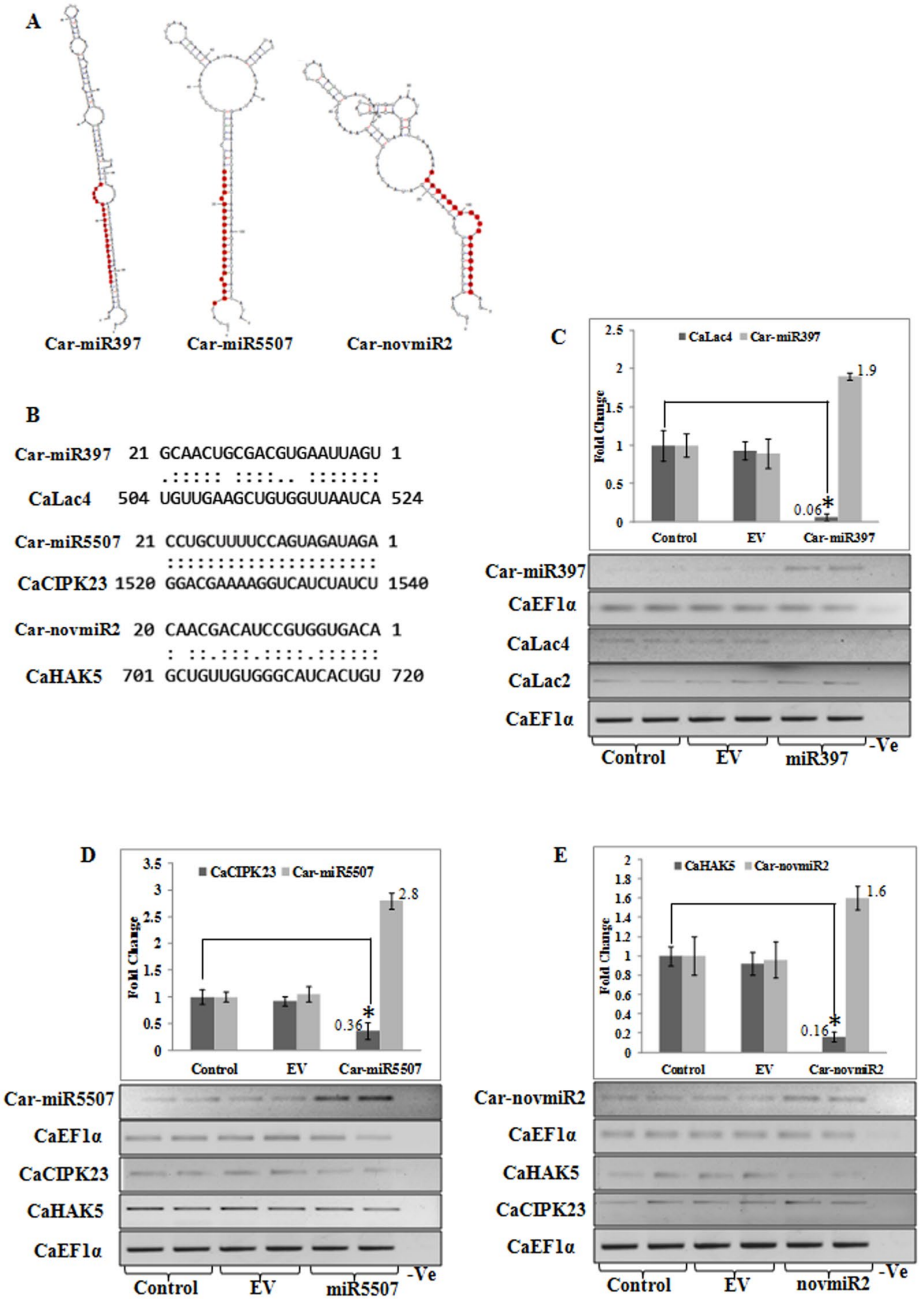
Validation of predicted miRNA targets. (**A**) Predicted secondary structures of miR397, miR5507 and novmiR2. (**B**) Predicted target sequences of miR397, miR5507 and novmiR2. (**C**–**E**) Candidate miRNA-target interaction was validated by transient over-expression of miRNA precursor sequence in chickpea leaf tissue by agaroinfiltration and change in expression pattern of their respective target genes was estimated by qRT-PCR as compared with control sample. The data represent three biological and three technical replicates for qRT-PCR and two independent samples were used for semi-quantitative RT-PCR. * indicates statistically significant change (p < 0.05) in expression.

As noted earlier, miRNA397 was upregulated in both stresses and was predicted to target *LACCASE4*, involved in lignin metabolism. Similarly, Car-novmiR2 and Car-miR5507 showing increased expression in late salt-treated sample were predicted to target high affinity potassium transporter *HAK5* and *CBL-INTERACTING PROTEIN KINASE 23* (*CIPK23*), respectively. Inverse correlation of expression patterns of these miRNAs and their respective target genes were also shown at transcript level. In order to validate candidate miRNA-target gene relationships, an *in vivo* transient expression system in chickpea was used. *LACCASE4*, *HAK5* and *CIPK23* genes were found to express in chickpea leaves also. Binary plasmids carrying precursor sequences of miR397, miR5507 and novmiR2 under the control of 35S promoter or an empty plasmid were agro-infiltrated in chickpea leaves. Stem-loop RT-PCR showed increased expression of the respective mature miRNAs in the agro-infiltrated leaves as compared to that in the non-infiltrated and empty vector control samples. Transcript levels of the corresponding target genes were found to be reduced significantly in three biological replicates (Fig. 6A–E), while expression of the non-target genes were unaltered. This expression assay showed that these cloned precursor transcripts were efficiently processed in native system to generate a functional mature miRNA, which were able to regulate their target mRNA levels.

## Discussion

Meristematic cells in the root apex and in the pericycle layer are the major determinants of developmental plasticity of root system in normal and adverse conditions. How plants integrate environmental cues to modulate RAM activity and ultimately root growth by miRNA-mediated mechanism is the key question in this study. Although, expression and ultimately the function of a gene is regulated at various steps, the post-transcriptional regulation of transcript level by miRNAs provides an additional layer of complexity in the mechanism of gene function. We have compared our result with the previously reported results from *Medicago* and soybean root tips under salt treatment^18, 25, 31^. Eleven miRNAs were found common between chickpea and each of *Medicago* (miR156, miR159, miR160, miR166, miR171, miR172, miR398, miR2592, miR2661, miR2672) and soybean (miR156, miR160, miR164, miR166, miR169, miR171, miR172, miR319, miR399, miR482, miR4412) root meristem tissues. Expression levels of miR172 and miR399a was found up- and downregulated, respectively, in all three legume RAM under salinity. However, some other miRNAs showed differential expression patterns such as, miR156 and miR160 expressions were up- and downregulated in chickpea and *Medicago*, respectively, in contrast to their opposite expression patterns in soybean. Expression of miR171 was upregulated in chickpea and *Medicago* in contrast to insignificant change in soybean. This differential miRNA expression patterns between chickpea, *Medicago* and soybean may have resulted due to different treatment procedures for example, normally grown chickpea and *Medicago* seedlings were treated with salt for a few hours while, soybean seedlings were germinated in presence of salt. Nevertheless, our result has revealed a complex regulatory mechanism of gene expression associated with root development and stress signaling.

Dynamics of miRNA expression under adverse soil condition and their predicted target genes in chickpea root apex indicated a marked influence of miRNA especially in root development and root hair formation. In general, the root epidermis is composed of a layer of root hair cells (trichoblast) followed by adjacent non-hair cell (atrichoblast) under normal condition however, under salt stress, this pattern changes^57–63^. Predicted target of Car-miR-156d, showing increased expression in salt-treated samples, was a mRNA that codes for a homeobox protein GLABRA2 (GL2), which expresses in root non-hair cells^64^. Inactivation of *GL2* gene was shown to alter differentiation pattern of root hairless epidermal cells to produce root hairs^57, 58^. Genes encoding proline-rich receptor-like protein kinase (*PERK13*) and extensin-2 were predicted as the targets of Car-miR-7031, upregulated by PEG treatment. *PERK13* expressed mostly in roots, especially in root hairs and was found to negatively regulate root hair elongation^62, 63^. Extensin-2, together with another protein LRX2, was shown to regulate root hair morphogenesis and elongation. *Lrx1* null mutant produce root hairs that frequently aborts, swells and branches^65^. It appears from the expression profiles of these miRNAs and their expected effects on target gene expressions that high salinity and water deficit cause downregulation of the negative regulators and upregulation of positive regulators of polarized growth of root hair through miRNA-mediated mechanism. Cell fate changes in the root epidermis and subsequent re-patterning of trichoblasts and atrichoblasts in response to salt stress has been reported before^66^.

Three miRNAs, Car-miR-361.1, Car-miR615 and Car-miR6562.1, which were predicted to target genes associated with lateral root formation such as, *LATERAL ORGAN BOUNDARIES* (*LOB*)^67^ and two ABC transporter genes (*ABCG* and PGP19)^59–61, 68^, exhibited increased expression in the stress-treated libraries. On the other hand, Car-miR1532, which was predicted to target *ARGINASE 1* and *ARGINASE 2*, displayed a reduced level of expression under PEG and salt treatments. Arginase 1 and -2 are critical regulators of nitric oxide signaling and were reported to negatively regulate auxin signaling and lateral root formation^69^. Expression patterns of these miRNAs indicated a negative regulation of lateral root formation in salinity. Reduction in the primary root growth and lateral root development are well-known responses to water deficit and salinity stresses^48, 70^.

Car-miR172e, Car-miR7907b.1 and Car-miRK12 showed increased expression in PEG- and salt-treated samples. These three miRNAs were predicted to target genes coding for DEAD-box ATP-dependent RNA helicases 24, -7 and -51, respectively. Loss-of-function mutations in two DEAD-box helicases *STRS1* and *STRS2* in *Arabidopsis* caused increased tolerance to salt, osmotic and heat stresses and induced expression of stress-responsive transcription factors^71–73^. In *Arabidopsis*, CIPK23 was shown to phosphorylate HAK5 to regulate HAK5-mediated high-affinity K^+^-uptake in roots^74^. Long exposure to high concentration of sodium chloride leads to repression of HAK5 transporter resulting in K^+^-deficiency in the root epidermal cells^75, 76^. Together with Na^+^ accumulation K^+^-deficiency causes a decrease in K^+^/Na^+^ ratio in the cytosol and subsequently affects plant growth^76–78^. Increased expression of Car-novmiR2 and Car-miR5507 and decreased level of their validated target mRNAs, *HAK5* and *CIPK23*, respectively, suggested a down regulation of *HAK5* expression and activity and provided a new insight in significant role of miRNA in high affinity potassium uptake mechanism under salinity in chickpea root.

The results described in this study would expand the candidate miRNAs and target genes to study regulation of root development under abiotic stresses. Salt treatment has been shown to increase auxin level in soybean root tip^31^. This suggests that RAM activity is directly regulated by phytohormones in response to environmental signals and the root tissues above RAM might also be affected by this response as phytohormones and miRNA can travel to different parts of the root. Many of the miRNAs identified in this study possess auxin- and abiotic stress-responsive *cis*-element in their promoters (Supplementary Table S6) suggesting that they can be directly regulated by the counteracting phytohormones. Our data provides the a genome-wide view of miRNA expression pattern in the cell division zone of chickpea root apex under water deficit and salinity stresses and probable miRNA-mediated regulation of cellular processes.

## Methods

### Plant materials, RNA isolation, small RNA enrichment and sequencing

Six-day post germination (dpg) seedlings of chickpea (*C. arientanum*; PUSABGD72) grown in growth chamber under suitable condition (temperature 23–25 °C and photoperiod 14 hrs light/10 hrs dark) were treated with 20% poly-ethyleneglycol (PEG-8000) and 250 mM NaCl for 1 hr and 2 hrs by submerging the roots. The control sample was treated with water for 2 hrs. For expression analysis and small RNA library construction, fifty number of 2 mm root tip regions for each treatment was excised with a scalpel having twin blade separated by 2 mm. For histogram, 3 mm root tips from control and treated samples were excised from 6 dpg chickpea seedlings and were processed as described before^79^. After fixation and wax embedding, 6 µm sections were sliced with a microtome (Leica#RM2265, Wetzlar, Germany). Saffrainin stained median-sections were observed under Carl Zeiss (Obercochen, Germany) axio imager 2 microscope.

Total RNA was extracted from harvested samples using TRI reagent (Sigma, St. Louis, MO, USA). RNA was quantified by NanoDrop1000 (Thermo Fisher Scientific, MA, USA). RNA-integrity was checked by separating in 1.5% denaturing agarose gel. Enrichment of small RNA was performed using PEG as described earlier^80, 81^. Small RNA (smRNA) libraries were prepared with miRCat small RNA cloning kit (Integrated DNA technologies, Coralville, US) according to manufacturer’s protocol. A concatanated small RNA library was prepared as described by Klevebring *et al*.^45^. These concatamers were sequenced using Roche 454 FLX system. The detail method is provided in Supplementary text S1.

### miRNA prediction and analysis

After filtering out adaptor sequences, the unique reads of 18–24 nt size range were processed for computational analysis. The valid reads were first filtered for rRNA/tRNA/snoRNA sequences and remaining sequences were mapped onto the chickpea genome assemblies^33, 82^ using Bowtie (version 1.1.2)^83^ with zero mismatch. The matched reads were screened for similarity with miRNA sequences present in miRBase (release 21)^84^ by allowing 2 mismatches with 2 gap penalty. The matched sequences were considered as known miRNAs. Rest of the sequences were used for novel miRNA prediction. Putative precursor sequences of an optimal size (150 bp up- and down-stream) were extracted for each remaining read^85^. Fold back secondary structures were generated with mfold web server (http://unafold.rna.albany.edu/), using the Zuker algorithm^86^ for prediction of novel miRNAs. To analyze miRNA expressions, reads per million (RPM) of total valid reads in a library was calculated. Normalized RPM *i.e*. RPM values of a miRNA normalized by the highest RPM value of that particular miRNA in any library, was used as fold expression to generate heat map. The heat map showing fold expression and hierarchical clustering were performed using TIGR Multi Experiment Viewer (MeV v4.9)^87^. Genomic positions of mapped miRNA sequences were used as input data for Mapchart 2.2^88^ for chromosomal map preparation.

### Target prediction

Potential targets for each miRNA was predicted by psRNAtarget web server (http://plantgrn.noble.org/psRNATarget/), using chickpea transcriptome and draft genome sequences^33, 89^ as reference. For minimum false positives following criteria were chosen: maximum expectation 3.0 (range 0–5.0), length for complementarity scoring (hspsize) 18 (range 15–30 bp), target accessibility i.e. allowed maximum energy to unpair the target site (UPE) was 25.0 (range 0–100, less is better). Flanking length around target site for target accessibility analysis was kept 17 bp in upstream and 13 bp in downstream and range of central mismatch leading to translational inhibition was 9–11 nts^53^. Functional annotation of predicted target genes was done by Blast2GO programme^90^ using *Arabidopsis* non-redundant database from TAIR as reference.

### Expression analysis

For northern blot analysis, 15 µg of enriched smRNA was separated on denaturing (7 M urea) 15% polyacrylamide gel along with the size markers (24 nt and 21 nt) and was transferred on Hybond-N+ membrane (Amershem Biosciences, Buckinghamshire, UK). Membrane was UV-crosslinked, the size marker lane was separated and individually hybridized with radiolabeled size marker-specific and miRNA-specific probes. SnoRND24 was used as an internal control. Expression analysis of miRNAs and their respective target genes was done by quantitative reverse transcriptase PCR (qRT-PCR). For miRNAs stem-loop primers were designed and cDNA for individual miRNA was prepared by pulse-RT reaction using SuperScript^®^ III Reverse Transcriptase (Thermo Fisher Scientific)^91, 92^. Details of method is provided in Supplementary text S1 *. ELONGATION FACTOR 1-α* (*EF-1α*) gene was used as internal control for normalization. Also qRT-PCR results were validated by northern blotting of miRNAs with specific probes and semi-quantitative RT-PCR of target genes. Relative expressions of genes were measured on the basis of signal intensity by using QuantityOne (BioRad laboratories, Berkeley, CA). Sequences of miRNA anti-sense oligos used for northern blots, adapters and primers used for library preparation and RT-PCR are given in detail in Supplementary Table S7.

### Degradome analysis and validation of candidate miRNA-target relationship

Validation of miRNA mediated cleavage of target mRNA was performed by RNA Ligase mediated- Rapid amplification of cDNA ends (RLM-RACE) method as described by Llave *et al*.^93^, Details of used method are given in Supplementary text S1. Obtained sequences were mapped to chickpea transcripts by NCBI-BLAST tool (http://blast.ncbi.nlm.nih.gov/Blast.cgi). miRNA precursor sequences were amplified from chickpea genomic DNA using primers listed in Supplementary Table S7 and cloned in to pEarlyGate100 vector. The plasmids with or without miRNA precursors were agroinfiltrated in to young chickpea leaves as described by Sparkes *et al*.^94^, and miRNA and target gene expression was determined by qRT-PCR. Details of used method are given in Supplementary text S1.

## Electronic supplementary material

Supplementary Informations
