# Supplementary material for: MicroRNA profiling provides insights into post-transcriptional regulation of gene expression in chickpea root apex under salinity and water deficiency
Source: Sci Rep. 2017 Jul 5;7:4632. doi: 10.1038/s41598-017-04906-z (PMC5498500; doi:10.1038/s41598-017-04906-z)
Supplement: Supplementary file 1 — Supplementary Informations [file 41598_2017_4906_MOESM1_ESM.pdf]

Supplementary information

MicroRNA profiling provides insights into post-transcriptional regulation of gene expression in chickpea root apex under salinity and water deficiency

Hitaishi Khandal, Sabiha Parween, Riti Roy, Mukesh Kumar Meena, Debasis Chattopadhyay*

Institution: National Institute of Plant Genome Research, Aruna Asaf Ali Marg, New Delhi 110067, India

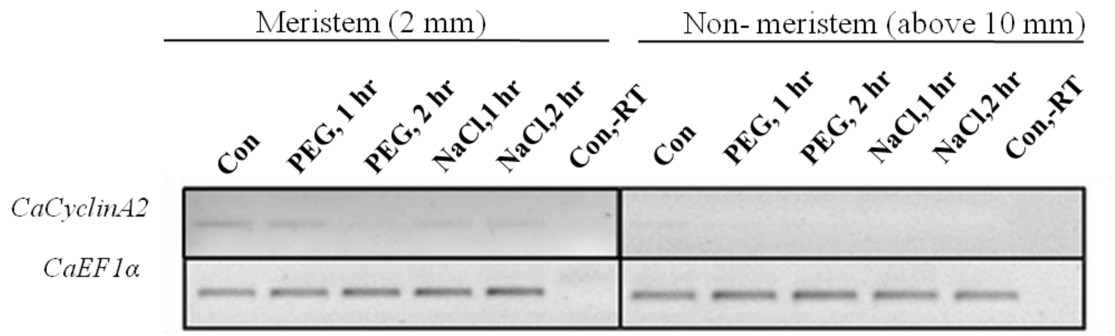

Supplementary Fig. S1- Expression level of *CYCLINA2*, estimated by semi quantitative RT-PCR (32 cycles), in 2 mm region of root apex and in the same size of tissue above 10 mm of root apex. Chickpea elongation factor 1 α (*CaEF1α*) was used as internal control. A reaction (Con,-RT) was performed without using reverse transcriptase to show absence of genomic DNA contamination.

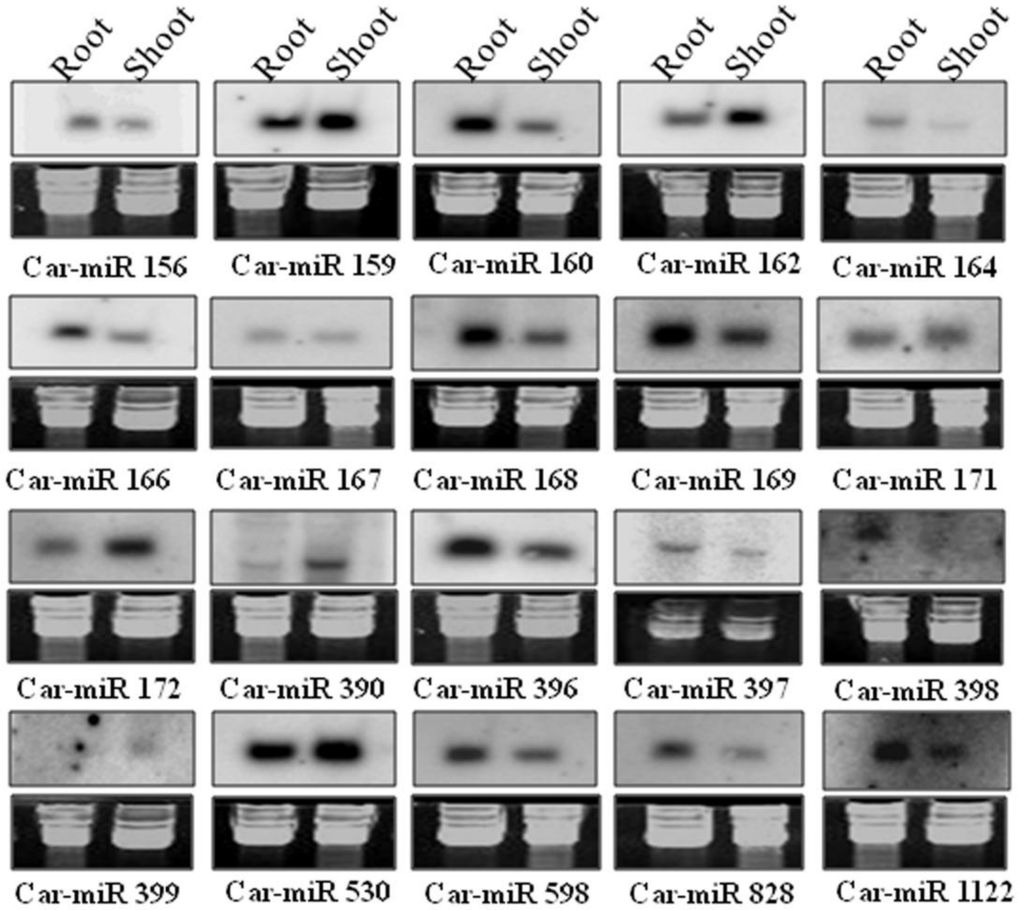

Supplementary Fig. S2- Expression pattern of representative members from each miRNA family in chickpea root and shoot tissues. Northern blot was performed to analyse expression levels of miRNAs in chickpea root and shoot tissues. 15µg of enriched smallRNA from control root and shoot tissues was loaded on denaturing (7M urea) polyacrylamide (15%) gel. Radiolabeled antisense probes were used for hybridization. Ethidium bromide-stained small RNAs were shown for equal loading.

Sample	Read Count
Control (RL1)	84162
PEG 1Hr (RL2)	316179
PEG 2Hr (RL3)	191261
NaCl 1Hr (RL4)	70779
NaCl 2Hr (RL5)	140878
Total	803259

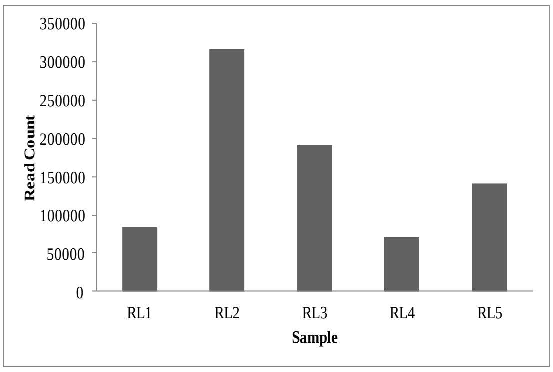

Supplementary Fig. S3- Total valid read counts obtained from each library. Quality filtered total raw reads obtained from 454 sequencing in control and PEG/Salt treated libraries.

A

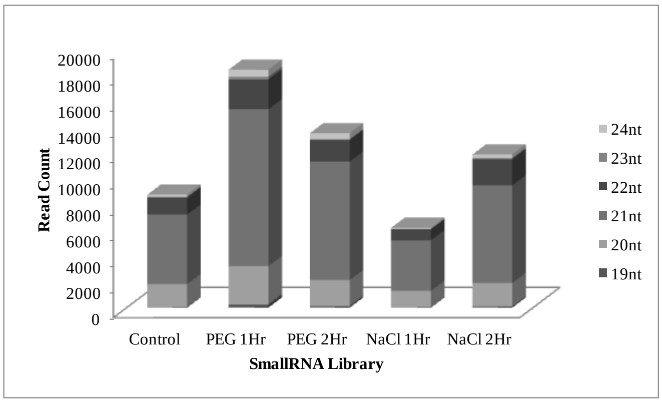

B

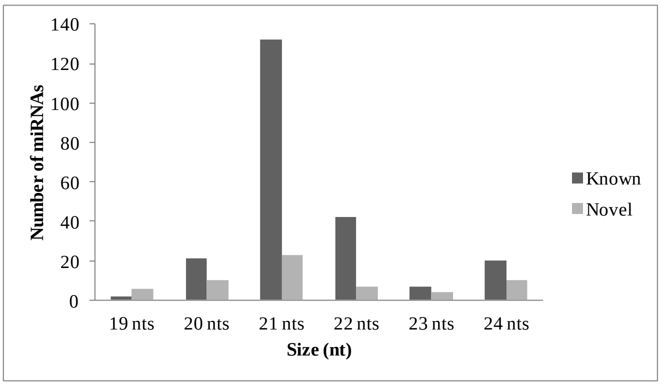

Supplementary Fig. S4- Size distribution of small RNA reads and predicted miRNAs. (A) Size distribution of small RNA reads in the size range of 18-24 nt in five small RNA libraries. Number of high quality filtered reads obtained after adapter removal from five libraries are shown. (B) Size distribution of the predicted known and novel miRNAs.

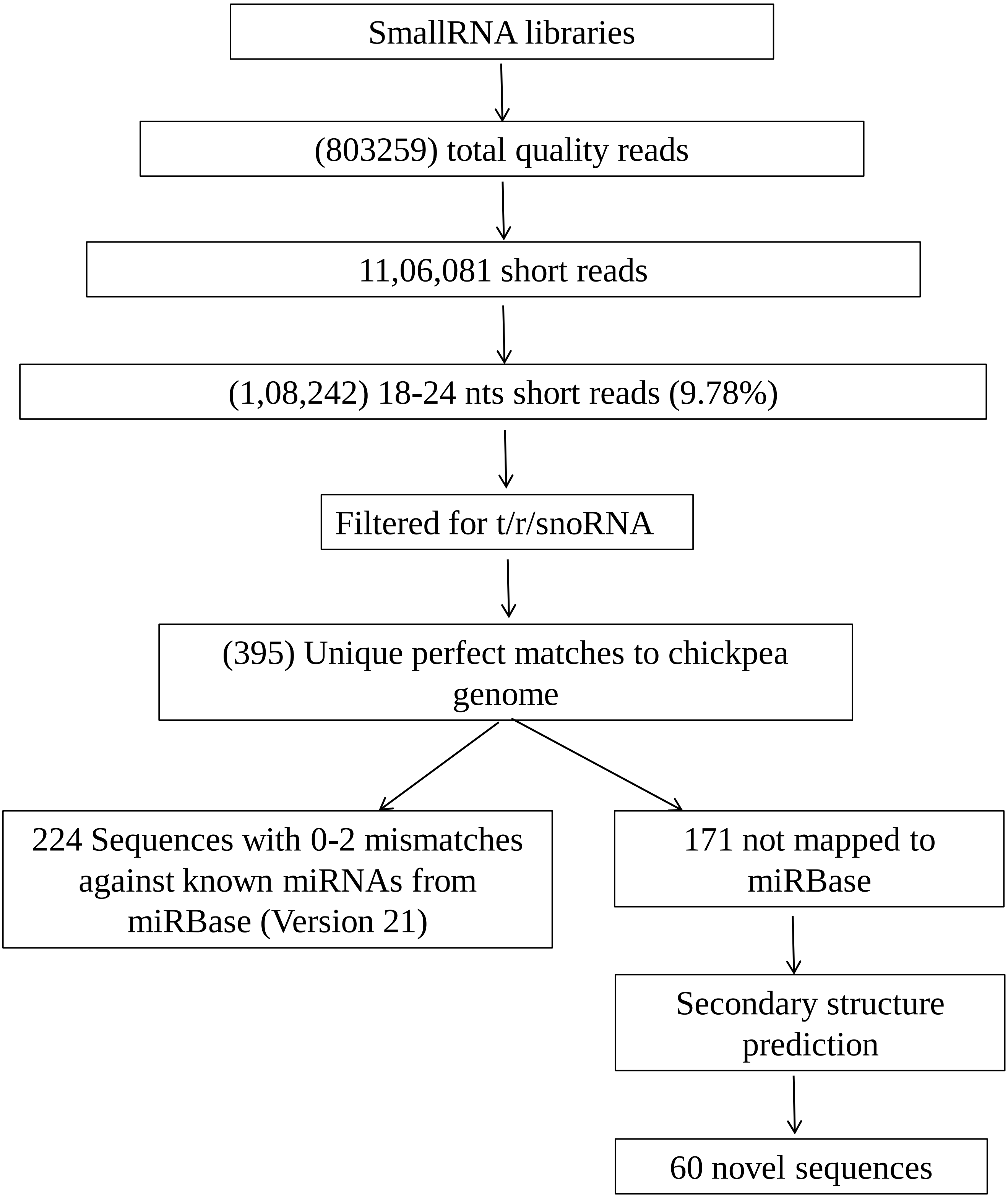

Supplementary Fig. S5- Analysis pipeline for miRNA prediction

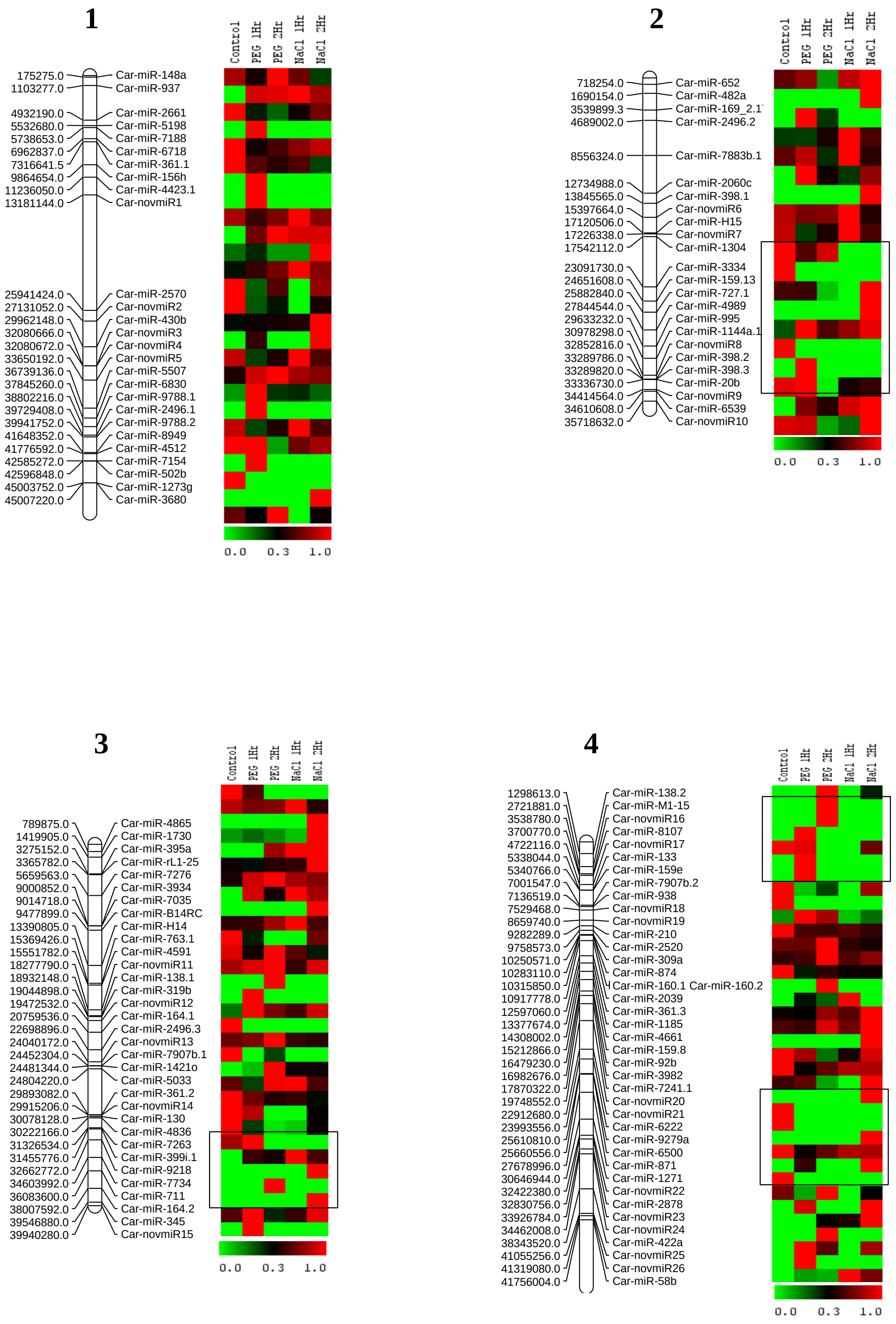
1
Control PEG 1Hr PEG 2Hr NaCl 1Hr NaCl 2Hr
175275.0 Car-miR-148a
1103277.0 Car-miR-937
4932190.0 Car-miR-2661
5532680.0 Car-miR-5198
5738653.0 Car-miR-7188
6962837.0 Car-miR-6718
7316641.5 Car-miR-361.1
9864654.0 Car-miR-156h
11236050.0 Car-miR-4423.1
13181144.0 Car-novmiR1
25941424.0 Car-miR-2570
27131052.0 Car-novmiR2
29962148.0 Car-miR-430b
32080666.0 Car-novmiR3
32080672.0 Car-novmiR4
33650192.0 Car-novmiR5
36739136.0 Car-miR-5507
37845260.0 Car-miR-6830
38802216.0 Car-miR-9788.1
39729408.0 Car-miR-2496.1
39941752.0 Car-miR-9788.2
41648352.0 Car-miR-8949
41776592.0 Car-miR-4512
42585272.0 Car-miR-7154
42596848.0 Car-miR-502b
45003752.0 Car-miR-1273g
45007220.0 Car-miR-3680
0.0 0.3 1.0
2
Control PEG 1Hr PEG 2Hr NaCl 1Hr NaCl 2Hr
718254.0 Car-miR-652
1690154.0 Car-miR-482a
3539899.3 Car-miR-169_2.1
4689002.0 Car-miR-2496.2
8556324.0 Car-miR-7883b.1
12734988.0 Car-miR-2060c
13845565.0 Car-miR-398.1
15397664.0 Car-novmiR6
17120506.0 Car-miR-H15
17226338.0 Car-novmiR7
17542112.0 Car-miR-1304
23091730.0 Car-miR-3334
24651608.0 Car-miR-159.13
25882840.0 Car-miR-727.1
27844544.0 Car-miR-4989
29633232.0 Car-miR-995
30978298.0 Car-miR-1144a.1
32852816.0 Car-novmiR8
33289786.0 Car-miR-398.2
33289820.0 Car-miR-398.3
33336730.0 Car-miR-20b
34414564.0 Car-novmiR9
34610608.0 Car-miR-6539
35718632.0 Car-novmiR10
0.0 0.3 1.0
3
Control PEG 1Hr PEG 2Hr NaCl 1Hr NaCl 2Hr
789875.0 Car-miR-4865
1419905.0 Car-miR-1730
3275152.0 Car-miR-395a
3365782.0 Car-miR-rL1-25
5659563.0 Car-miR-7276
9000852.0 Car-miR-3934
9014718.0 Car-miR-7035
9477899.0 Car-miR-B14RC
13390805.0 Car-miR-H14
15369426.0 Car-miR-763.1
15551782.0 Car-miR-4591
18277790.0 Car-novmiR11
18932148.0 Car-miR-138.1
19044898.0 Car-miR-319b
19472532.0 Car-novmiR12
20759536.0 Car-miR-164.1
22698896.0 Car-miR-2496.3
24040172.0 Car-novmiR13
24452304.0 Car-miR-7907b.1
24481344.0 Car-miR-1421o
24804220.0 Car-miR-5033
29893082.0 Car-miR-361.2
29915206.0 Car-novmiR14
30078128.0 Car-miR-130
30222166.0 Car-miR-4836
31326534.0 Car-miR-7263
31455776.0 Car-miR-399i.1
32662772.0 Car-miR-9218
34603992.0 Car-miR-7734
36083600.0 Car-miR-711
38007592.0 Car-miR-164.2
39546880.0 Car-miR-345
39940280.0 Car-novmiR15
0.0 0.3 1.0
4
Control PEG 1Hr PEG 2Hr NaCl 1Hr NaCl 2Hr
1298613.0 Car-miR-138.2
2721881.0 Car-miR-M1-15
3538780.0 Car-novmiR16
3700770.0 Car-miR-8107
4722116.0 Car-novmiR17
5338044.0 Car-miR-133
5340766.0 Car-miR-159e
7001547.0 Car-miR-7907b.2
7136519.0 Car-miR-938
7529468.0 Car-novmiR18
8659740.0 Car-novmiR19
9282289.0 Car-miR-210
9758573.0 Car-miR-2520
10250571.0 Car-miR-309a
10283110.0 Car-miR-874
10315850.0 Car-miR-160.1 Car-miR-160.2
10917778.0 Car-miR-2039
12597060.0 Car-miR-361.3
13377674.0 Car-miR-1185
14308002.0 Car-miR-4661
15212866.0 Car-miR-159.8
16479230.0 Car-miR-92b
16982676.0 Car-miR-3982
17870322.0 Car-miR-7241.1
19748552.0 Car-novmiR20
22912680.0 Car-novmiR21
23993556.0 Car-miR-6222
25610810.0 Car-miR-9279a
25660556.0 Car-miR-6500
27678996.0 Car-miR-871
30646944.0 Car-miR-1271
32422380.0 Car-novmiR22
32830756.0 Car-miR-2878
33926784.0 Car-novmiR23
34462008.0 Car-novmiR24
38343520.0 Car-miR-422a
41055256.0 Car-novmiR25
41319080.0 Car-novmiR26
41756004.0 Car-miR-58b
0.0 0.3 1.0

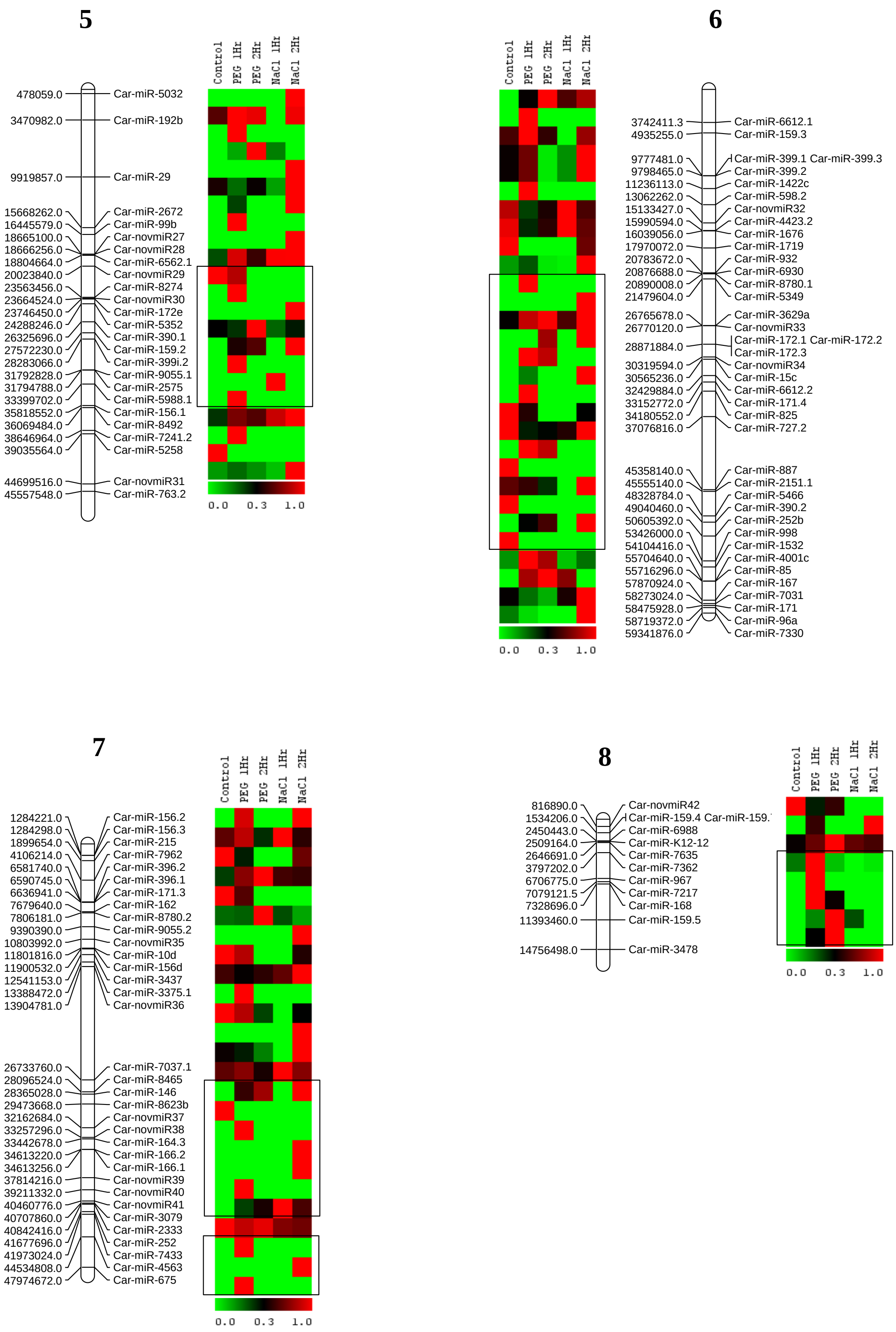

Supplementary Fig. S6- Distribution of miRNAs on eight chickpea chromosomes. Chromosomal positioning of miRNAs on eight chickpea chromosomes and their expression profiles in different libraries presented as heat maps. Boxes are used to mark the miRNA clusters showing significant alteration in different periods of stresses.

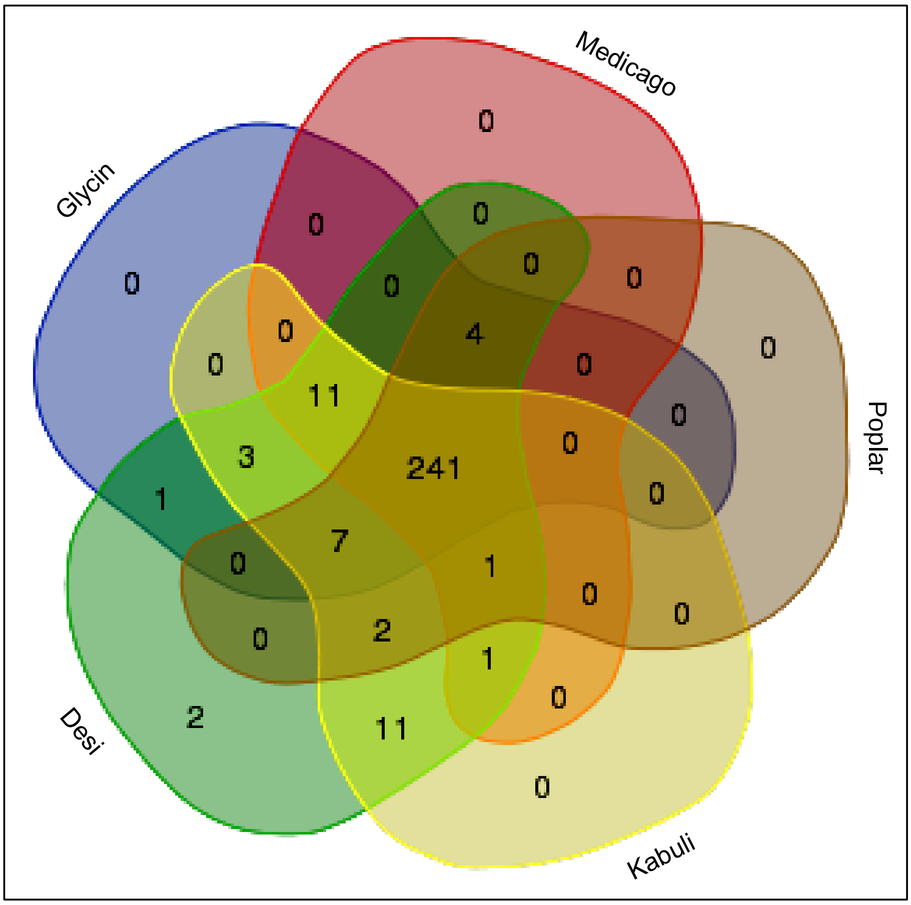

Supplementary Fig. S7- Venn diagram representing distribution of predicted miRNAs in *Medicago trancatula*, *Populus trichocarpa* (Poplar), Glycin max, *Cicer arietinum* (*Desi* and *Kabuli*).

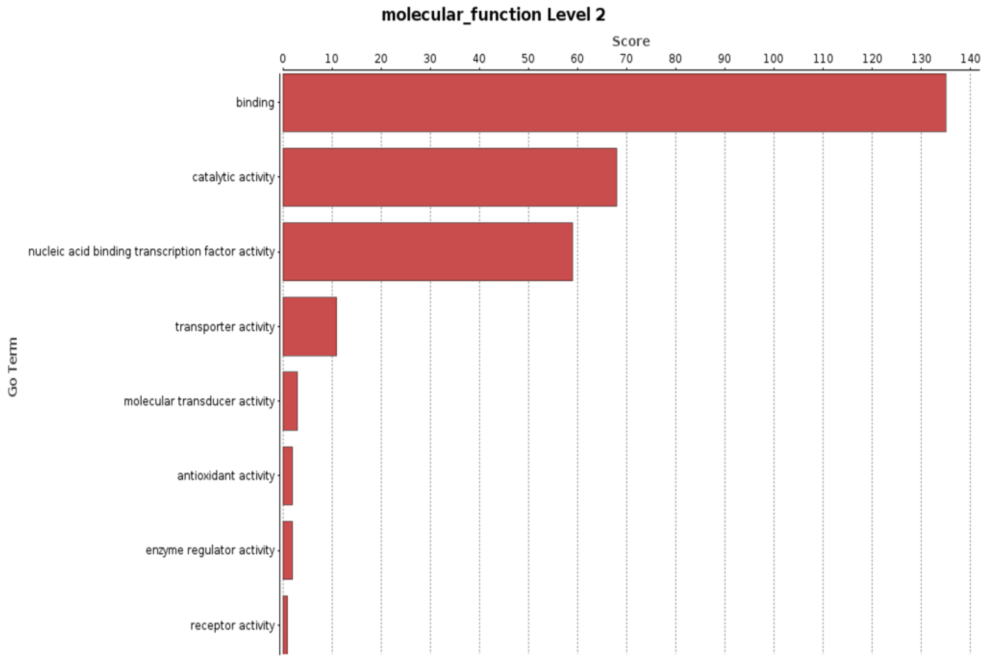

Supplementary Fig. S8- Gene ontology annotation for predicted target genes.

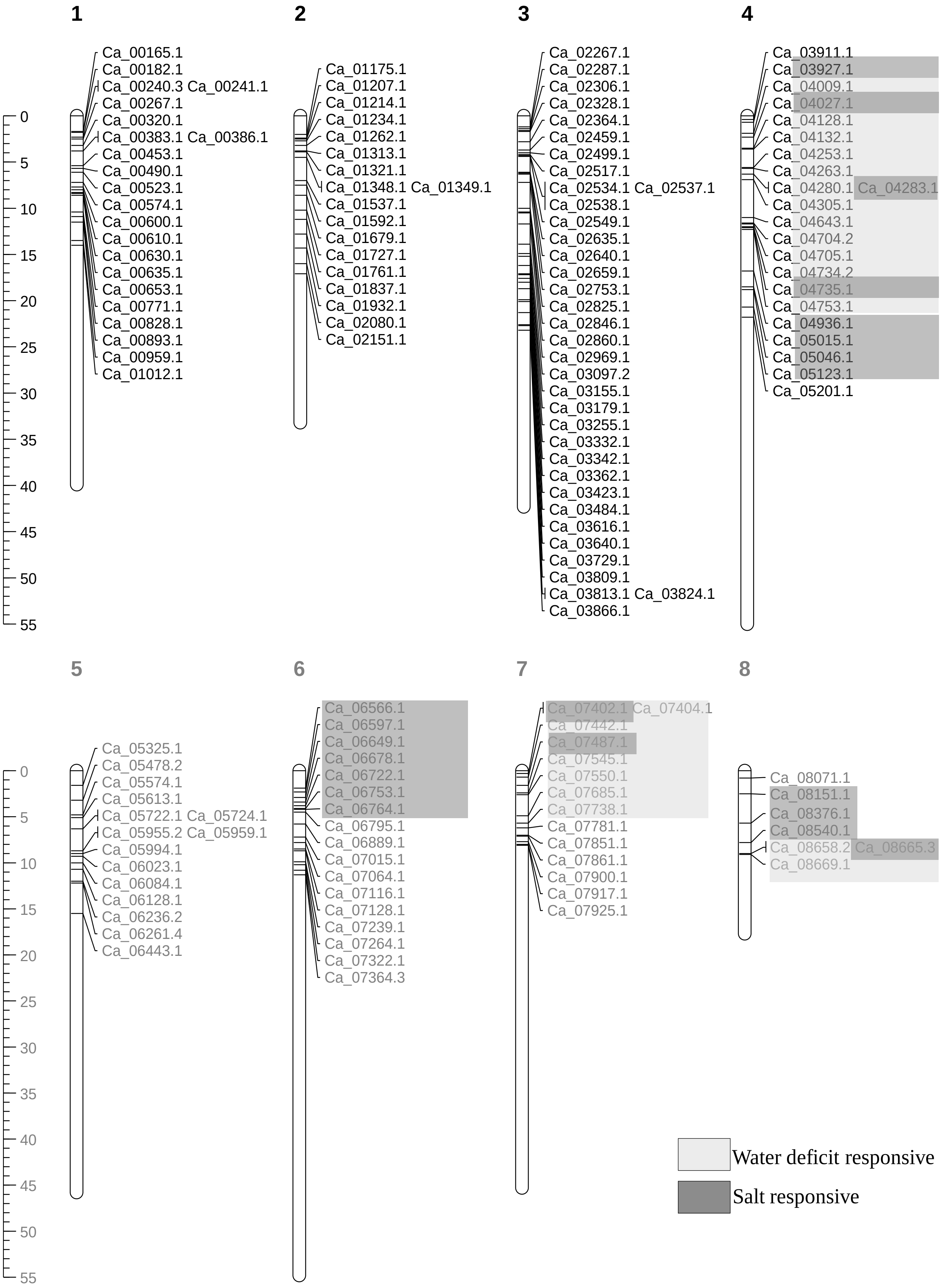

Supplementary Fig. S9- Chromosomal mapping of predicted target genes of stress-responsive miRNAs. Light-shaded and dark-shaded boxes represent the target genes of water-deficit-responsive and salt-responsive miRNAs, respectively.

Supplementary Table S1- Predicted targets of genome annotated conserved miRNAs.

miRNA_Acc.	Target_Acc.	NCBI_Acc.	Target Description
**Car-miR-156.2**	Ca_06849.1|Ca_LG_6:5035182-5037851|plus|	gi|828318259|ref|XP_004504410.2|	Squamosa promoter-binding-like protein 2
**Car-miR-156.2**	Ca_05690.1|Ca_LG_5:5939353-5941887|plus|	gi|502133312|ref|XP_004501715.1|	Squamosa promoter-binding-like protein 13
**Car-miR-156.2**	Ca_18054.1|scaffold02999:4085-9494|minus|	gi|502122822|ref|XP_004497906.1|	Squamosa promoter-binding-like protein 9
**Car-miR-156.2**	Ca_07357.1|Ca_LG_6:11254583-11258735|plus|	gi|502139372|ref|XP_004503743.1|	Squamosa promoter-binding-like protein 13
**Car-miR-156.2**	Ca_09722.1|scaffold00827:36897-42020|minus|	gi|502087112|ref|XP_004488431.1|	Squamosa promoter-binding-like protein 6
**Car-miR-156.3**	Ca_12773.1|scaffold00473:42673-44784|minus|	gi|502135909|ref|XP_004502495.1|	BTB/POZ domain-containing protein At1g21780
**Car-miR-159.2**	Ca_03379.1|Ca_LG_3:17731202-17743486|plus|	gi|502160145|ref|XP_004511648.1|	SNARE-interacting protein KEULE
**Car-miR-159.3**	Ca_12156.1|scaffold00341:113366-114136|plus|	gi|502168039|ref|XP_004514305.1|	Transcription factor GAMYB
**Car-miR-159.3**	Ca_03033.1|Ca_LG_3:12555780-12557590|minus|	gi|502106699|ref|XP_004493026.1|	Transcription factor GAMYB
**Car-miR-159.3**	Ca_17081.2|scaffold02188:38856-43752|plus|	gi|502179911|ref|XP_004516524.1|	Transcription factor GAMYB
**Car-miR-159.4**	Ca_17081.2|scaffold02188:38856-43752|plus|	gi|502179911|ref|XP_004516524.1|	Transcription factor GAMYB
**Car-miR-159.5**	Ca_14341.1|scaffold00872:6471-7722|plus|	gi|828326163|ref|XP_012573637.1|	Myb81, putative
**Car-miR-159.5**	Ca_12014.1|scaffold00316:160539-162063|minus|	gi|828328031|ref|XP_004510118.2|	Transcription factor GAMYB
**Car-miR-159.5**	Ca_17081.2|scaffold02188:38856-43752|plus|	gi|502179911|ref|XP_004516524.1|	Transcription factor GAMYB
**Car-miR-159.5**	Ca_10993.1|scaffold00126:159321-162261|plus|	gi|502079474|ref|XP_004486271.1|	Putative uncharacterized protein
**Car-miR-159.5**	Ca_14123.1|scaffold00807:68259-70266|plus|	gi|502097520|ref|XP_004491014.1|	Peptide-N4-(N-acetyl-beta-glucosaminyl)asparagine amidase A
**Car-miR-159.5**	Ca_03478.1|Ca_LG_3:18643849-18644777|plus|	gi|828330481|ref|XP_012574439.1|	Osmotin-like protein OSM34
**Car-miR-159.5**	Ca_17291.1|scaffold02337:26407-29767|plus|	gi|502089895|ref|XP_004489054.1|	Putative uncharacterized protein
**Car-miR-159.7**	Ca_17081.2|scaffold02188:38856-43752|plus|	gi|502179911|ref|XP_004516524.1|	Transcription factor GAMYB
**Car-miR-159.8**	Ca_08191.1|Ca_LG_8:3896989-3900461|plus|	gi|502150677|ref|XP_004508070.1|	Chromatin structure-remodeling complex protein BSH
**Car-miR-159.8**	Ca_16914.2|scaffold02095:47553-50151|minus|	gi|502082466|ref|XP_004487176.1|	Transcription factor TCP4
**Car-miR-159.8**	Ca_10515.1|scaffold03726:52436-55174|minus|	gi|502155347|ref|XP_004510040.1|	Transcription factor TCP4
**Car-miR-159.13**	Ca_14341.1|scaffold00872:6471-7722|plus|	gi|828326163|ref|XP_012573637.1|	Myb81, putative
**Car-miR-159.13**	Ca_03033.1|Ca_LG_3:12555780-12557590|minus|	gi|502106699|ref|XP_004493026.1|	Transcription factor GAMYB
**Car-miR-160.1**	Ca_15821.2|scaffold01444:60135-63748|minus|	gi|828337470|ref|XP_012567350.1|	Auxin response factor 17
**Car-miR-160.1**	Ca_13087.1|scaffold00538:49721-52061|minus|	gi|502156787|ref|XP_004510646.1|	Auxin response factor 18
**Car-miR-160.1**	Ca_13471.1|scaffold00625:20206-23322|minus|	gi|357443233|ref|XP_003591894.1|	Auxin response factor 18
**Car-miR-160.1**	Ca_00910.1|Ca_LG_1:11950543-11953447|minus|	gi|502082215|ref|XP_004487099.1|	Auxin response factor 10
**Car-miR-160.1**	Ca_06444.1|Ca_LG_5:15587817-15591576|plus|	gi|502145397|ref|XP_004506012.1|	Auxin response factor 18
**Car-miR-160.2**	Ca_15821.2|scaffold01444:60135-63748|minus|	gi|828337470|ref|XP_012567350.1|	Auxin response factor 17
**Car-miR-160.2**	Ca_13087.1|scaffold00538:49721-52061|minus|	gi|502156787|ref|XP_004510646.1|	Auxin response factor 18
**Car-miR-160.3**	Ca_15821.2|scaffold01444:60135-63748|minus|	gi|828337470|ref|XP_012567350.1|	Auxin response factor 17
**Car-miR-160.3**	Ca_13087.1|scaffold00538:49721-52061|minus|	gi|502156787|ref|XP_004510646.1|	Auxin response factor 18
**Car-miR-162**	Ca_02200.1|Ca_LG_3:681237-692327|plus|	gi|502114170|ref|XP_004494884.1|	Endoribonuclease Dicer homolog 1
**Car-miR-162**	Ca_15093.1|scaffold01150:66433-70507|plus|	gi|502090685|ref|XP_004489308.1|	Peroxidase superfamily protein

**Car-miR-162**	Ca_06658.1|Ca_LG_6:3006169-3010452|minus|	gi|502138810|ref|XP_004503536.1|	Inactive protein kinase SELMODRAFT_444075
**Car-miR-164.1**	Ca_12828.1|scaffold00481:47272-58337|minus|	gi|729346233|ref|XP_010541854.1|	Alanine--tRNA ligase
**Car-miR-164.1**	Ca_14826.1|scaffold01044:26439-30711|minus|	gi|502079330|ref|XP_004486227.1|	Putative MO25-like protein At5g47540
**Car-miR-164.2**	Ca_03386.1|Ca_LG_3:17792338-17793875|plus|	gi|502160115|ref|XP_004511638.1|	Putative DNA-binding protein ESCAROLA
**Car-miR-164.3**	Ca_14182.1|scaffold00822:55453-57393|minus|	gi|502089208|ref|XP_004488843.1|	NAC domain-containing protein 21/22
**Car-miR-164.3**	Ca_00673.1|Ca_LG_1:8963833-8965919|minus|	gi|502088135|ref|XP_004488746.1|	Protein CUP-SHAPED COTYLEDON 2
**Car-miR-164.3**	Ca_08467.1|Ca_LG_8:6578835-6580299|minus|	gi|502152022|ref|XP_004508725.1|	NAC domain-containing protein 100
**Car-miR-166.2**	Ca_09565.1|scaffold00659:93247-99756|minus|	gi|502156053|ref|XP_004510296.1|	Homeobox-leucine zipper protein ATHB-15
**Car-miR-166.2**	Ca_00550.2|Ca_LG_1:6395795-6399868|plus|	gi|502080067|ref|XP_004486450.1|	Homeobox-leucine zipper protein ATHB-14
**Car-miR-166.2**	Ca_00800.1|Ca_LG_1:10647803-10649528|minus|	gi|828290193|ref|XP_004487500.2|	ABSCISIC ACID-INSENSITIVE 5-like protein 1
**Car-miR-167**	Ca_22464.1|scaffold174698:103-450|minus|	gi|828312512|ref|XP_012571461.1|	Sec14p-like phosphatidylinositol transfer family protein
**Car-miR-167**	Ca_04643.1|Ca_LG_4:10985156-10989303|minus|	gi|502120037|ref|XP_004496826.1|	G-type lectin S-receptor-like serine/threonine-protein kinase At5g24080
**Car-miR-168**	Ca_17752.1|scaffold02690:19452-20602|minus|	gi|502177263|ref|XP_004516042.1|	Proteinase inhibitor type-2
**Car-miR-168**	Ca_20844.1|scaffold23940:357-1028|minus|	gi|491545072|ref|WP_005402688.1|	D-serine dehydratase
**Car-miR-168**	Ca_05907.1|Ca_LG_5:8163219-8170351|plus|	gi|502135658|ref|XP_004502409.1|	tRNA (cytosine(34)-C(5))-methyltransferase
**Car-miR-168**	Ca_10689.1|scaffold00077:84562-91887|minus|	gi|502109357|ref|XP_004493626.1|	Pentatricopeptide repeat-containing protein At2g22410
**Car-miR-169_2.1**	Ca_10488.1|scaffold02365:17548-21337|minus|	gi|502129138|ref|XP_004500196.1|	Nuclear transcription factor Y subunit A-1
**Car-miR-169_2.3**	Ca_10488.1|scaffold02365:17548-21337|minus|	gi|502129138|ref|XP_004500196.1|	Nuclear transcription factor Y subunit A-1
**Car-miR-169_2.4**	Ca_10488.1|scaffold02365:17548-21337|minus|	gi|502129138|ref|XP_004500196.1|	Nuclear transcription factor Y subunit A-1
**Car-miR-169_2.4**	Ca_02468.3|Ca_LG_3:3747798-3751888|minus|	gi|502112191|ref|XP_004494259.1|	Nuclear transcription factor Y subunit A-10
**Car-miR-169_2.5**	Ca_10488.1|scaffold02365:17548-21337|minus|	gi|502129138|ref|XP_004500196.1|	Nuclear transcription factor Y subunit A-1
**Car-miR-169_2.5**	Ca_02468.3|Ca_LG_3:3747798-3751888|minus|	gi|502112191|ref|XP_004494259.1|	Nuclear transcription factor Y subunit A-10
**Car-miR-169_2.6**	Ca_17176.1|scaffold02257:21257-24948|plus|	gi|502176812|ref|XP_004515944.1|	Protein of unknown function (DUF3411)
**Car-miR-169_2.6**	Ca_04058.1|Ca_LG_4:2563989-2566758|plus|	gi|502178163|ref|XP_004516191.1|	Cytochrome P450 94A1
**Car-miR-169_2.6**	Ca_19401.1|scaffold05572:1643-4474|plus|	gi|502165610|ref|XP_004513634.1|	Cytochrome P450 94A1
**Car-miR-169_2.7**	Ca_10488.1|scaffold02365:17548-21337|minus|	gi|502129138|ref|XP_004500196.1|	Nuclear transcription factor Y subunit A-1
**Car-miR-169_2.17**	Ca_10488.1|scaffold02365:17548-21337|minus|	gi|502129138|ref|XP_004500196.1|	Nuclear transcription factor Y subunit A-1
**Car-miR-171.2**	Ca_16636.1|scaffold01903:31452-34216|minus|	gi|502174226|ref|XP_004515465.1|	Scarecrow-like protein 6
**Car-miR-171.2**	Ca_18262.1|scaffold03260:12420-15412|plus|	gi|502146488|ref|XP_004506474.1|	Scarecrow-like protein 6
**Car-miR-171.3**	Ca_18262.1|scaffold03260:12420-15412|plus|	gi|502146488|ref|XP_004506474.1|	Scarecrow-like protein 6
**Car-miR-171.3**	Ca_16636.1|scaffold01903:31452-34216|minus|	gi|502174226|ref|XP_004515465.1|	Scarecrow-like protein 6
**Car-miR-171.4**	Ca_16636.1|scaffold01903:31452-34216|minus|	gi|502174226|ref|XP_004515465.1|	Scarecrow-like protein 6
**Car-miR-171.4**	Ca_18262.1|scaffold03260:12420-15412|plus|	gi|502146488|ref|XP_004506474.1|	Scarecrow-like protein 6
**Car-miR-171.5**	Ca_16636.1|scaffold01903:31452-34216|minus|	gi|502174226|ref|XP_004515465.1|	Scarecrow-like protein 6
**Car-miR-171.5**	Ca_18262.1|scaffold03260:12420-15412|plus|	gi|502146488|ref|XP_004506474.1|	Scarecrow-like protein 6
**Car-miR-172.1**	Ca_00883.1|Ca_LG_1:11416534-11420767|minus|	gi|502082378|ref|XP_004487148.1|	Ethylene-responsive transcription factor RAP2-7
**Car-miR-172.1**	Ca_01065.1|Ca_LG_2:593143-597426|minus|	gi|502145380|ref|XP_004506004.1|	Ethylene-responsive transcription factor RAP2-7
**Car-miR-172.1**	Ca_02644.1|Ca_LG_3:6171289-6174150|plus|	gi|502111031|ref|XP_004493938.1|	Floral homeotic protein APETALA 2
**Car-miR-172.1**	Ca_11972.1|scaffold00308:10342-13197|plus|	gi|502152836|ref|XP_004509117.1|	Floral homeotic protein APETALA 2
**Car-miR-172.1**	Ca_03431.1|Ca_LG_3:18117967-18121653|plus|	gi|502160801|ref|XP_004511904.1|	Floral homeotic protein APETALA 2
**Car-miR-172.1**	Ca_20442.1|scaffold12301:3162-3527|plus|	gi|357496583|ref|XP_003618580.1|	F-box/FBD/LRR-repeat protein

**Car-miR-172.1**	Ca_05919.1|Ca_LG_5:8263723-8268595|plus|	gi|595836327|ref|XP_007207234.1|	Asparagine synthetase [glutamine-hydrolyzing]
**Car-miR-172.1**	Ca_05270.1|Ca_LG_5:546140-552687|minus|	gi|502131795|ref|XP_004501104.1|	Chromo domain protein LHP1
**Car-miR-172.1**	Ca_07755.1|Ca_LG_7:5861320-5868245|minus|	gi|502153776|ref|XP_004509458.1|	Serine/threonine-protein kinase/endoribonuclease IRE1
**Car-miR-172.1**	Ca_18474.2|scaffold03598:8728-17057|minus|	gi|502170998|ref|XP_004514963.1|	Transcription factor GTE8
**Car-miR-172.1**	Ca_18528.1|scaffold03674:4231-6546|plus|	gi|502121962|ref|XP_004497511.1|	BTB/POZ domain-containing protein At5g03250
**Car-miR-172.1**	Ca_07956.1|Ca_LG_7:8405701-8414932|plus|	gi|502138662|ref|XP_004503487.1|	Nucleotide-diphospho-sugar transferases superfamily protein
**Car-miR-172.2**	Ca_00883.1|Ca_LG_1:11416534-11420767|minus|	gi|502082378|ref|XP_004487148.1|	Ethylene-responsive transcription factor RAP2-7
**Car-miR-172.2**	Ca_20442.1|scaffold12301:3162-3527|plus|	gi|357496583|ref|XP_003618580.1|	F-box/FBD/LRR-repeat protein
**Car-miR-172.2**	Ca_07755.1|Ca_LG_7:5861320-5868245|minus|	gi|502153776|ref|XP_004509458.1|	Serine/threonine-protein kinase/endoribonuclease IRE1
**Car-miR-172.3**	Ca_00883.1|Ca_LG_1:11416534-11420767|minus|	gi|502082378|ref|XP_004487148.1|	Ethylene-responsive transcription factor RAP2-7
**Car-miR-172.4**	Ca_00883.1|Ca_LG_1:11416534-11420767|minus|	gi|502082378|ref|XP_004487148.1|	Ethylene-responsive transcription factor RAP2-7
**Car-miR-390.1**	Ca_00574.1|Ca_LG_1:7187400-7209438|minus|	gi|502085903|ref|XP_004488044.1|	Receptor-like serine/threonine-protein kinase ALE2
**Car-miR-390.1**	Ca_11159.1|scaffold00166:189795-193329|minus|	gi|828293958|ref|XP_004489835.2|	Proline-rich receptor-like protein kinase PERK12
**Car-miR-390.1**	Ca_17496.1|scaffold02476:20715-23935|plus|	gi|502097596|ref|XP_004491031.1|	Zinc finger CCCH domain-containing protein 29
**Car-miR-390.1**	Ca_12680.1|scaffold00449:42095-45597|plus|	gi|502106329|ref|XP_004492917.1|	Probable LRR receptor-like serine/threonine-protein kinase At4g08850
**Car-miR-396.1**	Ca_08684.1|Ca_LG_8:9854372-9857630|plus|	gi|356563584|ref|XP_003550041.1|	Cysteine proteinase RD21a
**Car-miR-396.1**	Ca_01823.1|Ca_LG_2:12553150-12557687|minus|	gi|502148203|ref|XP_004507075.1|	NDH-dependent cyclic electron flow 1
**Car-miR-396.1**	Ca_09562.1|scaffold00659:51482-60019|minus|	gi|502156075|ref|XP_004510304.1|	Insulin-degrading enzyme
**Car-miR-396.1**	Ca_15231.1|scaffold01214:31529-43492|minus|	gi|502165064|ref|XP_004513398.1|	Eukaryotic translation initiation factor 2D
**Car-miR-396.1**	Ca_17914.1|scaffold02845:348-4059|minus|	gi|502080020|ref|XP_004486436.1|	Uncharacterized protein At4g37920, chloroplastic
**Car-miR-396.1**	Ca_10849.1|scaffold00105:137552-144973|minus|	gi|828331422|ref|XP_012574582.1|	MORC family CW-type zinc finger protein 3
**Car-miR-396.2**	Ca_05170.1|Ca_LG_4:21207284-21239561|plus|	gi|502117300|ref|XP_004495773.1|	WD repeat-containing protein 48
**Car-miR-396.2**	Ca_02392.1|Ca_LG_3:2984967-2994947|plus|	gi|502113482|ref|XP_004494665.1|	WD repeat-containing protein 48
**Car-miR-396.2**	Ca_11811.1|scaffold00286:19396-26360|minus|	gi|502085111|ref|XP_004487839.1|	Protein pleiotropic regulatory locus 1
**Car-miR-397**	Ca_10259.1|scaffold01445:34515-40115|plus|	gi|502146484|ref|XP_004506473.1|	Uncharacterized aarF domain-containing protein kinase At1g79600
**Car-miR-397**	Ca_05118.1|Ca_LG_4:20676173-20679438|plus|	gi|502116752|ref|XP_004495578.1|	RNA polymerase III RPC4
**Car-miR-397**	Ca_14405.1|scaffold00897:12512-15603|plus|	gi|502080260|ref|XP_004486508.1|	Probable WRKY transcription factor 14
**Car-miR-397**	Ca_01308.1|Ca_LG_2:3750909-3753820|plus|	gi|502097255|ref|XP_004490949.1|	Laccase-4
**Car-miR-397**	Ca_00561.1|Ca_LG_1:6856707-6865098|plus|	gi|502085707|ref|XP_004487981.1|	Subtilisin-like protease
**Car-miR-398.1**	Ca_03317.1|Ca_LG_3:16957384-16958820|minus|	gi|502160760|ref|XP_004511884.1|	UDP-glycosyltransferase 84B2
**Car-miR-398.1**	Ca_18515.1|scaffold03650:8908-10601|plus|	gi|502093565|ref|XP_004489982.1|	Nitrate transporter 1.7
**Car-miR-398.2**	Ca_02233.1|Ca_LG_3:918792-920042|plus|	gi|502112976|ref|XP_004494503.1|	Transcription factor MYB86
**Car-miR-398.2**	Ca_12045.1|scaffold00325:53439-55646|minus|	gi|502170167|ref|XP_004514773.1|	Probable pectate lyase 3
**Car-miR-398.3**	Ca_02409.2|Ca_LG_3:3241167-3244110|minus|	gi|830260275|ref|NP_001296637.1|	Superoxide dismutase [Cu-Zn]
**Car-miR-399.1**	Ca_00238.1|Ca_LG_1:2316790-2321569|plus|	gi|502077908|ref|XP_004485781.1|	Probable ubiquitin-conjugating enzyme E2 24
**Car-miR-399.1**	Ca_02119.1|Ca_LG_2:16684759-16692064|minus|	gi|828296786|ref|XP_012568708.1|	Cc-nbs-lrr resistance protein
**Car-miR-399.2**	Ca_00238.1|Ca_LG_1:2316790-2321569|plus|	gi|502077908|ref|XP_004485781.1|	Probable ubiquitin-conjugating enzyme E2 24
**Car-miR-399.2**	Ca_00238.4|Ca_LG_1:2317597-2322634|plus|	gi|502077908|ref|XP_004485781.1|	Probable ubiquitin-conjugating enzyme E2 24
**Car-miR-399.3**	Ca_02119.1|Ca_LG_2:16684759-16692064|minus|	gi|828296786|ref|XP_012568708.1|	Cc-nbs-lrr resistance protein
**Car-miR-399.5**	Ca_00238.2|Ca_LG_1:2316790-2322634|plus|	gi|502077908|ref|XP_004485781.1|	Probable ubiquitin-conjugating enzyme E2 24
**Car-miR-399.5**	Ca_19643.1|scaffold06355:2371-8929|minus|	gi|502087239|ref|XP_004488472.1|	Cleavage stimulation factor subunit 1

**Car-miR-399.5**	Ca_03401.1|Ca_LG_3:17894075-17900552|plus|	gi|502160053|ref|XP_004511617.1|	Protein FAM63A
**Car-miR-399.8**	Ca_00238.2|Ca_LG_1:2316790-2322634|plus|	gi|502077908|ref|XP_004485781.1|	Probable ubiquitin-conjugating enzyme E2 24
**Car-miR-530.1**	Ca_20706.2|scaffold17680:3-1368|minus|	gi|502159118|ref|XP_004511399.1|	Putative uncharacterized protein
**Car-miR-530.1**	Ca_16614.1|scaffold01893:21059-23922|minus|	gi|502148657|ref|XP_004507218.1|	Putative uncharacterized protein
**Car-miR-530.1**	Ca_04743.1|Ca_LG_4:12128568-12151302|minus|	gi|502118370|ref|XP_004496233.1|	Protein MOR1
**Car-miR-530.1**	Ca_12557.1|scaffold00418:105824-109844|plus|	gi|828294584|ref|XP_012568337.1|	Ninja-family protein AFP2
**Car-miR-598.1**	Ca_17196.2|scaffold02271:7481-12008|plus|	gi|828310888|ref|XP_004499932.2|	CTL-like protein DDB_G0274487
**Car-miR-598.1**	Ca_16440.1|scaffold01792:10321-11925|minus|	gi|502165759|ref|XP_004513681.1|	MATE efflux family protein 9
**Car-miR-598.1**	Ca_17814.1|scaffold02739:34561-36590|plus|	gi|502137582|ref|XP_004503131.1|	Putative F-box/LRR-repeat protein 23
**Car-miR-598.1**	Ca_20569.1|scaffold14311:1113-2683|minus|	gi|502147382|ref|XP_004506762.1|	Leucine-rich repeat receptor protein kinase EXS
**Car-miR-598.1**	Ca_05519.1|Ca_LG_5:4014584-4015618|plus|	-	--
**Car-miR-598.1**	Ca_19463.1|scaffold05745:8360-8662|plus|	gi|828291619|ref|XP_012575251.1|	Putative uncharacterized protein
**Car-miR-598.1**	Ca_13997.1|scaffold00776:77955-93842|plus|	gi|502170818|ref|XP_004514922.1|	Vacuolar protein sorting-associated protein 8 homolog
**Car-miR-598.1**	Ca_07098.1|Ca_LG_6:8134029-8149660|minus|	gi|502141718|ref|XP_004504604.1|	IAA-alanine resistance protein 1
**Car-miR-598.1**	Ca_04687.1|Ca_LG_4:11478791-11480077|minus|	gi|502120015|ref|XP_004496819.1|	F-box/kelch-repeat protein At3g23880
**Car-miR-598.1**	Ca_03966.1|Ca_LG_4:1134510-1135665|minus|	gi|502125872|ref|XP_004499096.1|	Protein of unknown function (DUF1645)
**Car-miR-598.1**	Ca_07162.1|Ca_LG_6:9264743-9268103|plus|	gi|502141882|ref|XP_004504685.1|	Uncharacterized urease accessory protein ureG-like
**Car-miR-598.1**	Ca_14169.1|scaffold00816:29419-31368|minus|	gi|502098440|ref|XP_004491234.1|	RPM1-interacting protein 4 (RIN4) family protein
**Car-miR-598.1**	Ca_18226.1|scaffold03217:4199-9218|minus|	gi|828308287|ref|XP_012570647.1|	Uncharacterised conserved protein (UCP030365)
**Car-miR-598.1**	Ca_13731.1|scaffold00698:68382-70165|plus|	gi|502139781|ref|XP_004503901.1|	NAC domain-containing protein 2
**Car-miR-598.1**	Ca_03107.1|Ca_LG_3:13993166-13996296|plus|	gi|502167684|ref|XP_004514216.1|	Omega-3 fatty acid desaturase, chloroplastic
**Car-miR-598.1**	Ca_03786.1|Ca_LG_3:21874617-21877073|minus|	gi|828332578|ref|XP_012574790.1|	Ectonucleotide pyrophosphatase/phosphodiesterase family member 1
**Car-miR-598.1**	Ca_06619.1|Ca_LG_6:2589238-2593408|minus|	gi|502096694|ref|XP_004490812.1|	Protein of unknown function, DUF593
**Car-miR-598.1**	Ca_16084.1|scaffold01578:40444-44970|plus|	gi|502147402|ref|XP_004506769.1|	Omega-3 fatty acid desaturase, chloroplastic
**Car-miR-598.1**	Ca_06776.1|Ca_LG_6:4279010-4279653|plus|	gi|694421704|ref|XP_009338686.1|	--
**Car-miR-598.1**	Ca_06110.1|Ca_LG_5:10550324-10551949|plus|	gi|828314739|ref|XP_012571815.1|	--
**Car-miR-598.2**	Ca_15671.1|scaffold01387:21186-23218|plus|	gi|502126920|ref|XP_004499492.1|	Zinc finger protein ZAT4
**Car-miR-598.2**	Ca_10558.1|scaffold03293:16481-17257|plus|	gi|502155435|ref|XP_004510071.1|	UPF0496 protein 3
**Car-miR-598.2**	Ca_16440.1|scaffold01792:10321-11925|minus|	gi|502165759|ref|XP_004513681.1|	MATE efflux family protein 9
**Car-miR-598.2**	Ca_17196.2|scaffold02271:7481-12008|plus|	gi|828310888|ref|XP_004499932.2|	CTL-like protein DDB_G0274487
**Car-miR-598.2**	Ca_17652.2|scaffold02602:33859-36473|minus|	gi|502132665|ref|XP_004501470.1|	Fe(2+) transport protein 3, chloroplastic
**Car-miR-598.2**	Ca_18187.2|scaffold03171:4541-8219|minus|	gi|502145681|ref|XP_004506131.1|	Histone deacetylase HDT1
**Car-miR-598.2**	Ca_05570.1|Ca_LG_5:4754532-4757370|minus|	gi|502132592|ref|XP_004501436.1|	Mechanosensitive ion channel protein 10
**Car-miR-598.2**	Ca_03966.1|Ca_LG_4:1134510-1135665|minus|	gi|502125872|ref|XP_004499096.1|	Protein of unknown function (DUF1645)
**Car-miR-598.2**	Ca_07162.1|Ca_LG_6:9264743-9268103|plus|	gi|502141882|ref|XP_004504685.1|	Uncharacterized urease accessory protein ureG-like
**Car-miR-598.2**	Ca_08508.2|Ca_LG_8:6989627-6995530|plus|	gi|502156683|ref|XP_004510595.1|	Uncharacterized protein C630.12
**Car-miR-598.2**	Ca_14038.1|scaffold00785:73208-74557|minus|	gi|502128785|ref|XP_004500079.1|	Protein of unknown function, DUF584
**Car-miR-598.2**	Ca_05101.1|Ca_LG_4:20517002-20520531|plus|	gi|502116708|ref|XP_004495561.1|	Protein EIN4
**Car-miR-598.2**	Ca_03107.1|Ca_LG_3:13993166-13996296|plus|	gi|502167684|ref|XP_004514216.1|	Omega-3 fatty acid desaturase, chloroplastic
**Car-miR-598.2**	Ca_06530.1|Ca_LG_6:1414867-1415897|minus|	gi|502091950|ref|XP_004489676.1|	--
**Car-miR-598.2**	Ca_06619.1|Ca_LG_6:2589238-2593408|minus|	gi|502096694|ref|XP_004490812.1|	Protein of unknown function, DUF593

**Car-miR-598.2**	Ca_15141.1|scaffold01173:75662-79005|plus|	gi|502157927|ref|XP_004510958.1|	Protein TRANSPARENT TESTA 1
**Car-miR-598.2**	Ca_10706.1|scaffold00077:216702-219582|minus|	gi|502109456|ref|XP_004493650.1|	Isoamylase 2, chloroplastic
**Car-miR-598.2**	Ca_04076.1|Ca_LG_4:2821982-2823069|plus|	gi|502125231|ref|XP_004498853.1|	
**Car-miR-598.2**	Ca_17814.1|scaffold02739:34561-36590|plus|	gi|502137582|ref|XP_004503131.1|	Putative F-box/LRR-repeat protein 23
**Car-miR-598.2**	Ca_11558.1|scaffold00252:85857-89448|plus|	gi|502174911|ref|XP_004515628.1|	B3 domain-containing transcription factor ABI3
**Car-miR-598.2**	Ca_00981.1|Ca_LG_1:13692303-13696264|plus|	gi|502080992|ref|XP_004486732.1|	
**Car-miR-598.2**	Ca_03287.1|Ca_LG_3:16628208-16629764|minus|	gi|502160678|ref|XP_004511844.1|	Cyclin-D5-3
**Car-miR-598.2**	Ca_20999.1|scaffold34238:229-819|minus|	gi|491052415|ref|WP_004914066.1|	Biopolymer transport protein exbD1
**Car-miR-598.2**	Ca_13997.1|scaffold00776:77955-93842|plus|	gi|502170818|ref|XP_004514922.1|	Vacuolar protein sorting-associated protein 8 homolog
**Car-miR-598.2**	Ca_03622.1|Ca_LG_3:19919663-19925790|plus|	gi|502159188|ref|XP_004511423.1|	4-coumarate--CoA ligase 2
**Car-miR-598.2**	Ca_15273.1|scaffold01229:63108-65070|plus|	gi|502097066|ref|XP_004490903.1|	
**Car-miR-598.2**	Ca_04704.2|Ca_LG_4:11645496-11648531|minus|	gi|502118489|ref|XP_004496292.1|	Serine carboxypeptidase-like 27
**Car-miR-598.2**	Ca_16646.1|scaffold01910:3859-5073|plus|	gi|502138414|ref|XP_004503397.1|	F-box/kelch-repeat protein At3g06240
**Car-miR-598.2**	Ca_06937.1|Ca_LG_6:6309690-6315618|plus|	gi|502143588|ref|XP_004505393.1|	WUSCHEL-related homeobox 8
**Car-miR-598.4**	Ca_05938.3|Ca_LG_5:8507260-8517502|minus|	gi|502135787|ref|XP_004502452.1|	U-box domain-containing protein 4
**Car-miR-598.4**	Ca_13217.1|scaffold00568:54849-59013|minus|	gi|502128697|ref|XP_004500050.1|	Purple acid phosphatase 18
**Car-miR-598.4**	Ca_00709.1|Ca_LG_1:9539459-9540335|minus|	gi|828308499|ref|XP_012570681.1|	--
**Car-miR-828**	Ca_10084.1|scaffold01296:36218-44379|plus|	gi|502178381|ref|XP_004516239.1|	Agmatine deiminase
**Car-miR-828**	Ca_00680.1|Ca_LG_1:9038319-9040175|plus|	gi|502183395|ref|XP_004517128.1|	
**Car-miR-828**	Ca_08288.1|Ca_LG_8:4758234-4759101|minus|	gi|502150891|ref|XP_004508175.1|	ABA-responsive protein ABR18
**Car-miR-1122**	Ca_14461.1|scaffold00922:33930-40619|plus|	gi|502092161|ref|XP_004489726.1|	IST1-like protein
**Car-miR-1122**	Ca_15680.1|scaffold01389:64697-70937|minus|	gi|502097614|ref|XP_004491035.1|	Uncharacterized protein YKL027W
**Car-miR-1122**	Ca_12606.2|scaffold00429:73070-76995|plus|	gi|502157245|ref|XP_004510806.1|	Uncharacterized protein At3g61260
**Car-miR-1122**	Ca_02251.1|Ca_LG_3:1087455-1091087|plus|	gi|502112905|ref|XP_004494481.1|	Charged multivesicular body protein 7
**Car-miR-1122**	Ca_17997.1|scaffold02926:1010-18719|minus|	gi|502126726|ref|XP_004499416.1|	Zinc finger Ran-binding domain-containing protein 3

Supplementary Table S2- List of predicted known and novel miRNAs from high throughput smallRNA sequencing in chickpea root apex under PEG/Salt treatments.

Name	Sequence	Size	Position	Start	End	miRNA*	Reads Per Million (RPM)					Normalized RPM				
							Con	PEG 1Hr	PEG 2Hr	NaCl 1 Hr	NaCl 2 Hr	Con	PEG 1Hr	PEG 2Hr	NaCl 1 Hr	NaCl 2 Hr
Car-miR-10d	TGAGAATTCCGATTGGTTGCC	21	Ca7	11801816	11801837	-	11.9	12.7	68.0	14.1	7.1	0.2	0.2	1	0.2	0.1
Car-miR-1144a.1	GGAATTTCTTGGGCACCCAT	20	Ca2	30978295	30978315	-	11.9	60.1	31.4	42.4	56.8	0.2	1	0.5	0.7	0.9
Car-miR-1144a.2	TGGAATTCTCGGGCAATTTT	20	Scaffold1116	64810	64830	-	11.9	0.0	0.0	0.0	0.0	1	0	0	0	0
Car-miR-1185	TGAGAGGAATTCCATTGGTTC	21	Ca4	13377674	13377695	Yes	118.8	104.4	240.5	183.7	340.7	0.3	0.3	0.7	0.5	1
Car-miR-12	CACGTGCTTCCGAAAATTCCA	21	Scaffold864	115406	115427	-	11.9	63.3	31.4	42.4	63.9	0.2	1.0	0.5	0.7	1
Car-miR-1227	GCCCTTTCCTCTGCCGGGGA	20	*	0	0	Yes	0	0	0	0	14.2	0	0	0	0	1
Car-miR-1271	CGCACCCTCAAATGCACCTCT	21	Ca4	30646943	30646964	-	0	3.2	0	0	7.1	0	0.4	0	0	1
Car-miR-1273g	AGCGGTGAAATGCGTAGAGAT	21	Ca1	45003754	45003775	Yes	0	0	0	0	14.2	0	0	0	0	1
Car-miR-130	AAATCGTAAAAGTTGGTCGCC	21	Ca3	30078128	30078149	-	23.8	19.0	0	0	7.1	1	0.8	0	0	0.3
Car-miR-1304	TGTAGGCACAATGGAATATT	20	Ca2	17542111	17542131	-	6036.0	1638.3	2724.0	7219.7	3684.0	0.8	0.2	0.4	1	0.5
Car-miR-133	TCGTTTGGTGCTGTGGGCACA	21	Ca4	5338044	5338065	-	0	3.2	0	0	0	0	1	0	0	0
Car-miR-138.1	AGAATTGATTGATGTTGCTCT	21	Ca3	18932148	18932169	-	23.8	28.5	31.4	14.1	28.4	0.8	0.9	1.0	0.5	0.9
Car-miR-138.2	GATGCACCATCCATCACGAGT	21	Ca4	1298613	1298634	-	23.8	12.7	2352.8	14.1	624.7	0.0	0.0	1.0	0.0	0.3
Car-miR-140	TGTAGAATTCCACTTGGAGTC	21	Scaffold1631	30222	30243	Yes	95.1	94.9	146.4	84.8	163.3	0.6	0.6	0.9	0.5	1
Car-miR-1421o	TGCACCCAAGTGGAACTTCTC	21	Ca3	24481343	24481364	Yes	23.8	0	5.2	0	0	1	0	0.2	0	0
Car-miR-1422c	CCACCATCAACAAAGGTTTCT	21	Ca6	11236113	11236134	Yes	0	3.16	0	0	0	0	1	0	0	0
Car-miR-146	TGGACGACGAATTCCATTAGTTGC	24	Ca7	28365029	28365053	-	11.88	9.49	5.23	0	35.49	0.3	0.3	0.1	0	1
Car-miR-1484	TCGTAAAGCGTCTGTAGCCAC	21	*	0	0	Yes	3849.72	1590.87	2650.83	1653.03	3684.04	1	0.4	0.7	0.4	1.0
Car-miR-148a	GAGAGTGCCTACACAAAATTCC	22	Ca1	175275	175297	-	71.29	34.79	94.11	56.51	21.30	0.8	0.4	1.0	0.6	0.2
Car-miR-153	TAGAGCACAAATAGAAATTCTT	22	Scaffold1348_1	722251	722273	-	0	0	0	0	7.10	0	0	0	0	1

Car-miR-1532	AACTCCAAGATGAGAGGATGCTG	23	Ca6	54104417	54104440	Yes	11.88	0	0	0	0	1	0	0	0	0
Car-miR-156d	GTACCTTCTGCCTGCTGCCTGG	22	Ca7	11900532	11900554	-	0	0	0	0	21.30	0	0	0	0	1
Car-miR-15c	GAAATTCCTTCTTGGTGGTGC	21	Ca6	30565235	30565256	-	0	0	5.23	0	7.10	0	0	0.7	0	1
Car-miR-1676	GCACCAATATCAGCATCTCT	20	Ca6	16039056	16039076	Yes	142.58	262.51	10.46	56.51	433.00	0.3	0.6	0.0	0.1	1
Car-miR-171	AGAGATTGATGGAGCACCAAA	21	Ca6	58475929	58475950	-	0	15.81	20.91	14.13	0	0	0.8	1	0.7	0
Car-miR-1719	ACAAGTATGCTTGATGGTCGC	21	Ca6	17970073	17970094	-	0	186.60	0	0	0	0	1	0	0	0
Car-miR-1729	AGTATCGTCACCGCAGTAGAG	21	Scaffold731	369934	369955	Yes	0	6.33	0	0	7.10	0	0.9	0	0	1
Car-miR-172b.1	ACACACCAAATTGTTTATAT	20	Scaffold10	10635	10655	-	35.65	28.46	0	0	14.20	1	0.8	0	0	0.4
Car-miR-172b.2	CATGATAGAAAATTTGGTGC	20	Scaffold1369	155827	155847	-	213.87	82.23	0	14.13	127.77	1	0.4	0	0.1	0.6
Car-miR-172e	GCACCAATAGAGTTACTTCT	20	Ca5	23746450	23746470	Yes	0	6.33	0	0	0	0	1	0	0	0
Car-miR-1730	TGCAACAAAGTGGGAATTCT	20	Ca3	1419905	1419925	-	297.05	234.04	245.74	367.34	149.07	0.8	0.6	0.7	1	0.4
Car-miR-1868	TTCACCTACGGAAACCTTGTTACG	24	Scaffold158	118337	118361	Yes	11.88	50.60	26.14	56.51	56.79	0.2	0.9	0.5	1.0	1
Car-miR-192b	TAAGCACCAAATGGAATCCCT	21	Ca5	3470982	3471003	-	11.88	22.14	20.91	0	21.30	0.5	1	0.9	0	1.0
Car-miR-2039	ATAAAAAAATTTGGGTGCCAA	20	Ca4	10917778	10917798	Yes	0	0	5.23	0	0	0	0	1	0	0
Car-miR-2057	TCGGGAAAAGGATTGGCTCTGA	22	Scaffold2711	1594	1616	Yes	142.58	53.77	20.91	0	227.15	0.6	0.2	0.1	0	1
Car-miR-2060c	GAGCAGAACCAAGTGGAATT	20	Ca2	12734988	12735008	-	47.53	69.58	20.91	84.77	35.49	0.6	0.8	0.2	1	0.4
Car-miR-20b	AGGATGTGATGGTGCACCCAT	21	Ca2	33336729	33336750	-	0	3.16	0	0	0	0	1	0	0	0
Car-miR-210	ACTTCTGGAGCACCATCAATC	21	Ca4	9282289	9282310	Yes	1508.99	755.90	721.53	819.45	660.15	1	0.5	0.5	0.5	0.4
Car-miR-215	CTATCACCAAGTGTTTCTATC	21	Ca7	1899654	1899675	-	0	6.33	0	0	7.10	0	0.9	0	0	1
Car-miR-2151.1	GCAGCACCAAATGAGAAATTT	21	Ca6	45555139	45555160	-	35.65	9.49	10.46	14.13	35.49	1	0.3	0.3	0.4	1.0
Car-miR-2151.2	GAATGCACCTAAATGGAATCCT	22	Scaffold109_1	824518	824540	-	0	0	0	0	7.10	0	0	0	0	1
Car-miR-2277	GCGTATCGACCCGTGCAATGCT	22	gi|197294093|ref|NC_011163.1|	97983	98005	Yes	95.05	18.98	36.60	0	35.49	1	0.2	0.4	0	0.4
Car-miR-2333	TAAATACTCCTAGTTGGTGCA	21	Ca7	40842414	40842435	Yes	0	6.33	10.46	28.26	14.20	0	0.2	0.4	1	0.5

Car-miR-2344	AGATCGTTACGCCTTTCGTGC	21	Scaffold731	372620	372641	Yes	201.99	37.95	156.85	0	141.97	1	0.2	0.8	0	0.7
Car-miR-23a	TAATCATGTGCCTAGAATCCA	21	Scaffold886	96723	96744	-	0	0	0	14.13	0	0	0	0	1	0
Car-miR-2496.1	TTCTTGGTGCACCATTAAATC	21	Ca1	39729407	39729428	-	59.41	502.88	120.25	127.16	92.28	0.1	1	0.2	0.3	0.2
Car-miR-2496.2	AGATTTGATCGGGCACCAAAA	21	Ca2	4689002	4689023	-	0	22.14	5.23	0	0	0	1	0.2	0	0
Car-miR-2496.3	GGTTGGTGCACCATCAAACCAT	22	Ca3	22698895	22698917	-	23.76	145.49	94.11	70.64	127.77	0.2	1	0.6	0.5	0.9
Car-miR-252	TGATTGATGTGCCTACAGGAC	21	Ca7	41677697	41677718	Yes	1996.15	1666.78	1866.56	1313.95	1220.91	1.0	0.8	0.9	0.7	0.6
Car-miR-2520	ATTGCCCCACCTGACCCTGAAGTT	24	Ca4	9758573	9758597	Yes	1057.48	1040.55	1777.68	762.94	645.95	0.6	0.6	1	0.4	0.4
Car-miR-252b	TGATGTGCCTGTTCAGTGATT	21	Ca6	50605390	50605411	Yes	35.65	0	0	0	0	1	0	0	0	0
Car-miR-2570	TAGTGGAAATGCTCGGGCGAC	21	Ca1	25941424	25941445	-	0	9.49	15.69	14.13	14.20	0	0.6	1	0.9	0.9
Car-miR-2575	ATGAGTGAACCACATGTGCTTA	22	Ca5	31794789	31794811	Yes	0	12.65	0	0.00	0	0	1	0	0	0
Car-miR-2584	TGTTTGTAGGGAGCATCAAATC	22	Scaffold271	125912	125934	-	23.76	28.46	47.06	14.13	28.39	0.5	0.6	1	0.3	0.6
Car-miR-2592bp	CTAGTAACTTCCTCTTGTCGC	21	Scaffold3500	74113	74134	-	71.29	50.60	52.28	0	42.59	1	0.7	0.7	0	0.6
Car-miR-2661	TGCATCAATTTTCATCTGTTT	21	Ca1	4932190	4932211	-	6035.98	1613.01	1103.20	2133.40	3712.43	1	0.3	0.2	0.4	0.6
Car-miR-2672	AAGAACGACCAAGGTGGAATTA	22	Ca5	15668262	15668284	-	0	9.49	94.11	14.13	0	0	0.1	1	0.2	0
Car-miR-2690	TTGGAAAGGATTGGCTCTGAGGG	23	*	0	0	Yes	0	0	0	0	7.10	0	0	0	0	1
Car-miR-26a	CCGAAGAACTTCCATTGTTGCC	22	Scaffold1369	64640	64662	-	0	6.33	5.23	14.13	7.10	0	0.4	0.4	1	0.5
Car-miR-277	GCCGCACCACCTCCAGTTCAT	21	*	0	0	-	0	0	0	0	7.10	0	0	0	0	1
Car-miR-2838	AGAATTCCTACTTTAGGTGCT	21	Scaffold895	14043	14064	-	11.88	6.33	10.46	0	0	1	0.5	0.9	0	0
Car-miR-2846	ACCTACAGAAACCTTGTTACG	21	Scaffold227	143916	143937	Yes	1687.22	1584.55	2624.69	353.21	1533.24	0.6	0.6	1	0.1	0.6
Car-miR-2878	GTAACAAGGTTTTTTGTTGTTTGA	24	Ca4	32830757	32830781	-	59.41	9.49	94.11	0	28.39	0.6	0.1	1	0	0.3
Car-miR-29	TGCACCAAAGGAATTCTATACT	22	Ca5	9919857	9919879	-	0	3.16	0	0	0	0	1	0	0	0
Car-miR-2948	TAAGAATTGATGGTGGCACCAA	22	Scaffold421_2	14125	14147	Yes	0	91.72	57.51	56.51	21.30	0	1	0.6	0.6	0.2
Car-miR-3079	TTAGTGCCTACAGAGTATGCA	21	Ca7	40707859	40707880	Yes	0	9.49	0	0	0	0	1	0	0	0
Car-miR-309a	ATAATTCCGACTTGGTGCG	19	Ca4	10250571	10250590	-	59.41	66.42	135.94	70.64	92.28	0.4	0.5	1	0.5	0.7

Car-miR-3109	GATTTATGGTGGCACCAAAGA	21	Scaffold674	115825	115846	-	0	91.72	57.51	56.51	21.30	0	1	0.6	0.6	0.2
Car-miR-319b	AATCGTGATTTGAATGGTGC	20	Ca3	19044898	19044918	-	0	0	5.23	0	0	0	0	1	0	0
Car-miR-3334	AATTCCTACTTTAGGTGCTTTAA	23	Ca2	23091730	23091753	-	11.88	6.33	10.46	0	0	1	0.5	0.9	0.0	0
Car-miR-3375.1	TAAAGCACCAAAAGGAGGAATT	22	Ca7	13388472	13388494	-	23.76	15.81	20.91	28.26	49.69	0.5	0.3	0.4	0.6	1
Car-miR-3375.2	TAGCACCAAAGTTGGAATTAA	21	Scaffold537	257545	257566	-	0	3.16	10.46	0	35.49	0	0.1	0.3	0	1
Car-miR-3437	AACACACCAAATTGTTTATAT	21	Ca7	12541153	12541174	-	35.65	28.46	0	0	14.20	1	0.8	0	0	0.4
Car-miR-345	AGCGAGTGTTGCTGGAATTCTC	22	Ca3	39546880	39546902	-	748.56	1435.90	365.99	649.91	1341.59	0.5	1	0.3	0.5	0.9
Car-miR-3478	TCAAGAATCCTACGGTTGGTGC	22	Ca8	14756498	14756520	-	0	6.33	20.91	0	0	0	0.3	1	0	0
Car-miR-361.1	TAATAGAAACCCTAGTTGGTGC	22	Ca1	7316642	7316664	-	0	3.16	0	0	0	0	1	0	0	0
Car-miR-361.2	TAAAGTAACTTCTATTGGTGC	21	Ca3	29893081	29893102	Yes	23.76	9.49	41.83	42.39	21.30	0.6	0.2	1.0	1	0.5
Car-miR-361.3	TTTAACAGAATTCCCATGTGC	21	Ca4	12597060	12597081	-	0	15.81	10.46	56.51	0	0	0.3	0.2	1	0
Car-miR-3629a	CACCACCAGTTTCATCACAAC	21	Ca6	26765678	26765699	Yes	0	9.49	0	0	0	0	1	0	0	0
Car-miR-3680	TGCACCCTAGATGGTGAAAGTC	22	Ca1	45007220	45007242	Yes	11.88	6.33	20.91	0	7.10	0.6	0.3	1	0	0.3
Car-miR-3895	TTGGAATTCTCGGCATGAT	19	Scaffold453	58850	58869	-	308.93	619.90	454.88	409.73	369.11	0.5	1	0.7	0.7	0.6
Car-miR-39	GGGTTCGAGTGAGAGCATACC	21	Scaffold2888	378	399	Yes	83.17	278.32	0	42.39	63.89	0.3	1	0	0.2	0.2
Car-miR-3934	TGAGGAAATTTCAGTAGGCAC	21	Ca3	9000852	9000873	-	106.94	88.56	125.48	141.28	312.33	0.3	0.3	0.4	0.5	1
Car-miR-395a	TTTGAAGGTGGTGGTGGTGAA	21	Ca3	3275152	3275173	Yes	0	0	0.00	0	14.20	0	0	0	0	1
Car-miR-3982	CCTGTGCCTCCAGGGGCACCT	21	Ca4	16982677	16982698	-	190.11	142.32	31.37	70.64	170.36	1	0.7	0.2	0.4	0.9
Car-miR-398c	GGATGACCTTGAAGTTCATCC	21	Scaffold352	145052	145073	-	11.88	37.95	10.46	42.39	14.20	0.3	0.9	0.2	1	0.3
Car-miR-399i.1	GTGTGCACATGTGTAGGCACA	21	Ca3	31455775	31455796	-	0	6.33	5.23	14.13	7.10	0	0.4	0.4	1	0.5
Car-miR-399i.2	CTGGCGACCAAATAGGAAGTC	21	Ca5	28283066	28283087	Yes	23.76	18.98	78.43	14.13	21.30	0.3	0.2	1	0.2	0.3
Car-miR-4001c	AGAATTCCCACTTTGTAGCA	20	Ca6	55704638	55704658	-	0	6.33	10.46	0	21.30	0	0.3	0.5	0	1
Car-miR-422a	ATCGTGACTTTGATGGTGGTG	21	Ca4	38343522	38343543	-	0	0	10.46	0	0	0	0	1	0	0
Car-miR-	AGAATCCCACCTTAGTGCAGT	21	Ca1	299621	299621	-	11.8	18.98	26.14	42.39	28.39	0.3	0.4	0.6	1	0.7

430b				47	68		8								
Car-miR-4412	GATTGATCGTGCCTACAAGAT	21	Scaffold1193	161858	161879	-	499.04	1084.83	1354.17	1045.51	887.29	0.4	0.8	1	0.8	0.7
Car-miR-4423.1	TCCACCATCAAAGTCATACTA	21	Ca1	11236050	11236071	Yes	0	3.16	0	0	0	0	1	0	0	0
Car-miR-4423.2	TTGGCACCAATATCAGCATCT	21	Ca6	15990593	15990614	Yes	142.58	262.51	10.46	56.51	433.00	0.3	0.6	0.0	0.1	1
Car-miR-4423.3	TTGCACCAATAGCAGCATTTG	21	Scaffold5700	10313	10334	-	0	0	0	0	14.20	0	0	0	0	1
Car-miR-4512	CAGGGGCCCACTGTAGGCACCT	22	Ca1	41776591	41776613	Yes	47.53	47.44	5.23	28.26	35.49	1	1.0	0.1	0.6	0.7
Car-miR-4547	TAGGCCGCGACAATATGAAGTTCT	24	Scaffold2415	6343	6367	-	0	3.16	0	14.13	0	0	0.2	0	1	0
Car-miR-4563	TCAAGTACACCATCAATAGTCGAC	24	Ca7	44534810	44534834	-	0	0	0	0	78.08	0	0	0	0	1
Car-miR-4583	GCTACCAATAGAATTCCTTTGTA	23	Scaffold98	417003	417026	-	0	18.98	26.14	0	49.69	0	0.4	0.5	0	1
Car-miR-4591	TTACGGCGACCATGGAAGTACTT	23	Ca3	15551782	15551805	Yes	11.88	3.16	0	0	7.10	1	0.3	0	0	0.6
Car-miR-4634	CCCCGTTGTGCTCGGACCCT	20	Scaffold1776	737	757	Yes	35.65	82.23	20.91	28.26	255.54	0.1	0.3	0.1	0.1	1
Car-miR-4661	TGTACCTACAGAGATTTCCAT	21	Ca4	14308002	14308023	-	47.53	41.12	83.66	56.51	92.28	0.5	0.4	0.9	0.6	1
Car-miR-4818d.1	AATTCCAAGCTTTGGTGCAAG	21	Scaffold866	61529	61550	-	11.88	3.16	5.23	0	7.10	1	0.3	0.4	0	0.6
Car-miR-4818d.2	AGAATTTCCACTTTGGTGCAT	21	Scaffold1450	30052	30073	Yes	35.65	12.65	0	0	21.30	1	0.4	0	0	0.6
Car-miR-482a	TTCTCATCCCTTCCATTTTCAT	22	Ca2	1690154	1690176	-	0	0	0	0	7.10	0	0	0	0	1
Car-miR-4836	AGATATTGGTGCCCGAGAAT	20	Ca3	30222166	30222186	Yes	5940.92	1356.83	162.08	353.21	1944.95	1	0.2	0.0	0.1	0.3
Car-miR-4864	ATTTAGACACTTCCATAGGTGC	22	Scaffold208	365696	365718	-	0	3.16	0	0	0	0	1	0	0	0
Car-miR-4865	TGATAGAAAAATTTGGTTGCC	21	Ca3	789875	789896	-	11.88	6.33	0	0	0	1	0.5	0	0	0
Car-miR-4882b	TTGAACCAAATGGGAATTTCT	21	Scaffold1278	128805	128826	-	0	6.33	0	0	28.39	0	0.2	0	0	1
Car-miR-495	ATTTATAACTATTTTTCGTGC	21	Scaffold214	68119	68140	-	0	3.16	0	0	0	0	1	0	0	0
Car-miR-4989	TGAACCAATTTTTCATCTGTA	21	Ca2	27844544	27844565	Yes	154.46	145.49	20.91	0	312.33	0.5	0.5	0.1	0	1
Car-miR-502b	TTAATTCCACCTTAGTGACAC	21	Ca1	42596850	42596871	-	11.88	0	0	0	0	1	0	0	0	0
Car-miR-5032	AGTAGTTCCACTTGTGTCTTTTG	23	Ca5	478059	478082	-	0	0	0	0	7.10	0	0	0	0	1
Car-miR-5033	GGGTGACCAAATGGAAGTTCT	21	Ca3	24804221	24804242	-	0	6.33	83.66	28.26	28.39	0	0.1	1	0.3	0.3

Car-miR-5198	TTAGTATGATTTATGGTGTTT	21	Ca1	5532680	5532701	-	0	3.16	0	0	0	0	1	0	0	0
Car-miR-5225	AGGTATGCTCTCACTCGAACCC	22	Scaffold287	2409	2431	Yes	83.17	278.32	0	42.39	63.89	0.3	1	0	0.2	0.2
Car-miR-5258	TGAAGCGTCAATGGAAGTTACT	22	Ca5	39035565	39035587	-	0	9.49	0	0	0	0	1	0	0	0
Car-miR-5349	AGGGCTATCACCTTCTTTGGC	21	Ca6	21479605	21479626	Yes	118.82	205.58	26.14	14.13	1029.26	0.1	0.2	0.0	0.0	1
Car-miR-5352	TACAGCTACATGAGTACCATCCGG	24	Ca5	24288246	24288270	Yes	0	0	0	0	21.30	0	0	0	0	1
Car-miR-5466	TTGATTGATGGTGCCAAGGTGT	22	Ca6	48328782	48328804	Yes	0	6.33	5.23	0	0	0	1	0.8	0	0
Car-miR-5473	TAATTTGATGAATCTTGGTGC	21	Scaffold314	609255	609276	-	0	0	0	14.13	0	0	0	0	1	0
Car-miR-549a	TTAACATTGCACCATAGTGCA	21	C11137854	2378	2399	-	0	3.16	0	14.13	0	0	0.2	0	1	0
Car-miR-5507	AGATAGATGACCTTTTCGTCC	21	Ca1	36739136	36739157	Yes	0	6.33	0	0	14.20	0	0.4	0	0	1
Car-miR-550a	TGCACCACCATGGAATTTTAG	21	Scaffold575	90806	90827	-	190.11	863.43	690.16	536.88	482.69	0.2	1	0.8	0.6	0.6
Car-miR-58b	TTGTAGGCATCAAGTGGATCT	21	Ca4	41756004	41756025	-	0	6.33	5.23	56.51	35.49	0	0.1	0.1	1	0.6
Car-miR-5939	TTGAGGTGCACCAAGTGGAAT	21	Scaffold1128	129370	129391	Yes	83.17	129.67	83.66	84.77	92.28	0.6	1	0.6	0.7	0.7
Car-miR-5988.1	TAAAAGAATTCCTACTTGTTGC	22	Ca5	33399702	33399724	Yes	0	0	0	14.13	0	0	0	0	1	0
Car-miR-5988.2	GATTGATGGTGGAATTGTGTTTCA	24	Scaffold134	265625	265649	-	23.76	12.65	20.91	14.13	7.10	1	0.5	0.9	0.6	0.3
Car-miR-6090	AGTTCTGATGGGATCTGGTGC	21	Scaffold1722	50188	50209	Yes	0	0	5.23	0	21.30	0	0	0.2	0	1
Car-miR-615	TGCCAGAGAACTCTGCAGGCAC	22	C11129662	2416	2438	-	0	0	0	0	7.10	0	0	0	0	1
Car-miR-6222	TGTGACCAAACTTTACTATTG	21	Ca4	23993555	23993576	-	11.88	0	0	0	0	1	0	0	0	0
Car-miR-6335	TATGCAACAGATGGAATTCGT	21	Scaffold290	580672	580693	-	0	0	0	0	7.10	0	0	0	0	1
Car-miR-6500	TTTGGACCACCTCAGTAGTAT	21	Ca4	25660555	25660576	-	0	0	0	0	7.10	0	0	0	0	1
Car-miR-652	TGTGAATGATGGTGTGCCTACA	22	Ca2	718254	718276	-	47.53	60.09	10.46	70.64	85.18	0.6	0.7	0.1	0.8	1
Car-miR-6539	TATAAGAGAATTCCCAGGTGC	21	Ca2	34610609	34610630	-	0	41.12	26.14	56.51	63.89	0	0.6	0.4	0.9	1
Car-miR-6550	GCAGGCTCCACTCCTGGTGGTG	22	Scaffold158	118947	118969	Yes	23.76	28.46	0	0	85.18	0.3	0.3	0	0	1
Car-miR-6562.1	TTAAAGGAAAAGGAATTGGCT	21	Ca5	18804663	18804684	-	0	3.16	0	0	0	0	1	0	0	0
Car-miR-	TCTAGGTGGAAAGGATTGGCT	21	Scaffold642	547030	547051	Yes	47.5	9.49	0	0	28.39	1	0.2	0	0	0.6

6562.2							3									
Car-miR-6612.1	GATTTGATGCTGCACCATTAT	21	Ca6	3742411	3742432	-	0	34.79	109.80	56.51	85.18	0	0.3	1	0.5	0.8
Car-miR-6612.2	TTAGGTGCAACATCAATCTAA	21	Ca6	32429885	32429906	-	0	6.33	5.23	0	0	0	1	0.8	0	0
Car-miR-6718	TCTATGGTGCATTGATGGTGT	21	Ca1	6962837	6962858	Yes	1354.53	730.60	575.13	706.42	298.13	1	0.5	0.4	0.5	0.2
Car-miR-6722	AAGCATCCTGAAGTTACTAT	20	Scaffold143	190588	190608	-	0	3.16	0	14.1284844	0	0	0.2	0	1	0
Car-miR-675	GGGATTGATGCTGCCAAGAGT	21	Ca7	47974672	47974693	-	0	15.81	0	0	0	0	1	0	0	0
Car-miR-6830	AGGCCTACAAAATTCCATTGG	21	Ca1	37845260	37845281	Yes	6535.02	1767.99	3053.42	7841.31	4024.76	0.8	0.2	0.4	1	0.5
Car-miR-6930	AGGTTGGTGAGATTGATGGTGC	22	Ca6	20876687	20876709	-	5988.45	1600.36	2666.51	6301.30	3613.06	1.0	0.3	0.4	1	0.6
Car-miR-6988	AACAGATGCTGTAGTTGTTGC	21	Ca8	2450443	2450464	-	0	3.16	0	0	7.10	0	0.4	0	0	1
Car-miR-7031	GGCGGTGGCGGTGGTGGTGGA	21	Ca6	58273025	58273046	Yes	154.46	1233.48	941.12	84.77	205.85	0.1	1.0	0.8	0.1	0.2
Car-miR-7035	ATCTTGTAGGCACGATCAATC	21	Ca3	9014718	9014739	-	558.45	1277.76	1474.43	1102.02	986.67	0.4	0.9	1	0.7	0.7
Car-miR-7037.1	TTAGTGGCTACAGTTGTGCAC	21	Ca7	26733761	26733782	-	23.76	18.98	5.23	0	7.10	1	0.8	0.2	0	0.3
Car-miR-7037.2	TTTGCCTACAGGATTGGCACG	21	Scaffold1348_1	570687	570708	Yes	0	0	0	0	21.30	0	0	0	0	1
Car-miR-711	TGCATCCTCATTGGTGAGATTG	22	Ca3	36083599	36083621	Yes	0	0	0	0	7.10	0	0	0	0	1
Car-miR-7154	TAAAGGAAGTGACAATTTGAG	21	Ca1	42585274	42585295	-	0	6.33	0	0	0	0	1	0	0	0
Car-miR-718	GCCGACGCCAGGAGCGCGCAA	21	Scaffold5185	685	706	Yes	0	3.16	0	0	28.39	0	0.1	0	0	1
Car-miR-7188	TATCCGGGCTCATTGATGGTGC	22	Ca1	5738653	5738675	-	4372.52	1590.87	2154.12	2981.11	3620.15	1	0.4	0.5	0.7	0.8
Car-miR-7217	AAGTAACTCCAACTGGTGCCT	21	Ca8	7079122	7079143	-	0	9.49	67.97	14.13	0	0	0.1	1	0.2	0
Car-miR-7241.1	AAAGAATTGCATTTGGTGCCC	21	Ca4	17870322	17870343	-	4776.50	1638.31	2724.03	3786.43	3684.04	1	0.3	0.6	0.8	0.8
Car-miR-7241.2	TGCACCAGCACCTGGAATTGA	21	Ca5	38646964	38646985	Yes	11.88	31.63	26.14	42.39	49.69	0.2	0.6	0.5	0.9	1
Car-miR-7241.3	TAAAATTCCATGGTGGTGCAG	21	Scaffold336	618114	618135	-	190.11	863.43	690.16	536.88	482.69	0.2	1	0.8	0.6	0.6
Car-miR-7263	GGCACCCAAATTTTCAATTTA	21	Ca3	31326534	31326555	-	11.88	15.81	0	0	0	0.8	1.0	0	0	0
Car-miR-727.1	TCAAGCCATTCCTTTTCCCCG	21	Ca2	25882840	25882861	-	11.88	0	0	0	0	1	0	0	0	0
Car-miR-727.2	TGGAAGCCAATTCCTCTTCCCT	22	Ca6	37076814	37076836	-	0	3.16	0	0	0	0	1	0	0	0

Car-miR-7276	CAGCTCCTCCCACTGCTTGGG	21	Ca3	5659563	5659584	Yes	0	0	10.46	14.13	14.20	0	0	0.7	1.0	1
Car-miR-7330	TGGGCCTACAGTCTATCATATGTC	24	Ca6	59341873	59341897	Yes	11.88	3.16	0	0	78.08	0.2	0.0	0	0	1
Car-miR-7362	TTGATGGTGCCAACATTGAG	20	Ca8	3797202	3797222	Yes	0	3.16	0	0	0	0	1	0	0	0
Car-miR-7433	TGAGTATGATTGACTCGTGCA	21	Ca7	41973025	41973046	-	0	15.81	0	0	0	0	1	0	0	0
Car-miR-7492o	CTACAGGATGCTTATATTTGGTGT	24	Scaffold553	266327	266351	Yes	0	9.49	0	0	0	0	1	0	0	0
Car-miR-7587	TAAATAGTAACTTCGGAGGTCGC	23	*	0	0	-	475.27	211.91	146.40	183.67	163.26	1	0.4	0.3	0.4	0.3
Car-miR-763.1	GCACCAAGGGAAATTCCTCTA	21	Ca3	15369426	15369447	-	11.88	12.65	20.91	28.26	14.20	0.4	0.4	0.7	1	0.5
Car-miR-763.2	TGAAGGATTTCCATTTGGTGC	21	Ca5	45557548	45557569	-	23.76	34.79	26.14	14.13	198.75	0.1	0.2	0.1	0.1	1
Car-miR-7635	TATGTACCAAGCTGGAATTCT	21	Ca8	2646691	2646712	-	47.53	300.46	20.91	0	7.10	0.2	1	0.1	0	0.0
Car-miR-7647	AACCTTACCAACCCTTGGTGC	21	Scaffold314	406145	406166	-	0	0	5.23	0	0	0	0	1	0	0
Car-miR-7687.1	AATTGGAATTCTCTGCAGTGC	21	Scaffold451	293582	293603	-	59.41	18.98	0.00	42.39	14.20	1	0.3	0	0.7	0.2
Car-miR-7687.2	ATTTCTAAGTAACTTCGAGGTAGC	24	Scaffold1976	21268	21292	-	0	0	0	28.26	0	0	0	0	1	0
Car-miR-7734	TAATTCCATTGCAGCAATCGA	21	Ca3	34603990	34604011	-	0	0	5.23	0	0	0	0	1	0	0
Car-miR-7883b.1	TAGCACCTGGAATTCTCATAA	21	Ca2	8556324	8556345	-	1663.46	1616.17	2671.74	7134.88	3641.45	0.2	0.2	0.4	1	0.5
Car-miR-7883b.2	CTACAATAATTCCAGGTGCACA	22	Scaffold913	414694	414716	-	59.41	120.19	282.34	141.28	177.46	0.2	0.4	1	0.5	0.6
Car-miR-7907b.1	TCATGAACTTCAAGGTCAGG	20	Ca3	24452305	24452325	-	1152.54	1284.08	1965.90	918.35	809.21	0.6	0.7	1	0.5	0.4
Car-miR-7907b.2	AAAATTCCATCGACTCGTGCA	21	Ca4	7001547	7001568	-	0	18.98	0	0	0	0	1	0	0	0
Car-miR-7915	TGAAAACCTTGTTACAACCTT	21	Scaffold1060	378521	378542	-	1425.82	1581.38	2629.91	339.08	1242.21	0.5	0.6	1	0.1	0.5
Car-miR-7962	TTAAAATTTCCACTTGGTGCT	21	Ca7	4106214	4106235	Yes	47.53	69.58	20.91	84.77	35.49	0.6	0.8	0.2	1	0.4
Car-miR-8013	AGCTCATGATAGAAAATTTGGTGC	24	Scaffold1369	141428	141452	-	213.87	82.23	0	14.13	127.77	1	0.4	0	0.1	0.6
Car-miR-8107	ACAACTGGTGCTGGTGATTCT	21	Ca4	3700770	3700791	-	0	506.04	0	0	0	0	1	0	0	0
Car-miR-821d	AAACCTTTGTTGATGGTGGAGA	22	Scaffold548	483516	483538	Yes	0	3.16	0	0	0	0	1	0	0	0
Car-miR-825	TGGACAAGCCTGAAGTTCATTT	22	Ca6	34180552	34180574	-	0	3.16	0	0	21.30	0	0.1	0	0	1
Car-miR-	CCTACGGAAACCTTGTTACGA	21	Ca5	235634	235634	Yes	11.8	50.60	26.14	56.51	56.79	0.2	0.9	0.5	1.0	1

8274				57	78		8									
Car-miR-8301	ACAAAGAAATTCTATTGGTGC	21	Scaffold1281	357837	357858	-	0	6.33	5.23	0	14.20	0	0.4	0.4	0	1
Car-miR-8322	TATAAGTAAAAGTTGGTTGC	20	Scaffold1006	280260	280280	-	11.88	3.16	5.23	0	0	1	0.3	0.4	0	0
Car-miR-8447	TGGATTGATGGTGGAATTGTG	21	Scaffold296	296317	296338	-	23.76	12.65	20.91	14.13	7.10	1	0.5	0.9	0.6	0.3
Car-miR-8465	TTGCACCAATAGCAGCATTT	20	Ca7	28096526	28096546	-	0	0	0	0	14.20	0	0	0	0	1
Car-miR-8492	AACTGCTGTTGGTGTTGCTCC	21	Ca5	36069485	36069506	Yes	0	3.16	0	0	0	0	1	0	0	0
Car-miR-85	TATGACATTAATGGATTTAACT	22	Ca6	55716298	55716320	Yes	11.88	0	0	0	0	1	0	0	0	0
Car-miR-8623b	CTCCAAGGAATTCCCATTGCTAGC	24	Ca7	29473668	29473692	-	23.76	28.46	15.69	42.39	28.39	0.6	0.7	0.4	1	0.7
Car-miR-871	AAAGGCACCAAATGAAATTCT	21	Ca4	27678996	27679017	Yes	4776.50	1638.31	2724.03	3786.43	3684.04	1	0.3	0.6	0.8	0.8
Car-miR-874	CTATCGCAGCATGGAATTCTA	21	Ca4	10283110	10283131	-	5940.92	1594.03	2582.86	2020.37	2023.03	1	0.3	0.4	0.3	0.3
Car-miR-8780.1	TGAGATTGATGGTGCACCAAGAAT	24	Ca6	20890009	20890033	Yes	11.88	0	0	0	7.10	1	0	0	0	0.6
Car-miR-8780.2	TTCCCATTGATGGTGCATCAAAAT	24	Ca7	7806181	7806205	-	11.88	3.16	0	0	7.10	1	0.3	0	0	0.6
Car-miR-8824	GGGCTCTCACCCTCTCTGGCG	21	Scaffold287	33527	33548	Yes	11.88	0	10.46	0	0	1	0	0.9	0	0
Car-miR-8834a	TACAGATCGTCGCCTTGGTAGGCC	24	*	0	0	Yes	11.88	0	15.69	0	35.49	0.3	0	0.4	0	1
Car-miR-887	GTTTGGAATTTCGGGTACCAA	21	Ca6	45358141	45358162	-	23.76	9.49	0	0	7.10	1	0.4	0	0	0.3
Car-miR-888	CACGAAAGGCGTAACGATCTGGGC	24	gi|197294093|ref|NC_011163.1|	102957	102981	Yes	11.88	6.33	10.46	0	21.30	0.6	0.3	0.5	0	1
Car-miR-8885	TGGGCGTAAAGCGTCTGTAGGT	22	gi|197294093|ref|NC_011163.1|	97710	97732	Yes	3481.38	1581.38	2650.83	1483.49	3648.55	1.0	0.4	0.7	0.4	1
Car-miR-8949	TTAATTCCATGGTGTCTACG	20	Ca1	41648350	41648370	Yes	6107.27	1625.66	2823.37	7318.55	3733.73	0.8	0.2	0.4	1	0.5
Car-miR-9041	TGAATTTCCAACCTTTGGTGC	21	Scaffold332	209370	209391	-	11.88	3.16	5.23	0	7.10	1	0.3	0.4	0	0.6
Car-miR-9055.1	AGGAATTACATTGGTAGCCTTTGA	24	Ca5	31792827	31792851	-	0	18.98	26.14	0	49.69	0	0.4	0.5	0	1
Car-miR-9055.2	AGCACCAAGTGGATTCTCATA	21	Ca7	9390390	9390411	Yes	118.82	354.23	522.85	254.31	234.25	0.2	0.7	1	0.5	0.4
Car-miR-9218	ATTGGAAGAACAAGTGTTACCG	22	Ca3	32662772	32662794	Yes	0	0	0	0	14.20	0	0	0	0	1
Car-miR-9279a	TCCACCAAGGTCAAAAGGAAA	21	Ca4	25610810	25610831	-	11.88	0	0	0	0	1	0	0	0	0

Car-miR-92b	TAGGCACCAATCTTCCGTGAAT	22	Ca4	16479231	16479253	Yes	0	0	0	0	56.79	0	0	0	0	1
Car-miR-932	TCCAATTCTCGTAAGCACCAT	21	Ca6	20783672	20783693	Yes	6071.62	1666.78	2823.37	7473.97	3769.22	0.8	0.2	0.4	1	0.5
Car-miR-937	ACACCTGTACCATCAATCTTC	21	Ca1	1103277	1103298	-	0	25.30	26.14	28.26	21.30	0	0.9	0.9	1	0.8
Car-miR-938	TAAAGCACCAAGTGGAAGTATC	22	Ca4	7136519	7136541	-	47.53	3.16	10.46	0	35.49	1	0.1	0.2	0	0.7
Car-miR-967	AGATCAACTTCCCTTGGTGCA	21	Ca8	6706775	6706796	-	0	15.81	5.23	0	0	0	1	0.3	0	0
Car-miR-96a	CGGCAGCATGGAAATTTCTGT	21	Ca6	58719375	58719396	-	35.65	18.98	10.46	42.39	113.57	0.3	0.2	0.1	0.4	1
Car-miR-9788.1	AACTTGTAGGCACAATCAATC	21	Ca1	38802215	38802236	-	558.45	1277.76	1474.43	1102.02	986.67	0.4	0.9	1	0.7	0.7
Car-miR-9788.2	TTTTGGTGCCAACAAATCTAT	21	Ca1	39941752	39941773	-	0	3.16	0	0	0	0	1	0	0	0
Car-miR-995	TTTGCACCATCAAATCAGTTT	21	Ca2	29633232	29633253	-	0	0	0	0	35.49	0	0	0	0	1
Car-miR-998	TGGCATGTACCATGGGAAATTCTC	24	Ca6	53426001	53426025	-	11.88	9.49	5.23	0	21.30	0.6	0.4	0.2	0	1
Car-miR-99b	GAAGAATTCCAACTTTGTCGC	21	Ca5	16445579	16445600	-	0	0	0	0	7.10	0	0	0	0	1
Car-miR-B14RC	TCACCATCAATCTCACCCTCC	21	Ca3	9477899	9477920	-	0	25.30	10.46	28.26	21.30	0	0.9	0.4	1	0.8
Car-miR-H14	TGAGGCACAGTCTAAGCGTGGT	22	Ca3	13390805	13390827	-	0	0	0	0	851.80	0	0	0	0	1
Car-miR-H15	GCACCAAATGCGTCAACTTCAA	22	Ca2	17120506	17120528	Yes	0	0	0	0	56.79	0	0	0	0	1
Car-miR-K12-12	TGTAATTCCAGGTGAACCAAT	21	Ca8	2509164	2509185	-	582.21	1053.20	1798.59	1031.38	894.39	0.3	0.6	1	0.6	0.5
Car-miR-M1-15	TCACCCTTTGCTGGTGAGTGC	21	Ca4	2721881	2721902	Yes	0	0	20.91	0	0	0	0	1	0	0
Car-miR-rL1-25	TGCATGGCACCAAATGGAAATTGT	24	Ca3	3365782	3365806	-	23.76	34.79	26.14	14.13	198.75	0.1	0.2	0.1	0.1	1
Car-novmiR1	GCGACGACTTCCACTTGGTGC	21	Ca1	13181144	13181165	-	249.52	148.65	209.14	324.96	220.05	0.8	0.5	0.6	1	0.7
Car-novmiR10	ACGCACCAATTTTCTATCAGA	21	Ca2	35718634	35718655	-	499.04	477.58	57.51	98.90	553.67	0.9	0.9	0.1	0.2	1
Car-novmiR11	TAGGGCTACCAAATAGGAAGTTCT	24	Ca3	18277790	18277814	-	23.76	9.49	26.14	14.13	7.10	0.9	0.4	1	0.5	0.3
Car-novmiR12	TGCTGGTTCATCTGTAGCAAT	21	Ca3	19472532	19472553	-	0	12.65	0	0	0	0	1	0	0	0
Car-novmiR13	CACCAAACCATCGACTGCTT	20	Ca3	24040171	24040191	Yes	11.88	0	0	0	0	1	0	0	0	0
Car-novmiR14	CGCAACATCAATGCACCAAAC	21	Ca3	29915207	29915228	-	2447.66	1470.69	993.41	1102.02	702.74	1	0.6	0.4	0.5	0.3
Car-	TTGTGGAGAGCTGTAGGCAC	20	Ca3	399402	399402	Yes	0	6.33	0.00	0.00	0.00	0	1	0	0	0

novmiR15				78	98										
Car-novmiR16	TGCACCATCGATCCACGTGAT	21	Ca4	3538780	3538801	-	0	0	5.23	0	0	0	0	1	0	0
Car-novmiR17	TCAAAGCCTCCAGCACCAAGAAAG	24	Ca4	4722116	4722140	-	47.53	44.28	0	0	28.39	1	0.9	0	0	0.6
Car-novmiR18	CTCGCCCAGGGTGGTACAG	19	Ca4	7529468	7529487	Yes	11.88	0	0	0	0	1	0	0	0	0
Car-novmiR19	AGCACCACCACCACCACCGG	20	Ca4	8659740	8659760	-	154.46	1233.48	941.12	84.77	205.85	0.1	1	0.8	0.1	0.2
Car-novmiR2	ACAGTGGTGCCTACAGCAAC	20	Ca1	27131053	27131073	Yes	59.41	88.56	41.83	42.39	347.82	0.2	0.3	0.1	0.1	1
Car-novmiR20	TCACCAAAGGTGCTCGGGA	19	Ca4	19748551	19748570	Yes	23.76	28.46	5.23	0	49.69	0.5	0.6	0.1	0	1
Car-novmiR21	TGCGCACCAAATACACCATCT	21	Ca4	22912681	22912702	-	0	0	0	0	7.10	0	0	0	0	1
Car-novmiR22	GAGGAATTTCTAGGGCCCAAA	21	Ca4	32422380	32422401	-	11.88	0	0	0	0	1	0	0	0	0
Car-novmiR23	TACAGAATCCTAGTTTGGTGC	21	Ca4	33926786	33926807	-	0	6.33	0	0	7.10	0	0.9	0	0	1
Car-novmiR24	TCAAGCACCAAATGTGGAAATT	22	Ca4	34462010	34462032	-	0	0	10.46	14.13	35.49	0	0	0.3	0.4	1
Car-novmiR25	TCGGAGAAGTCCACTTTGGTGTC	23	Ca4	41055254	41055277	-	0	9.49	5.23	0	7.10	0	1	0.6	0	0.7
Car-novmiR26	TGCACAAGCACCATGGATTCT	21	Ca4	41319078	41319099	-	0	3.16	0	0	0	0	1	0	0	0
Car-novmiR27	TCCTGGCGTCGGCCTGTGGGCTC	23	Ca5	18665100	18665123	Yes	47.53	22.14	41.83	14.13	127.77	0.4	0.2	0.3	0.1	1
Car-novmiR28	GGGCGCACCGGACACCACG	19	Ca5	18666256	18666275	Yes	0	9.49	0	0	42.59	0	0.2	0	0	1
Car-novmiR29	TGCAGACCAAGAAACTGTAGACAC	24	Ca5	20023840	20023864	-	0	0	0	0	7.10	0	0	0	0	1
Car-novmiR3	TGCTACAGCATTGCACGGGTCG	22	Ca1	32080665	32080687	Yes	118.82	22.14	62.74	0	85.18	1	0.2	0.5	0	0.7
Car-novmiR30	GGCCCGGGTACATATTCACCGC	22	Ca5	23664525	23664547	Yes	11.88	9.49	0	0	0	1	0.8	0	0	0
Car-novmiR31	TAGACGATAATGCTGTAGGGAC	22	Ca5	44699516	44699538	-	11.88	0	0	0	0	1	0	0	0	0
Car-novmiR32	TCGCACCATTCAACATGAAAC	21	Ca6	15133428	15133449	Yes	83.17	167.63	73.20	0	120.67	0.5	1	0.4	0	0.7
Car-novmiR33	TGCACCAAAGGTCACTGAAC	20	Ca6	26770120	26770140	Yes	0	0	0	0	7.09834041	0	0	0	0	1
Car-novmiR34	CCAAAATTCCATTGGTGCTTC	21	Ca6	30319594	30319615	Yes	95.05	256.18	303.25	155.41	305.23	0.3	0.8	1.0	0.5	1
Car-novmiR35	TCAGAGGTAGTGACAATTTTGAG	23	Ca7	10803992	10804015	Yes	11.88	6.33	0	0	0	1	0.5	0	0	0
Car-novmiR36	CCAAAGATATGACAATTTCGGTGC	24	Ca7	13904780	13904804	Yes	0	18.98	0	0	0	0	1	0	0	0

Car-novmiR37	TGGCACCACCAATAGCACCAA	21	Ca7	32162684	32162705	-	0	3.16	5.23	0	7.10	0	0.4	0.7	0	1
Car-novmiR38	AGAGAACACAGATGACACACA	21	Ca7	33257296	33257317	Yes	23.76	0	0	0	0	1	0	0	0	0
Car-novmiR39	GATGAATACAAATACAACT	19	Ca7	37814214	37814233	Yes	0	3.16	0	0	0	0	1	0	0	0
Car-novmiR4	TGCGTATCGACCCGTGCAATG	21	Ca1	32080673	32080694	Yes	95.05	18.98	26.14	0	35.49	1	0.2	0.3	0	0.4
Car-novmiR40	TCAGACAACGCAGACTGTAGGTTC	24	Ca7	39211331	39211355	Yes	0	0	0	0	7.10	0	0	0	0	1
Car-novmiR41	TAGCACCAAGACCAACAAAT	20	Ca7	40460777	40460797	-	0	0	0	0	7.10	0	0	0	0	1
Car-novmiR42	TAGGTGCACTGATGGTGCAG	20	Ca8	816890	816910	-	11.88	3.16	5.23	0	0	1	0.3	0.4	0	0
Car-novmiR43	TTCACCACTACAATCGTCGTC	21	Scaffold1193	96852	96873	-	0	0	10.46	0	7.10	0	0.0	1	0	0.7
Car-novmiR44	TGCCTACATTGTTCCATCGAC	21	Scaffold1202	251852	251873	Yes	11.88	28.46	5.23	14.13	156.16	0.1	0.2	0.0	0.1	1
Car-novmiR45	TTCCACCAATAGGAATTACTC	21	Scaffold198	71750	71771	Yes	142.58	82.23	120.25	211.93	198.75	0.7	0.4	0.6	1	0.9
Car-novmiR46	TAGAACACACACCATGGAATTC	22	Scaffold2	188956	188978	-	11.88	6.33	0	0	0	1	0.5	0	0	0
Car-novmiR47	TGCGACAAAGTGGGAATTCT	20	Scaffold223	175757	175777	-	59.41	15.81	20.91	0	14.20	1	0.3	0.4	0	0.2
Car-novmiR48	GAGCCCACAGGCCGACGCCAGGAG	24	Scaffold287	2576	2600	Yes	47.53	22.14	41.83	14.13	127.77	0.4	0.2	0.3	0.1	1
Car-novmiR49	TGGTCGATGGAACAATGTAGGCA	24	Scaffold287	22889	22913	Yes	11.88	28.46	5.23	14.13	156.16	0.1	0.2	0.0	0.1	1
Car-novmiR5	TCTTACAGAGAATTCCAGCAC	21	Ca1	33650191	33650212	Yes	1033.72	1233.48	1275.74	1426.98	3584.66	0.3	0.3	0.4	0.4	1
Car-novmiR50	GCCGACGCCAGGAGCGCGC	19	Scaffold2888	204	223	Yes	0	3.16	0	0	28.39	0	0.1	0	0	1
Car-novmiR51	TCCAAAACTCCCCCATCTGT	20	Scaffold396	125617	125637	-	0	0	5.23	0	0	0	0	1	0	0
Car-novmiR52	TCCCAGCACCAAATGGAATTA	21	Scaffold40	727403	727424	Yes	5988.45	1609.85	2703.11	4860.20	3669.84	1	0.3	0.5	0.8	0.6
Car-novmiR53	TAGAGAATCACCAGCACCAAA	21	Scaffold48	308761	308782	Yes	0	506.04	0	0	0	0	1	0	0	0
Car-novmiR54	GATTGATCGTGCCTACAAGA	20	Scaffold589	219120	219140	-	499.04	1084.83	1354.17	1045.51	887.29	0.4	0.8	1	0.8	0.7
Car-novmiR55	AGGAGAAGTCGTAACAAGGTTT	22	Scaffold604	6267	6289	Yes	23.76	25.30	20.91	0	14.20	0.9	1	0.8	0	0.6
Car-novmiR56	TCACATACAAGAAACCTTGTTACG	24	Scaffold604	72433	72457	Yes	11.88	6.33	41.83	14.13	0	0.3	0.2	1	0.3	0
Car-novmiR57	TCCCGACAGGTATGCTCTCACTC	23	Scaffold7279	133	156	Yes	118.82	335.25	0	56.51	198.75	0.4	1	0	0.2	0.6
Car-	ACGCTTTACGCCCAATCATTCC	22	Scaffold731	362233	362255	Yes	47.5	50.60	0	14.13	0	0.9	1	0	0.3	0

novmiR58							3									
Car-<br>novmiR59	TGAGAGCATACCTGTCGGGAC	21	Scaffold8751	891	912	-	118.<br>82	335.25	0	56.51	198.75	0.4	1	0	0.2	0.6
Car-<br>novmiR6	CCTACAAGCACCAAGGAGT	19	Ca2	153976<br>64	153976<br>83	-	0	60.09	20.91	14.13	42.59	0	1	0.3	0.2	0.7
Car-<br>novmiR60	TCGCGCACCATCACCAATGGA<br>ATT	24	*	0	0	Yes	11.8<br>8	25.30	10.46	0	7.10	0.5	1	0.4	0	0.3
Car-<br>novmiR7	TAGAAGAATTCCCACTTTGTTG<br>CA	24	Ca2	172263<br>38	172263<br>62	-	297.<br>05	234.04	245.74	367.34	149.07	0.8	0.6371<br>3	0.7	1	0.4
Car-<br>novmiR8	TAGCCAACTCCTTTTTCCCTT	21	Ca2	328528<br>15	328528<br>36	-	11.8<br>8	0	0	0	0	1	0	0	0	0
Car-<br>novmiR9	TCAGCACCAAAACTAGTAAGC	21	Ca2	344145<br>65	344145<br>86	-	368.<br>34	389.02	5.23	141.28	177.46	0.9	1	0.0	0.4	0.5

Supplementary Table S3- List of miRNAs located in coding region.

Name	Position	Start	End		
**Car-miR-10d**	Ca_LG_7	31643017	31643035	gene	CDS
**Car-miR-711**	Ca_LG_3	39677591	39677610	gene	three_prime_UTR
**Car-miR-4661**	Ca_LG_4	31178369	31178387	gene	three_prime_UTR
**Car-miR-422a**	Ca_LG_4	1352948	1352966	gene	three_prime_UTR
**Car-miR-6222**	Ca_LG_4	42774598	42774616	gene	Intron
**Car-miR-309a**	Ca4	10250571	10250590	gene	CDS
**Car-miR-2496.2**	Ca_LG_3	23222746	23222765	gene	Intron
**Car-miR-7031**	Ca_LG_6	52011529	52011547	gene	Intron
**Car-miR-6988**	Ca_LG_8	5574598	5574616	gene	CDS
**Car-miR-888**	Ca_LG_7	10341728	10341746	gene	CDS
**Car-miR-3478**	Ca_LG_1	802605	802624	gene	Intron
**Car-miR-7362**	Ca_LG_8	6858906	6858925	gene	Intron
**Car-miR-3982**	Ca_LG_4	54560480	54560498	gene	CDS
**Car-miR-4591**	scaffold04669	11393	11411	gene	CDS
**Car-miR-8274**	Ca_LG_1	16745393	16745412	gene	Intron
**Car-miR-7635**	Ca_LG_8	5762232	5762250	gene	Intron
**Car-miR-2690**	scaffold01327	42537	42558	gene	Intron

Supplementary Table S4-Predicted targets of newly identified miRNAs in chickpea.

miRNA_Acc.	Target_Acc.	NCBI_Acc.	Target Description
**Car-miR-9788.1**	Ca_02538.1|Ca_LG_3:4334686-4339906|minus|	gi|828301466|ref|XP_012569511.1|	Cicer arietinum potassium transporter 5-like
**Car-miR-9788.1**	Ca_11796.1|scaffold00279:54263-65312|minus|	gi|502142477|ref|XP_004504977.1|	Cicer arietinum pseudouridine-5'-monophosphatase-like
**Car-miR-2496.1**	Ca_01899.1|Ca_LG_2:13896917-13900718|plus|	gi|502143726|ref|XP_004505443.1|	Cicer arietinum thiamine thiazole synthase, chloroplastic-like
**Car-miR-2496.1**	Ca_05460.1|Ca_LG_5:3048101-3056596|minus|	gi|828312548|ref|XP_012571468.1|	Cicer arietinum phosphatidylinositol-glycan biosynthesis class F protein-like
**Car-miR-7035**	Ca_13782.2|scaffold00719:46236-58338|plus|	gi|571545385|ref|XP_006602352.1|	Cicer arietinum paired amphipathic helix protein Sin3-like 4-like
**Car-miR-7035**	Ca_15050.2|scaffold01135:49149-62753|minus|	gi|502131747|ref|XP_004501088.1|	Cicer arietinum uncharacterized LOC101498
**Car-novmiR19**	Ca_07615.1|Ca_LG_7:3377720-3377967|minus|	-	not found
**Car-novmiR19**	Ca_08256.3|Ca_LG_8:4485093-4486412|minus|	gi|828324055|ref|XP_012573300.1|	Cicer arietinum glycine-rich cell wall structural protein-like
**Car-novmiR19**	Ca_07662.1|Ca_LG_7:4408137-4409174|plus|	gi|760063750|ref|WP_043746609.1|	not found
**Car-novmiR19**	Ca_10338.1|scaffold01652:39921-42903|minus|	gi|828310713|ref|XP_012571131.1|	Cicer arietinum glycine-rich RNA-binding protein 3, mitochondrial-like
**Car-novmiR19**	Ca_20666.1|scaffold16481:226-1035|minus|	-	Cicer arietinum peptidyl-prolyl cis-trans isomerase B1-like
**Car-novmiR19**	Ca_13376.1|scaffold00606:107582-109469|plus|	gi|502145495|ref|XP_004506054.1|	Cicer arietinum putative uncharacterized protein DDB_G0277255-like
**Car-novmiR19**	Ca_05365.1|Ca_LG_5:2021417-2025075|minus|	gi|502131262|ref|XP_004500928.1|	Cicer arietinum uncharacterized LOC101502880
**Car-miR-399i.2**	Ca_04647	-	Cicer arietinum cysteine-rich receptor-like protein kinase 6-like
**Car-miR-7031**	Ca_03656.1|Ca_LG_3:20152381-20165047|plus|	gi|502159370|ref|XP_004511484.1|	Cicer arietinum putative esterase/lipase HI_0193-like
**Car-miR-7031**	Ca_07694.1|Ca_LG_7:5032141-5036906|minus|	gi|502154750|ref|XP_004509818.1|	Cicer arietinum proline-rich receptor-like protein kinase PERK13-like
**Car-miR-7031**	Ca_14943.1|scaffold01094:52532-55270|minus|	gi|502153982|ref|XP_004509533.1|	Cicer arietinum pentatricopeptide repeat-containing protein At4g19440
**Car-miR-7031**	Ca_14191.1|scaffold00823:106506-109326|minus|	gi|502119002|ref|XP_004496472.1|	Cicer arietinum trihelix transcription factor GT-2-like
**Car-miR-7031**	Ca_02006.1|Ca_LG_2:15150173-15152073|plus|	gi|502143035|ref|XP_004505192.1|	Cicer arietinum putative nuclease HARBI1-like
**Car-miR-7031**	Ca_01104.1|Ca_LG_2:1205316-1207824|minus|	gi|502168638|ref|XP_004514434.1|	Cicer arietinum endo-1,3(4)-beta-glucanase 1-like
**Car-miR-7031**	Ca_04845.1|Ca_LG_4:15863255-15866955|plus|	gi|502117125|ref|XP_004495712.1|	Cicer arietinum formin-like protein 11-like
**Car-miR-7031**	Ca_17556.1|scaffold02523:35003-36493|plus|	gi|502120506|ref|XP_004496984.1|	Cicer arietinum probable ribose-5-phosphate isomerase-like
**Car-miR-7031**	Ca_06634.1|Ca_LG_6:2771398-2776008|plus|	gi|828308674|ref|XP_012570718.1|	Cicer arietinum extensin-2-like
**Car-miR-7031**	Ca_12518.1|scaffold00409:37180-42195|plus|	gi|828331424|ref|XP_004512265.2|	Cicer arietinum rab proteins geranylgeranyltransferase component A 2-like
**Car-miR-7031**	Ca_16744.1|scaffold01972:14708-16011|minus|	gi|502081759|ref|XP_004486959.1|	Cicer arietinum extensin-2-like
**Car-miR-10d**	Ca_04128.1|Ca_LG_4:3526439-3546874|plus|	gi|828308636|ref|XP_012570708.1|	Cicer arietinum protein arginine N-methyltransferase 1.5-like
**Car-miR-10d**	Ca_10480.1|scaffold02308:15758-18730|minus|	gi|502102458|ref|XP_004492082.1|	Cicer arietinum metal tolerance protein 11-like
**Car-miR-K12-12**	Ca_06795.1|Ca_LG_6:4472373-4476949|plus|	gi|502139740|ref|XP_004503881.1|	probable LRR receptor-like serine/threonine-protein kinase At5g63710-like
**Car-miR-K12-12**	Ca_13127.2|scaffold00546:80439-86447|minus|	gi|502086882|ref|XP_004488356.1|	Cicer arietinum DEAD-box ATP-dependent RNA helicase 51-like
**Car-miR-4412**	Ca_00893.1|Ca_LG_1:11536253-11540271|plus|	gi|502082410|ref|XP_004487158.1|	Cicer arietinum myosin-9-like
**Car-miR-4412**	Ca_07364.3|Ca_LG_6:11285061-11288414|minus|	gi|502139392|ref|XP_004503750.1|	-
**Car-miR-4412**	Ca_08669.1|Ca_LG_8:9084718-9089495|minus|	gi|502175770|ref|XP_004515738.1|	Cicer arietinum monocopper oxidase-like protein SKU5-like
**Car-novmiR54**	Ca_15730.3|scaffold01405:24062-27145|minus|	gi|502120561|ref|XP_004497003.1|	Not found
**Car-novmiR54**	Ca_08669.1|Ca_LG_8:9084718-9089495|minus|	gi|502175770|ref|XP_004515738.1|	-
**Car-novmiR54**	Ca_07364.3|Ca_LG_6:11285061-11288414|minus|	gi|502139392|ref|XP_004503750.1|	-
**Car-novmiR56**	Ca_01761.1|Ca_LG_2:11235094-11238011|plus|	gi|828322176|ref|XP_004506700.2|	Cicer arietinum uncharacterized LOC101510948
**Car-novmiR56**	Ca_00610.1|Ca_LG_1:7952482-7957969|plus|	gi|502086233|ref|XP_004488152.1|	Cicer arietinum AT-rich interactive domain-containing protein 4-like
**Car-miR-2661**	Ca_14162.1|scaffold00815:61921-65292|plus|	gi|502134411|ref|XP_004502110.1|	Cicer arietinum zinc finger protein CONSTANS-LIKE 9-like

**Car-miR-2661**	Ca_11751.1|scaffold00271:180387-185742|plus|	gi|502118931|ref|XP_004496445.1|	Cicer arietinum histone-lysine N-methyltransferase ASHH1-like
**Car-miR-2661**	Ca_00448.1|Ca_LG_1:5328248-5334843|plus|	gi|502111902|ref|XP_004494200.1|	Cicer arietinum signal recognition particle 54 kDa protein, chloroplastic-like
**Car-miR-2661**	Ca_01915.1|Ca_LG_2:14069684-14070816|minus|	gi|828320095|ref|XP_012572615.1|	Medicago truncatula hypothetical protein
**Car-miR-7188**	Ca_25606.1|scaffold179716:507-857|plus|	gi|800937454|ref|WP_045965844.1|	unknown
**Car-miR-7188**	Ca_07466.1|Ca_LG_7:1009934-1053510|plus|	gi|828328546|ref|XP_012574078.1|	Cicer arietinum midasin-like
**Car-miR-6830**	Ca_06039.1|Ca_LG_5:9475486-9477108|minus|	gi|502136527|ref|XP_004502719.1|	Cicer arietinum homeobox-leucine zipper protein ATHB-12-like
**Car-miR-8949**	Ca_03160.1|Ca_LG_3:15003067-15011710|minus|	gi|828330537|ref|XP_012574443.1|	Cicer arietinum sec14 cytosolic factor-like
**Car-miR-1304**	Ca_12578.1|scaffold00420:8203-10909|minus|	gi|502097858|ref|XP_004491098.1|	Cicer arietinum transmembrane protein 256 homolog
**Car-miR-1304**	Ca_06539.1|Ca_LG_6:1507165-1528735|plus|	gi|502091866|ref|XP_004489655.1|	Cicer arietinum valine--tRNA ligase-like
**Car-miR-1304**	Ca_17044.1|scaffold02167:27920-31735|plus|	gi|502178716|ref|XP_004516319.1|	Cicer arietinum phytoene synthase, chloroplastic-like
**Car-miR-4865**	Ca_01845.1|Ca_LG_2:13002598-13004673|plus|	gi|502144373|ref|XP_004505673.1|	Cicer arietinum expansin-like A2-like
**Car-miR-4865**	Ca_18312.1|scaffold03341:23098-24036|plus|	gi|502166743|ref|XP_004513968.1|	Cicer arietinum putative DNA-binding protein ESCAROLA-like
**Car-miR-4865**	Ca_01020.1|Ca_LG_1:14136142-14153549|minus|	gi|502089626|ref|XP_004488969.1|	Cicer arietinum uncharacterized LOC101504865
**Car-miR-1421o**	Ca_02856.1|Ca_LG_3:10446209-10447079|plus|	gi|502106263|ref|XP_004492901.1|	Cicer arietinum uncharacterized LOC101500304
**Car-miR-874**	Ca_06399.1|Ca_LG_5:14621762-14625384|minus|	gi|502128660|ref|XP_004500038.1|	Cicer arietinum tryptophan synthase beta chain 2, chloroplastic-like
**Car-miR-874**	Ca_06399.2|Ca_LG_5:14621174-14625384|minus|	gi|502128660|ref|XP_004500038.1|	Cicer arietinum tryptophan synthase beta chain 2, chloroplastic-like
**Car-miR-7241.1**	Ca_11105.1|scaffold00159:183632-187237|plus|	gi|828301585|ref|XP_012569532.1|	Cicer arietinum F-box/LRR-repeat protein 3-like
**Car-miR-871**	Ca_16626.1|scaffold01898:28202-32247|minus|	gi|502163371|ref|XP_004512819.1|	Cicer arietinum putative transporter arsB-like
**Car-miR-871**	Ca_16626.5|scaffold01898:28202-32613|minus|	gi|502163371|ref|XP_004512819.1|	Cicer arietinum putative transporter arsB-like
**Car-miR-871**	Ca_08464.1|Ca_LG_8:6550668-6553677|plus|	gi|657390428|gb|KEH31949.1|	Cicer arietinum NEP1-interacting protein-like 2-like
**Car-miR-932**	Ca_01916.1|Ca_LG_2:14079267-14082996|plus|	gi|828320180|ref|XP_012572633.1|	Cicer arietinum clone Ca3 TIR-NBS-LRR disease resistance protein gene,
**Car-miR-6930**	Ca_04634.1|Ca_LG_4:10909679-10912262|plus|	gi|502119076|ref|XP_004496496.1|	Cicer arietinum L-type lectin-domain containing receptor kinase S.6-like
**Car-miR-6930**	Ca_10515.1|scaffold03726:52436-55174|minus|	gi|502155347|ref|XP_004510040.1|	Cicer arietinum transcription factor TCP4-like
**Car-miR-6930**	Ca_14830.1|scaffold01044:56059-58488|plus|	gi|502079318|ref|XP_004486223.1|	Cicer arietinum F-box protein CPR30-like
**Car-miR-6930**	Ca_08007.1|Ca_LG_8:352712-357682|minus|	gi|502126581|ref|XP_004499361.1|	Cicer arietinum UPF0420 protein C16orf58 homolog
**Car-miR-2151.1**	Ca_13505.1|scaffold00635:8856-11407|minus|	gi|502082784|ref|XP_004487272.1|	Probable L-type lectin-domain containing receptor kinase VII.2-like
**Car-miR-2151.1**	Ca_04410.1|Ca_LG_4:8160375-8161748|minus|	gi|502132307|ref|XP_004501296.1|	Cicer arietinum uncharacterized LOC101513393
**Car-miR-2151.1**	Ca_07088.1|Ca_LG_6:8020426-8023420|plus|	gi|702273422|ref|XP_010043898.1|	Citrus sinensis peptide transporter PTR2-like
**Car-miR-2151.1**	Ca_10435.1|scaffold02056:64040-68626|plus|	gi|502166974|ref|XP_004514026.1|	probable leucine-rich repeat receptor-like protein kinase At2g33170-like
**Car-miR-2151.1**	Ca_16217.1|scaffold01644:23325-23885|plus|	gi|828307214|ref|XP_012570453.1|	Cicer arietinum zinc finger protein 7-like
**Car-miR-2151.1**	Ca_08224.1|Ca_LG_8:4207132-4209303|plus|	gi|502150748|ref|XP_004508104.1|	Cicer arietinum probable protein phosphatase 2C 13-like
**Car-novmiR35**	Ca_16218.1|scaffold01644:32496-33077|plus|	gi|502122948|ref|XP_004497948.1|	Cicer arietinum zinc finger protein 7-like
**Car-novmiR35**	Ca_09432.1|scaffold05945:140716-141402|plus|	gi|502122948|ref|XP_004497948.1|	Cicer arietinum zinc finger protein 3-like
**Car-novmiR35**	Ca_04705.1|Ca_LG_4:11655306-11664650|plus|	gi|502118543|ref|XP_004496319.1|	-
**Car-miR-8322**	Ca_00436.1|Ca_LG_1:5226833-5230494|minus|	gi|502111984|ref|XP_004494214.1|	Cicer arietinum uncharacterized LOC101491958
**Car-miR-8322**	Ca_02170.2|Ca_LG_3:48838-53051|minus|	gi|502161469|ref|XP_004512176.1|	Cicer arietinum aspartic proteinase-like protein 1-like
**Car-miR-8322**	Ca_08912.1|scaffold00216:124852-131977|plus|	gi|502162799|ref|XP_004512620.1|	Cicer arietinum histone-lysine N-methyltransferase ASHH3-like
**Car-miR-8322**	Ca_01507.1|Ca_LG_2:6649187-6656859|minus|	gi|502098074|ref|XP_004491149.1|	Cicer arietinum abscisic-aldehyde oxidase-like
**Car-novmiR46**	Ca_10294.1|scaffold01499:196605-197102|minus|	gi|568864938|ref|XP_006485841.1|	not found
**Car-miR-2057**	Ca_20761.1|scaffold19560:573-938|minus|	gi|828334011|ref|XP_012575071.1|	Cicer arietinum uncharacterized LOC101496645
**Car-miR-2057**	Ca_19444.1|scaffold05689:6112-6504|plus|	gi|828334011|ref|XP_012575071.1|	-

**Car-miR-2057**	Ca_21555.1|scaffold84670:2-445|minus|	gi|748749171|ref|WP_040007287.1|	-
**Car-novmiR52**	Ca_08879.1|scaffold00212:58036-62657|plus|	gi|828332867|ref|XP_004513234.2|	Cicer arietinum flavonoid 3',5'-hydroxylase 2-like
**Car-novmiR52**	Ca_07573.1|Ca_LG_7:2839184-2843036|plus|	gi|502176358|ref|XP_004515856.1|	Cicer arietinum cytochrome P450 71A26-like
**Car-novmiR52**	Ca_04807.1|Ca_LG_4:12955370-12957248|minus|	gi|502118264|ref|XP_004496181.1|	Cicer arietinum cytochrome P450 77A3-like
**Car-novmiR52**	Ca_08937.1|scaffold05824:341346-343280|minus|	gi|502146991|ref|XP_004506686.1|	Cicer arietinum cytochrome P450 71D9-like
**Car-miR-5198**	Ca_11049.1|scaffold00157:162911-167206|minus|	gi|502140730|ref|XP_004504314.1|	Cicer arietinum importin-5-like
**Car-miR-5198**	Ca_20394.1|scaffold11467:3740-4063|plus|	gi|828339075|ref|XP_012567653.1|	Cicer arietinum protein TRANSPARENT TESTA 1-like
**Car-miR-5198**	Ca_20377.1|scaffold11271:2515-3087|minus|	gi|357517577|ref|XP_003629077.1|	not found
**Car-miR-361.1**	Ca_07404.1|Ca_LG_7:338459-357112|plus|	gi|502121886|ref|XP_004497478.1|	Cicer arietinum 2-isopropylmalate synthase 2
**Car-miR-361.1**	Ca_07550.1|Ca_LG_7:2635599-2636896|minus|	gi|502176512|ref|XP_004515877.1|	Cicer arietinum protein LATERAL ORGAN BOUNDARIES-like
**Car-miR-361.1**	Ca_04009.1|Ca_LG_4:1931891-1934172|minus|	gi|502125674|ref|XP_004499020.1|	Cicer arietinum F-box protein At1g47056-like
**Car-miR-4423.1**	Ca_04305.2|Ca_LG_4:6939616-6942756|minus|	gi|502179091|ref|XP_004516371.1|	Cicer arietinum eukaryotic translation initiation factor 3 subunit E-like
**Car-miR-4423.1**	Ca_04305.1|Ca_LG_4:6938913-6942756|minus|	gi|502179091|ref|XP_004516371.1|	Cicer arietinum eukaryotic translation initiation factor 3 subunit E-like
**Car-miR-4423.1**	Ca_05724.1|Ca_LG_5:6319670-6322605|minus|	gi|502134021|ref|XP_004501972.1|	Cicer arietinum protein ETHYLENE INSENSITIVE 3-like
**Car-miR-9788.2**	Ca_19523.1|scaffold05936:8391-9623|plus|	gi|502118646|ref|XP_004496354.1|	Cicer arietinum scarecrow-like protein 18-like
**Car-miR-9788.2**	Ca_02537.1|Ca_LG_3:4326547-4331470|minus|	gi|502112506|ref|XP_004494353.1|	Cicer arietinum uncharacterized LOC101506286
**Car-miR-9788.2**	Ca_02846.1|Ca_LG_3:10378555-10383327|plus|	gi|502106317|ref|XP_004492914.1|	Cicer arietinum ELMO domain-containing protein A-like
**Car-miR-7154**	Ca_15120.1|scaffold01166:85859-86893|minus|	gi|502093888|ref|XP_004490085.1|	Cicer arietinum uncharacterized LOC101504118
**Car-miR-7154**	Ca_07738.1|Ca_LG_7:5654190-5656704|minus|	gi|502154445|ref|XP_004509702.1|	Cicer arietinum caffeic acid 3-O-methyltransferase 1-like
**Car-miR-2496.2**	Ca_15631.1|scaffold01374:9325-10575|plus|	gi|502127724|ref|XP_004499811.1|	Cicer arietinum transcription factor MYB98-like
**Car-miR-2496.2**	Ca_08756.1|scaffold00046:192192-193566|minus|	gi|502152988|ref|XP_004509179.1|	Cicer arietinum dof zinc finger protein DOF5.7-like
**Car-miR-2496.2**	Ca_04734.2|Ca_LG_4:12035623-12041682|plus|	gi|502118339|ref|XP_004496218.1|	Cicer arietinum peptidyl-prolyl cis-trans isomerase CYP21-3
**Car-miR-20b**	Ca_10739.2|scaffold00078:380833-383738|plus|	gi|502091005|ref|XP_004489411.1|	Cicer arietinum transcription factor MYB28-like
**Car-miR-20b**	Ca_10739.1|scaffold00078:380833-383738|plus|	gi|502091005|ref|XP_004489411.1|	Cicer arietinum transcription factor MYB28-like
**Car-miR-319b**	Ca_20001.1|scaffold08079:2907-3071|minus|	gi|828297240|ref|XP_012568796.1|	Cicer arietinum uncharacterized LOC101494974
**Car-miR-319b**	Ca_11792.1|scaffold00277:187268-192555|plus|	gi|828320419|ref|XP_012572678.1|	Cicer arietinum protein Mpv17-like
**Car-novmiR12**	Ca_01313.1|Ca_LG_2:3799627-3802810|minus|	gi|502097270|ref|XP_004490953.1|	Cicer arietinum probable signal peptidase complex subunit 2-like
**Car-novmiR12**	Ca_04753.1|Ca_LG_4:12276014-12282706|plus|	gi|502118398|ref|XP_004496247.1|	Cicer arietinum pumilio homolog 4-like
**Car-miR-7734**	Ca_19571.1|scaffold06082:121-10005|minus|	gi|502111573|ref|XP_004494098.1|	Cicer arietinum uncharacterized LOC101512705
**Car-novmiR15**	Ca_05201.1|Ca_LG_4:21758140-21762580|minus|	gi|502121024|ref|XP_004497162.1|	Cicer arietinum proteasome subunit alpha type-5-like
**Car-novmiR15**	Ca_10078.1|scaffold01208:35949-59125|minus|	gi|828297442|ref|XP_012568840.1|	Cicer arietinum auxin transport protein BIG-like
**Car-miR-M1-15**	Ca_16499.1|scaffold01828:42198-58836|minus|	gi|502165216|ref|XP_004513474.1|	Cicer arietinum RNA polymerase-associated protein CTR9 homolog
**Car-novmiR16**	Ca_20109	-	Cicer arietinum interactor of constitutive active ROPs 2, chloroplastic-like
**Car-miR-7907b.2**	Ca_16618.1|scaffold01895:21376-23937|minus|	gi|502135863|ref|XP_004502479.1|	Cicer arietinum NAC domain-containing protein 2-like
**Car-miR-2039**	Ca_02753.1|Ca_LG_3:7241434-7244543|minus|	gi|502109551|ref|XP_004493671.1|	Cicer arietinum pentatricopeptide repeat-containing protein At3g60050-like
**Car-miR-2039**	Ca_10012.1|scaffold01129:44536-49180|plus|	gi|502128725|ref|XP_004500060.1|	Cicer arietinum uncharacterized LOC101509310
**Car-miR-2039**	Ca_16875.1|scaffold02074:34258-41148|minus|	gi|828337350|ref|XP_012567315.1|	Cicer arietinum uncharacterized LOC101503385
**Car-miR-422a**	Ca_04704.2|Ca_LG_4:11645496-11648531|minus|	gi|502118489|ref|XP_004496292.1|	Cicer arietinum serine carboxypeptidase-like 27-like
**Car-miR-422a**	Ca_04280.1|Ca_LG_4:6268141-6277967|plus|	gi|502122597|ref|XP_004497823.1|	Cicer arietinum probable actin-related protein 2/3 complex subunit 2-like
**Car-miR-422a**	Ca_22583.1|scaffold174900:42-221|minus|	gi|674646764|emb|CEA09604.1|	unknown
**Car-miR-29**	Ca_03255.2|Ca_LG_3:16243027-16249816|minus|	gi|357482377|ref|XP_003611474.1|	Cicer arietinum AP-2 complex subunit mu-like

**Car-miR-29**	Ca_03255.1|Ca_LG_3:16242462-16250070|minus|	gi|502160553|ref|XP_004511783.1|	Cicer arietinum AP-2 complex subunit mu-like
**Car-miR-6562.1**	Ca_20062.1|scaffold08578:1173-1568|minus|	gi|502088335|ref|XP_004488796.1|	Cicer arietinum putative Peroxidase 48-like
**Car-miR-6562.1**	Ca_10725.1|scaffold00078:74777-83919|minus|	gi|502090922|ref|XP_004489384.1|	Cicer arietinum ABC transporter B family member 19-like
**Car-miR-172e**	Ca_02287.4|Ca_LG_3:1402521-1405602|minus|	gi|502112749|ref|XP_004494431.1|	Cicer arietinum DEAD-box ATP-dependent RNA helicase 24-like
**Car-miR-172e**	Ca_02287.2|Ca_LG_3:1402521-1407233|minus|	gi|502112752|ref|XP_004494432.1|	Cicer arietinum DEAD-box ATP-dependent RNA helicase 24-like
**Car-miR-172e**	Ca_02287.1|Ca_LG_3:1402521-1407233|minus|	gi|502112749|ref|XP_004494431.1|	Cicer arietinum DEAD-box ATP-dependent RNA helicase 24-like
**Car-miR-172e**	Ca_02287.3|Ca_LG_3:1402521-1407233|minus|	gi|502112749|ref|XP_004494431.1|	Cicer arietinum DEAD-box ATP-dependent RNA helicase 24-like
**Car-miR-2575**	Ca_00241.1|Ca_LG_1:2344448-2345872|plus|	gi|502077890|ref|XP_004485777.1|	Cicer arietinum E3 ubiquitin-protein ligase RMA1H1-like
**Car-miR-8492**	Ca_03866.1|Ca_LG_3:23162311-23172924|minus|	gi|502162537|ref|XP_004512535.1|	Cicer arietinum probable starch synthase 4, chloroplastic/amyloplastic-like
**Car-miR-8492**	Ca_05574.1|Ca_LG_5:4788819-4790852|plus|	gi|502132600|ref|XP_004501440.1|	Cicer arietinum hybrid signal transduction histidine kinase A-like
**Car-miR-8492**	Ca_07442.1|Ca_LG_7:743891-748573|plus|	gi|502141946|ref|XP_004504716.1|	Cicer arietinum putative disease resistance protein RGA4-like
**Car-miR-8492**	Ca_16016.1|scaffold01531:55588-63386|plus|	gi|502156560|ref|XP_004510535.1|	Cicer arietinum uncharacterized LOC101505495
**Car-miR-5258**	Ca_11043.1|scaffold00157:114995-116695|minus|	gi|502140700|ref|XP_004504304.1|	Cicer arietinum mitochondrial dicarboxylate/tricarboxylate transporter DTC-like
**Car-miR-5258**	Ca_08071.1|Ca_LG_8:966817-969601|minus|	gi|502149977|ref|XP_004507733.1|	Cicer arietinum probable LRR receptor-like serine/threonine-protein kinase At2g24230-like
**Car-miR-5258**	Ca_03484.1|Ca_LG_3:18697303-18703454|plus|	gi|502159946|ref|XP_004511583.1|	Cicer arietinum putative copper-transporting ATPase HMA5-like
**Car-miR-1422c**	Ca_17156.1|scaffold02242:16501-18675|minus|	gi|40850570|gb|AAR96002.1|	Cicer reticulatum Ty1-copia retrotransposon reverse transcriptase-like gene
**Car-miR-1422c**	Ca_15061.1|scaffold01137:34908-40242|plus|	gi|502145832|ref|XP_004506199.1|	Cicer arietinum uncharacterized LOC101498524
**Car-miR-1422c**	Ca_10252.1|scaffold01437:43716-51528|plus|	gi|502086144|ref|XP_004488123.1|	Cicer arietinum 3-oxoacyl-[acyl-carrier-protein] synthase II, chloroplastic-like
**Car-miR-3629a**	Ca_00828.1|Ca_LG_1:10938180-10943887|plus|	gi|502083407|ref|XP_004487450.1|	Cicer arietinum transcription factor LHW-like
**Car-miR-3629a**	Ca_04253.1|Ca_LG_4:5610007-5614189|minus|	gi|502121999|ref|XP_004497529.1|	Cicer arietinum cell division cycle protein 48 homolog
**Car-miR-6612.2**	Ca_01727.1|Ca_LG_2:10207394-10215041|minus|	gi|502146307|ref|XP_004506396.1|	Cicer arietinum ran-binding protein 10-like
**Car-miR-6612.2**	Ca_00267.1|Ca_LG_1:2510659-2515166|minus|	gi|502077787|ref|XP_004485744.1|	Cicer arietinum serine-rich adhesin for platelets-like
**Car-miR-6612.2**	Ca_02969.1|Ca_LG_3:11747698-11751923|minus|	gi|502104643|ref|XP_004492601.1|	Cicer arietinum uncharacterized LOC101510671
**Car-miR-727.2**	Ca_09099.1|scaffold03204:59265-63821|minus|	gi|502094572|ref|XP_004490249.1|	Cicer arietinum dentin sialophosphoprotein-like
**Car-miR-727.2**	Ca_07851.1|Ca_LG_7:6979348-6985108|plus|	gi|502152692|ref|XP_004509046.1|	Cicer arietinum putative nuclear matrix constituent protein 1-like protein-like
**Car-miR-5466**	Ca_10330.1|scaffold01625:38905-55964|minus|	gi|502157333|ref|XP_004510838.1|	Cicer arietinum NAD-dependent malic enzyme 59 kDa isoform, mitochondrial-like
**Car-miR-5466**	Ca_05724.1|Ca_LG_5:6319670-6322605|minus|	gi|502134021|ref|XP_004501972.1|	-
**Car-miR-5466**	Ca_11378.1|scaffold00208:75934-79345|plus|	gi|502184186|ref|XP_004517302.1|	Cicer arietinum putative disease resistance RPP13-like protein 1-like
**Car-novmiR36**	Ca_13159.1|scaffold00553:137148-139161|plus|	gi|525314167|ref|NP_001265963.1|	Cicer arietinum tubby-like F-box protein 5-like
**Car-novmiR36**	Ca_05994.1|Ca_LG_5:9032536-9034800|plus|	gi|502136777|ref|XP_004502829.1|	Cicer arietinum chalcone--flavonone isomerase-like
**Car-novmiR36**	Ca_03362.1|Ca_LG_3:17639360-17642183|minus|	gi|502160202|ref|XP_004511667.1|	Cicer arietinum adenine phosphoribosyltransferase 1, chloroplastic-like
**Car-miR-3079**	Ca_01349.1|Ca_LG_2:4513319-4518419|plus|	gi|828294435|ref|XP_012568297.1|	Medicago truncatula COBW domain-containing protein
**Car-miR-3079**	Ca_05955.2|Ca_LG_5:8677301-8681432|plus|	gi|502136631|ref|XP_004502761.1|	Cicer arietinum two-component response regulator-like APRR5-like
**Car-miR-7433**	Ca_10940.1|scaffold00119:262215-262964|minus|	gi|502163690|ref|XP_004512918.1|	Cicer arietinum ankyrin repeat-containing protein kinase A-like
**Car-miR-7433**	Ca_05325.1|Ca_LG_5:1614346-1618865|minus|	gi|502131100|ref|XP_004500875.1|	Cicer arietinum protein NLP4-like
**Car-miR-675**	Ca_12296.1|scaffold00360:22254-26849|minus|	gi|502083897|ref|XP_004487598.1|	Cicer arietinum DDRGK domain-containing protein 1-like
**Car-miR-3478**	Ca_02306.1|Ca_LG_3:1552463-1555868|plus|	gi|502112692|ref|XP_004494413.1|	Cicer arietinum uncharacterized LOC101497500
**Car-miR-3478**	Ca_15962.1|scaffold01511:55085-56543|plus|	gi|502090007|ref|XP_004489091.1|	Cicer arietinum putative UDP-rhamnose:rhamnosyltransferase 1-like

**Car-miR-7362**	Ca_17419.1|scaffold02422:16595-23279|minus|	gi|502173574|ref|XP_004515361.1|	Cicer arietinum plasma membrane ATPase 1-like
**Car-miR-7362**	Ca_10330.1|scaffold01625:38905-55964|minus|	gi|502157333|ref|XP_004510838.1|	-
**Car-miR-7362**	Ca_08994.2|scaffold00233:211964-216244|plus|	gi|828320448|ref|XP_012572682.1|	Medicago truncatula Glucosidase 2 subunit beta
**Car-miR-967**	Ca_10841.2|scaffold00105:62387-69986|minus|	gi|502161785|ref|XP_004512283.1|	Cicer arietinum serine/threonine-protein kinase ppk4-like
**Car-miR-967**	Ca_10841.1|scaffold00105:62387-70033|minus|	gi|502161785|ref|XP_004512283.1|	-
**Car-miR-967**	Ca_13914.1|scaffold00751:30134-33896|minus|	gi|502159994|ref|XP_004511599.1|	Cicer arietinum cytochrome P450 87A3-like
**Car-miR-4864**	Ca_07900.1|Ca_LG_7:7749778-7751442|plus|	gi|828328477|ref|XP_012574062.1|	Cicer arietinum nodulation-signaling pathway 1 protein-like
**Car-miR-4864**	Ca_04263.1|Ca_LG_4:5745547-5749913|plus|	gi|502088191|ref|XP_004488763.1|	Cicer arietinum phosphoglycerate kinase, chloroplastic-like
**Car-miR-495**	Ca_16389.1|scaffold01748:23264-27049|plus|	gi|502180322|ref|XP_004516612.1|	Cicer arietinum uncharacterized LOC101509187
**Car-miR-495**	Ca_07116.1|Ca_LG_6:8479567-8483632|minus|	gi|502142523|ref|XP_004504999.1|	Cicer arietinum uncharacterized LOC101514166
**Car-miR-495**	Ca_00600.1|Ca_LG_1:7653658-7655816|minus|	gi|502087493|ref|XP_004488551.1|	Cicer arietinum uncharacterized LOC101507229
**Car-miR-7647**	Ca_17128.1|scaffold02225:23479-35697|minus|	gi|828338854|ref|XP_012567604.1|	Cicer arietinum RNA polymerase-associated protein LEO1-like
**Car-miR-7647**	Ca_13055.1|scaffold00523:109487-111228|minus|	gi|502096974|ref|XP_004490877.1|	Cicer arietinum glycerol-3-phosphate acyltransferase 5-like
**Car-novmiR51**	Ca_02499.1|Ca_LG_3:4014864-4019795|plus|	gi|502112340|ref|XP_004494299.1|	Cicer arietinum calcium homeostasis endoplasmic reticulum protein-like
**Car-novmiR53**	Ca_11371.1|scaffold00207:178922-181171|plus|	gi|502090841|ref|XP_004489358.1|	Cicer arietinum probable inactive receptor kinase At1g27190-like
**Car-novmiR53**	Ca_17898.1|scaffold02827:30916-36482|minus|	gi|502093210|ref|XP_004489873.1|	Cicer arietinum WAT1-related protein At3g28070-like
**Car-novmiR53**	Ca_00523.1|Ca_LG_1:6135242-6143327|minus|	gi|502080174|ref|XP_004486482.1|	Cicer arietinum sporulation-specific protein 15-like
**Car-miR-821d**	Ca_06597.1|Ca_LG_6:2301893-2305663|plus|	gi|502137651|ref|XP_004503151.1|	Cicer arietinum protein FIZZY-RELATED 3-like
**Car-miR-821d**	Ca_07015.1|Ca_LG_6:7153929-7158258|minus|	gi|502141598|ref|XP_004504559.1|	Cicer arietinum deoxyhypusine synthase-like
**Car-miR-821d**	Ca_17787.1|scaffold02717:25524-31637|plus|	gi|728835242|gb|KHG14685.1|	Cicer arietinum serine/threonine-protein kinase ULK4-like
**Car-miR-7492o**	Ca_03729.1|Ca_LG_3:21348562-21352295|minus|	gi|502161923|ref|XP_004512330.1|	Cicer arietinum decapping 5-like protein-like
**Car-miR-7492o**	Ca_04705.1|Ca_LG_4:11655306-11664650|plus|	gi|502118543|ref|XP_004496319.1|	Cicer arietinum uncharacterized LOC101504689
**Car-miR-549a**	Ca_16414.1|scaffold01774:17856-22250|plus|	gi|357480557|ref|XP_003610564.1|	Medicago truncatula Mo25 family protein
**Car-miR-937**	Ca_18777.1|scaffold04104:11643-12718|plus|	gi|828336672|ref|XP_012575679.1|	Cicer arietinum vegetative cell wall protein gp1-like
**Car-miR-937**	Ca_15487.1|scaffold01313:49287-54653|minus|	gi|502094063|ref|XP_004490130.1|	Cicer arietinum chorismate mutase, chloroplastic-like
**Car-miR-2570**	Ca_08785.1|scaffold00055:69812-74963|plus|	gi|502157000|ref|XP_004510724.1|	Cicer arietinum glyoxylate/succinic semialdehyde reductase 1-like
**Car-miR-430b**	Ca_07397.1|Ca_LG_7:179927-219756|plus|	gi|502121609|ref|XP_004497386.1|	Cicer arietinum callose synthase 9-like
**Car-miR-5507**	Ca_18410.1|scaffold03493:339-6441|minus|	gi|502087396|ref|XP_004488520.1|	Cicer arietinum CBL-interacting serine/threonine-protein kinase 23-like
**Car-miR-1144a.1**	Ca_03455.1|Ca_LG_3:18347785-18348593|plus|	gi|502160872|ref|XP_004511937.1|	Cicer arietinum calmodulin-like protein 1-like
**Car-miR-1144a.1**	Ca_03738.1|Ca_LG_3:21459140-21460835|plus|	gi|502162197|ref|XP_004512424.1|	Cicer arietinum anthocyanin 3'-O-beta-glucosyltransferase-like
**Car-miR-6539**	Ca_10155.1|scaffold01344:15203-28265|plus|	gi|502128969|ref|XP_004500139.1|	Cicer arietinum uncharacterized LOC101510274
**Car-miR-6539**	Ca_17352.1|scaffold02381:25542-29745|plus|	gi|502179233|ref|XP_004516395.1|	Cicer arietinum uncharacterized LOC101500211
**Car-miR-7276**	Ca_14333.1|scaffold00870:39423-43711|minus|	gi|502114561|ref|XP_004494986.1|	Cicer arietinum UDP-glucuronic acid decarboxylase 1-like
**Car-miR-B14RC**	Ca_14619.1|scaffold00964:54248-60754|plus|	gi|502102293|ref|XP_004492028.1|	Cicer arietinum RNA polymerase II C-terminal domain phosphatase-like 3-like
**Car-miR-B14RC**	Ca_01898.1|Ca_LG_2:13886842-13887751|plus|	gi|763790707|gb|KJB57703.1|	unknown
**Car-miR-B14RC**	Ca_00198.1|Ca_LG_1:1894844-1898242|plus|	gi|502077227|ref|XP_004485586.1|	Cicer arietinum uncharacterized LOC101515692
**Car-miR-2496.3**	Ca_14146.1|scaffold00812:48792-53243|plus|	gi|502173101|ref|XP_004515252.1|	Cicer arietinum uncharacterized LOC101509715
**Car-miR-2496.3**	Ca_18910.1|scaffold04360:17712-19308|minus|	gi|502092166|ref|XP_004489727.1|	Cicer arietinum probable S-acyltransferase At5g50020-like
**Car-miR-5033**	Ca_04643.1|Ca_LG_4:10985156-10989303|minus|	gi|502120037|ref|XP_004496826.1|	G-type lectin S-receptor-like serine/threonine-protein kinase At5g24080-like
**Car-miR-5033**	Ca_16758.1|scaffold01978:53292-53993|plus|	gi|502119960|ref|XP_004496799.1|	Cicer arietinum agamous-like MADS-box protein AGL62-like
**Car-miR-399i.1**	Ca_12767.1|scaffold00469:159056-160601|minus|	gi|502176641|ref|XP_004515907.1|	Cicer arietinum S-adenosylmethionine synthase 2-like

**Car-miR-138.2**	Ca_09486.1|scaffold04235:60951-66522|minus|	gi|502146758|ref|XP_004506594.1|	Cicer arietinum xylosyltransferase 2-like
**Car-miR-361.3**	Ca_02231.1|Ca_LG_3:900296-903556|plus|	gi|502112979|ref|XP_004494504.1|	Cicer arietinum pentatricopeptide repeat-containing protein At3g49170
**Car-miR-361.3**	Ca_08774.1|scaffold00046:530611-531220|minus|	gi|502153118|ref|XP_004509223.1|	Cicer arietinum STS14 protein-like
**Car-miR-1271**	Ca_03423.1|Ca_LG_3:18034745-18038842|minus|	gi|502160978|ref|XP_004511977.1|	Cicer arietinum L-ascorbate oxidase homolog
**Car-miR-1271**	Ca_13559.1|scaffold00644:83561-88087|minus|	gi|502153369|ref|XP_004509317.1|	Cicer arietinum protein transport protein Sec61 subunit alpha-like
**Car-novmiR24**	Ca_03824.1|Ca_LG_3:22735362-22738146|plus|	gi|502127291|ref|XP_004499639.1|	Cicer arietinum sugar transport protein 14-like
**Car-novmiR24**	Ca_01837.1|Ca_LG_2:12833372-12845889|plus|	gi|828320611|ref|XP_012572705.1|	Glycine max uncharacterized LOC100812718
**Car-novmiR24**	Ca_01837.3|Ca_LG_2:12833089-12848074|plus|	gi|828320611|ref|XP_012572705.1|	Glycine max uncharacterized LOC100812718
**Car-novmiR24**	Ca_01837.2|Ca_LG_2:12833372-12848450|plus|	gi|828320611|ref|XP_012572705.1|	Glycine max uncharacterized LOC100812718
**Car-novmiR24**	Ca_03927.1|Ca_LG_4:674132-677424|plus|	gi|502125978|ref|XP_004499138.1|	Cicer arietinum uncharacterized LOC101506316
**Car-novmiR25**	Ca_04132.1|Ca_LG_4:3611518-3614751|plus|	gi|502124503|ref|XP_004498555.1|	Cicer arietinum probable dimethyladenosine transferase-like
**Car-novmiR25**	Ca_00165.3|Ca_LG_1:1653653-1656659|plus|	gi|502076697|ref|XP_004485430.1|	Cicer arietinum elongation factor 1-gamma-like
**Car-novmiR25**	Ca_00165.2|Ca_LG_1:1653634-1656659|plus|	gi|502076697|ref|XP_004485430.1|	Cicer arietinum elongation factor 1-gamma-like
**Car-novmiR25**	Ca_00165.1|Ca_LG_1:1653368-1656659|plus|	gi|502076697|ref|XP_004485430.1|	Cicer arietinum elongation factor 1-gamma-like
**Car-novmiR25**	Ca_00165.4|Ca_LG_1:1653368-1656659|plus|	gi|502076697|ref|XP_004485430.1|	Cicer arietinum elongation factor 1-gamma-like
**Car-miR-58b**	Ca_18207.1|scaffold03186:20519-21229|plus|	gi|502143591|ref|XP_004505394.1|	Cicer arietinum LOB domain-containing protein 12-like
**Car-miR-58b**	Ca_06787.1|Ca_LG_6:4377783-4380806|minus|	gi|502090301|ref|XP_004489184.1|	Cicer arietinum uncharacterized LOC101499211
**Car-miR-2672**	Ca_00383.1|Ca_LG_1:3769291-3772838|plus|	gi|502078578|ref|XP_004485991.1|	Cicer arietinum uncharacterized LOC101505254
**Car-miR-2672**	Ca_07364.3|Ca_LG_6:11285061-11288414|minus|	gi|502139392|ref|XP_004503750.1|	Cicer arietinum UDP-glucose 4-epimerase-like
**Car-miR-9055.1**	Ca_19643.1|scaffold06355:2371-8929|minus|	gi|502087239|ref|XP_004488472.1|	Cicer arietinum cleavage stimulation factor subunit 1-like
**Car-miR-9055.1**	Ca_13579.1|scaffold00649:70763-85296|plus|	gi|828337186|ref|XP_012567281.1|	Cicer arietinum pleiotropic drug resistance protein 2-like
**Car-miR-7241.2**	Ca_13528.1|scaffold00637:122809-124527|plus|	gi|828327118|ref|XP_012573805.1|	Cicer arietinum fibroin heavy chain-like
**Car-miR-7241.2**	Ca_11196.1|scaffold00189:5707-6459|minus|	gi|828327762|ref|XP_012573938.1|	Cicer arietinum probable nucleoredoxin 1-like
**Car-miR-6612.1**	Ca_02267.1|Ca_LG_3:1197892-1206282|plus|	gi|502112844|ref|XP_004494461.1|	Cicer arietinum squamosa promoter-binding-like protein 12-like
**Car-miR-6612.1**	Ca_16809.1|scaffold02025:30985-32211|plus|	gi|502091392|ref|XP_004489536.1|	Cicer arietinum uncharacterized LOC101510754
**Car-novmiR34**	Ca_18187.1|scaffold03171:5634-8219|minus|	gi|502145681|ref|XP_004506131.1|	Cicer arietinum histone deacetylase HDT1-like
**Car-novmiR34**	Ca_18187.2|scaffold03171:4541-8219|minus|	gi|502145681|ref|XP_004506131.1|	Cicer arietinum histone deacetylase HDT1-like
**Car-novmiR34**	Ca_18588.1|scaffold03759:1251-2231|plus|	gi|828337460|ref|XP_012567346.1|	Cicer arietinum F-box/LRR-repeat protein 13-like
**Car-miR-15c**	Ca_02635.1|Ca_LG_3:6127181-6131050|plus|	gi|502110987|ref|XP_004493928.1|	Cicer arietinum glutathione S-transferase L3-like
**Car-miR-15c**	Ca_10855.2|scaffold00105:282135-285121|plus|	gi|357484287|ref|XP_003612431.1|	Cicer arietinum uncharacterized LOC101507155
**Car-miR-171**	Ca_06889.1|Ca_LG_6:5829237-5834012|plus|	gi|502140029|ref|XP_004504025.1|	Cicer arietinum uncharacterized LOC101491153
**Car-miR-171**	Ca_20014.1|scaffold08257:3766-6647|plus|	gi|502116617|ref|XP_004495517.1|	Cicer arietinum nuclear pore complex protein Nup98-Nup96-like
**Car-miR-215**	Ca_18371.1|scaffold03432:21717-26855|plus|	gi|502169513|ref|XP_004514624.1|	DNA-directed RNA polymerases I, II, and III subunit RPABC1-like
**Car-miR-215**	Ca_02328.1|Ca_LG_3:1720912-1724329|minus|	gi|502095118|ref|XP_004490378.1|	Cicer arietinum serine/threonine-protein kinase SAPK2-like
**Car-miR-9055.2**	Ca_17728.1|scaffold02667:21491-26776|plus|	gi|502156849|ref|XP_004510669.1|	Cicer arietinum uncharacterized LOC101494537
**Car-miR-9055.2**	Ca_01234.1|Ca_LG_2:2731928-2736969|plus|	gi|502092991|ref|XP_004489848.1|	Cicer arietinum protein SAMHD1 homolog
**Car-miR-9055.2**	Ca_00453.1|Ca_LG_1:5367492-5367968|plus|	gi|828301231|ref|XP_012569476.1|	Cicer arietinum uncharacterized LOC101511310
**Car-novmiR37**	Ca_15787.1|scaffold01429:66074-68762|minus|	gi|502165385|ref|XP_004513556.1|	G-type lectin S-receptor-like serine/threonine-protein kinase At2g19130-like
**Car-novmiR37**	Ca_09724.1|scaffold00829:9402-13326|plus|	gi|502127379|ref|XP_004499683.1|	Cicer arietinum uncharacterized LOC101512845
**Car-novmiR37**	Ca_18777.1|scaffold04104:11643-12718|plus|	gi|828336672|ref|XP_012575679.1|	-
**Car-novmiR37**	Ca_15910.1|scaffold01485:2501-8059|plus|	gi|502142382|ref|XP_004504930.1|	Cicer arietinum WAT1-related protein At1g68170-like

**Car-novmiR37**	Ca_09075.1|scaffold00333:29358-31696|minus|	gi|502144840|ref|XP_004505768.1|	Glycine max eukaryotic translation initiation factor 3 subunit A-like
**Car-miR-2333**	Ca_08382	-	Cicer arietinum centromere protein F-like
**Car-miR-6988**	Ca_09127.2|scaffold04453:69832-71682|plus|	gi|502091322|ref|XP_004489514.1|	Cicer arietinum uncharacterized LOC101500482
**Car-miR-6988**	Ca_12891.2|scaffold00495:63635-68611|plus|	gi|828307771|ref|XP_012570569.1|	Cicer arietinum uncharacterized LOC101506418
**Car-miR-6988**	Ca_04735.1|Ca_LG_4:12055341-12059247|plus|	gi|502118343|ref|XP_004496220.1|	Cicer arietinum transcription factor bHLH133-like
**Car-miR-7217**	Ca_19404.2|scaffold05577:6612-11717|minus|	gi|828320404|ref|XP_004505563.2|	Cicer arietinum putative DNA repair and recombination protein RAD26-like
**Car-miR-7217**	Ca_19404.1|scaffold05577:6612-11717|minus|	gi|828320404|ref|XP_004505563.2|	Cicer arietinum putative DNA repair and recombination protein RAD26-like
**Car-miR-7217**	Ca_07685.1|Ca_LG_7:4888996-4890978|plus|	gi|828327459|ref|XP_012573867.1|	Cicer arietinum pentatricopeptide repeat-containing protein At4g33990-like
**Car-novmiR43**	Ca_12123.1|scaffold00337:13433-13588|minus|	gi|607892108|gb|EZF31594.1|	Cicer arietinum protein FON2 SPARE1-like
**Car-novmiR43**	Ca_09125.1|scaffold04453:20945-21181|plus|	gi|354546089|emb|CCE42818.1|	Not found
**Car-novmiR43**	Ca_08658.2|Ca_LG_8:8961918-8965455|plus|	gi|502175863|ref|XP_004515755.1|	Cicer arietinum TVP38/TMEM64 family membrane protein slr0305-like
**Car-novmiR43**	Ca_11562.1|scaffold00252:133414-136651|plus|	gi|502174848|ref|XP_004515613.1|	Cicer arietinum putative kinase-like protein TMKL1-like
**Car-novmiR43**	Ca_05478.2|Ca_LG_5:3242487-3277135|plus|	gi|828313518|ref|XP_012571617.1|	Glycine max protein DDB_G0276689-like
**Car-miR-4882b**	Ca_16512.1|scaffold01835:10815-12377|minus|	gi|502145722|ref|XP_004506151.1|	Cicer arietinum cytochrome P450 94A1-like
**Car-miR-4882b**	Ca_03097.2|Ca_LG_3:13860196-13865158|minus|	gi|828298695|ref|XP_012569075.1|	Cicer arietinum TMV resistance protein N-like
**Car-miR-4882b**	Ca_11689.1|scaffold00264:153879-155805|plus|	gi|502173395|ref|XP_004515319.1|	Cicer arietinum cyclin-U4-1-like
**Car-miR-8301**	Ca_19889.1|scaffold07379:705-4410|minus|	gi|502098020|ref|XP_004491136.1|	Cicer arietinum chitin elicitor receptor kinase 1-like
**Car-miR-8301**	Ca_11358.1|scaffold00207:24469-26382|minus|	gi|502090790|ref|XP_004489341.1|	Cicer arietinum cytochrome P450 82A3-like
**Car-miR-26a**	Ca_13461.1|scaffold00623:101193-104140|plus|	gi|502094940|ref|XP_004490333.1|	Cicer arietinum transcription factor bHLH49-like
**Car-miR-26a**	Ca_13461.2|scaffold00623:100978-104140|plus|	gi|502094940|ref|XP_004490333.1|	Cicer arietinum transcription factor bHLH49-like
**Car-miR-26a**	Ca_13461.3|scaffold00623:100978-104140|plus|	gi|502094940|ref|XP_004490333.1|	Cicer arietinum transcription factor bHLH49-like
**Car-miR-6722**	Ca_16601.1|scaffold01885:11490-12849|plus|	gi|502152636|ref|XP_004509019.1|	Cicer arietinum protein LOL1-like
**Car-miR-6722**	Ca_16601.2|scaffold01885:11490-13384|plus|	gi|502152636|ref|XP_004509019.1|	Cicer arietinum protein LOL1-like
**Car-miR-6090**	Ca_01207.1|Ca_LG_2:2430532-2433857|minus|	gi|502093325|ref|XP_004489905.1|	Cicer arietinum uncharacterized LOC101498334
**Car-miR-6090**	Ca_13000.1|scaffold00517:83341-86886|plus|	gi|502128823|ref|XP_004500093.1|	Cicer arietinum pentatricopeptide repeat-containing protein At2g29760
**Car-miR-7241.3**	Ca_16123.1|scaffold01603:32427-33619|plus|	gi|502140300|ref|XP_004504155.1|	Cicer arietinum protein kinase PINOID-like
**Car-miR-398c**	Ca_16650.2|scaffold01910:49591-57371|plus|	gi|502138161|ref|XP_004503308.1|	Cicer arietinum probable ATP-dependent RNA helicase DHX36-like
**Car-miR-398c**	Ca_08222.2|Ca_LG_8:4140185-4187287|plus|	gi|828324334|ref|XP_012573351.1|	Glycine max uncharacterized LOC100782481
**Car-miR-2948**	Ca_16132.1|scaffold01605:2483-4495|plus|	gi|502121898|ref|XP_004497483.1|	Cicer arietinum uncharacterized LOC101495776
**Car-miR-2948**	Ca_01012.1|Ca_LG_1:14010335-14013866|plus|	gi|502080833|ref|XP_004486696.1|	Cicer arietinum acyl-protein thioesterase 2-like
**Car-miR-2948**	Ca_11462.1|scaffold00229:53216-59108|minus|	gi|593791640|ref|XP_007158859.1|	Cicer arietinum homeobox-leucine zipper protein HDG8-like
**Car-miR-718**	Ca_02640.1|Ca_LG_3:6150173-6152777|minus|	gi|502111015|ref|XP_004493934.1|	Cicer arietinum probable adenylate kinase 1
**Car-miR-718**	Ca_06261.4|Ca_LG_5:12178090-12180525|minus|	gi|828314006|ref|XP_012571709.1|	Cicer arietinum protein L-isoaspartyl methyltransferase 2 (PIMT2)
**Car-miR-3375.2**	Ca_12065.1|scaffold00327:132803-135726|minus|	gi|502155688|ref|XP_004510156.1|	Cicer arietinum tRNA (guanine-N(7)-)-methyltransferase-like
**Car-miR-3375.2**	Ca_16118.1|scaffold01600:29356-33883|plus|	gi|502155782|ref|XP_004510190.1|	Cicer arietinum protein FRIGIDA-like
**Car-miR-3375.2**	Ca_16118.2|scaffold01600:29356-33883|plus|	gi|502155782|ref|XP_004510190.1|	Cicer arietinum protein FRIGIDA-like
**Car-miR-550a**	Ca_15745.1|scaffold01411:38959-46361|plus|	gi|502103438|ref|XP_004492282.1|	Histone-lysine N-methyltransferase, H3 lysine-9 specific SUVH4-like
**Car-miR-550a**	Ca_04062.1|Ca_LG_4:2620267-2625318|plus|	gi|502125182|ref|XP_004498831.1|	Cicer arietinum uncharacterized LOC101514791
**Car-miR-3109**	Ca_01175.1|Ca_LG_2:2031552-2033318|minus|	gi|502180544|ref|XP_004516661.1|	Cicer arietinum vitellogenin-2-like
**Car-miR-3109**	Ca_07264.1|Ca_LG_6:10234396-10244322|minus|	gi|502142587|ref|XP_004505031.1|	Cicer arietinum uncharacterized LOC101498764
**Car-miR-12**	Ca_02549.1|Ca_LG_3:4391700-4392287|plus|	gi|502112549|ref|XP_004494367.1|	Cicer arietinum translocator protein homolog

**Car-miR-12**	Ca_08917.1|scaffold00216:171851-180117|plus|	gi|657382776|gb|AES75932.2|	Cicer arietinum zinc finger CCCH domain-containing protein 13-like
**Car-miR-7883b.2**	Ca_20371.1|scaffold11177:2081-2737|minus|	gi|502162737|ref|XP_004512600.1|	Cicer arietinum kunitz-type trypsin inhibitor-like 2 protein-like
**Car-miR-7883b.2**	Ca_00240.3|Ca_LG_1:2329480-2335747|minus|	gi|502077901|ref|XP_004485779.1|	Cicer arietinum callose synthase 11-like
**Car-miR-4583**	Ca_24279	-	Cicer arietinum uncharacterized LOC101505981
**Car-miR-4583**	Ca_27101	-	Cicer arietinum uncharacterized LOC101488812
**Car-miR-4583**	Ca_22650	-	Cicer arietinum uncharacterized LOC101488812
**Car-miR-7587**	Ca_05068.1|Ca_LG_4:19284310-19284724|minus|	gi|494917241|ref|WP_007643279.1|	not found
**Car-miR-6718**	Ca_09234.1|scaffold00441:86575-96434|minus|	gi|502129984|ref|XP_004500491.1|	Cicer arietinum phosphoinositide phospholipase C 6-like
**Car-miR-6718**	Ca_09788.1|scaffold01949:88935-99707|minus|	gi|502156314|ref|XP_004510412.1|	Cicer arietinum linoleate 9S-lipoxygenase-like
**Car-miR-130**	Ca_13958.1|scaffold00768:107823-112239|minus|	gi|502169129|ref|XP_004514542.1|	Cicer arietinum probable anion transporter 3, chloroplastic-like
**Car-miR-130**	Ca_15876.1|scaffold01477:374-5541|minus|	gi|502080380|ref|XP_004486545.1|	Cicer arietinum ATP-citrate synthase beta chain protein 1-like
**Car-miR-4836**	Ca_10686.1|scaffold00077:27906-37179|minus|	gi|828300211|ref|XP_012569302.1|	Cicer arietinum uncharacterized LOC101490573
**Car-novmiR17**	Ca_19193.1|scaffold05030:655-1664|minus|	gi|502148058|ref|XP_004507021.1|	Cicer arietinum uncharacterized LOC101494931
**Car-novmiR17**	Ca_10932.1|scaffold00119:191911-193867|minus|	gi|828332365|ref|XP_012574750.1|	Medicago truncatula Cysteine-rich receptor-like protein kinase
**Car-novmiR17**	Ca_12704.1|scaffold00455:94379-98439|plus|	gi|502129261|ref|XP_004500242.1|	Phosphoribosylformylglycinamidine cyclo-ligase
**Car-novmiR17**	Ca_00574.1|Ca_LG_1:7187400-7209438|minus|	gi|502085903|ref|XP_004488044.1|	Nascent polypeptide-associated complex subunit alpha, muscle-specific form-like
**Car-miR-938**	Ca_06571.1|Ca_LG_6:1939031-1940580|plus|	gi|502137539|ref|XP_004503116.1|	Cicer arietinum U-box domain-containing protein 21-like
**Car-miR-938**	Ca_00376.1|Ca_LG_1:3732739-3736448|plus|	gi|502078554|ref|XP_004485983.1|	Cicer arietinum uncharacterized LOC101502683
**Car-miR-938**	Ca_00376.2|Ca_LG_1:3732739-3737281|plus|	gi|502078554|ref|XP_004485983.1|	Cicer arietinum uncharacterized LOC101502683
**Car-miR-938**	Ca_02748.2|Ca_LG_3:7209526-7218422|plus|	gi|502109572|ref|XP_004493676.1|	Cicer arietinum ATP-dependent RNA helicase DHX8-like
**Car-miR-938**	Ca_01243.2|Ca_LG_2:2875395-2891306|plus|	gi|502093017|ref|XP_004489861.1|	Cicer arietinum AP-1 complex subunit gamma-2-like
**Car-miR-210**	Ca_18375.1|scaffold03438:17441-19979|minus|	gi|502146999|ref|XP_004506689.1|	Cicer arietinum 30S ribosomal protein S10, chloroplastic-like
**Car-miR-210**	Ca_06953.1|Ca_LG_6:6572844-6575800|minus|	gi|828318073|ref|XP_004504417.2|	Cicer arietinum probable peptide/nitrate transporter At1g27040-like
**Car-miR-887**	Ca_08698.1|Ca_LG_8:10022862-10028275|minus|	gi|502151143|ref|XP_004508299.1|	Cicer arietinum acyl-CoA-binding domain-containing protein 4-like
**Car-miR-887**	Ca_11730.1|scaffold00270:174681-178498|minus|	gi|502106452|ref|XP_004492947.1|	Cicer arietinum uncharacterized LOC101513998
**Car-miR-887**	Ca_09866.1|scaffold01264:207254-208914|minus|	-	NOT found
**Car-miR-8780.2**	Ca_17775.1|scaffold02712:282-1250|plus|	gi|502119348|ref|XP_004496588.1|	Cicer arietinum uncharacterized LOC101504477
**Car-miR-8780.2**	Ca_01107.1|Ca_LG_2:1218925-1221446|plus|	gi|828335077|ref|XP_012575338.1|	Cicer arietinum endo-1,3(4)-beta-glucanase 1-like
**Car-miR-2277**	Ca_10292.1|scaffold01499:47919-48209|plus|	gi|502126976|ref|XP_004499514.1|	Cicer arietinum MADS-box transcription factor 20-like
**Car-miR-172b.1**	Ca_06161.1|Ca_LG_5:11041566-11043755|minus|	gi|828314253|ref|XP_012571733.1|	Cicer arietinum clathrin coat assembly protein AP180-like
**Car-miR-172b.1**	Ca_00572.1|Ca_LG_1:7127153-7144385|minus|	gi|502085820|ref|XP_004488018.1|	Cicer arietinum calcium-transporting ATPase 8, plasma membrane-type-like
**Car-miR-172b.1**	Ca_00496.1|Ca_LG_1:5764862-5768994|plus|	gi|502079951|ref|XP_004486416.1|	Cicer arietinum ferric reduction oxidase 7, chloroplastic-like
**Car-miR-172b.2**	Ca_12595.2|scaffold00423:47540-50618|minus|	gi|502123985|ref|XP_004498336.1|	Transcription factor IIIA
**Car-miR-172b.2**	Ca_12595.1|scaffold00423:47540-50618|minus|	gi|502123985|ref|XP_004498336.1|	Transcription factor IIIA
**Car-miR-172b.2**	Ca_18316.2|scaffold03348:2733-3887|plus|	gi|357504971|ref|XP_003622774.1|	Cicer arietinum EPIDERMAL PATTERNING FACTOR-like protein 9-like
**Car-miR-8013**	Ca_04059.1|Ca_LG_4:2577908-2582541|minus|	gi|502178206|ref|XP_004516199.1|	Cicer arietinum GDSL esterase/lipase At5g03610-like
**Car-miR-8013**	Ca_02538.1|Ca_LG_3:4334686-4339906|minus|	gi|828301466|ref|XP_012569511.1|	Cicer arietinum potassium transporter 5-like (HAK5)
**Car-miR-8013**	Ca_10428.3|scaffold02551:21379-24615|minus|	gi|502122452|ref|XP_004497752.1|	Cicer arietinum uncharacterized LOC101513161
**Car-miR-4818d.2**	Ca_05314.1|Ca_LG_5:1477727-1488458|minus|	gi|502131051|ref|XP_004500858.1|	Cicer arietinum epidermal growth factor receptor substrate 15-like
**Car-miR-4818d.2**	Ca_05314.2|Ca_LG_5:1477727-1488881|minus|	gi|502131051|ref|XP_004500858.1|	Cicer arietinum epidermal growth factor receptor substrate 15-like

**Car-miR-4818d.2**	Ca_16954.1|scaffold02123:26839-29855|minus|	gi|502095916|ref|XP_004490569.1|	Cicer arietinum calmodulin-binding receptor-like cytoplasmic kinase 1-like
**Car-novmiR47**	Ca_20573.1|scaffold14399:575-1336|plus|	gi|502147475|ref|XP_004506797.1|	Gamma-interferon-inducible lysosomal thiol reductase
**Car-novmiR47**	Ca_09185.3|scaffold00390:141817-143164|minus|	gi|502121239|ref|XP_004497235.1|	Cicer arietinum probable prefoldin subunit 5-like
**Car-miR-9041**	Ca_09555.1|scaffold00654:107746-111487|plus|	gi|502095714|ref|XP_004490520.1|	Cicer arietinum WRKY transcription factor 55-like
**Car-miR-9041**	Ca_00185.1|Ca_LG_1:1807650-1814000|minus|	gi|502077134|ref|XP_004485562.1|	Cicer arietinum probable protein phosphatase 2C 62-like
**Car-miR-9041**	Ca_20090.1|scaffold08709:5031-5678|plus|	gi|665522180|ref|WP_031124801.1|	NOT found
**Car-miR-6562.2**	Ca_17442.1|scaffold02438:26684-29968|plus|	gi|695031200|ref|XP_009403104.1|	Cicer arietinum uncharacterized LOC101501489
**Car-miR-4818d.1**	Ca_04985.1|Ca_LG_4:18130048-18133674|minus|	gi|502115664|ref|XP_004495259.1|	Cicer arietinum uncharacterized LOC101494670
**Car-miR-4818d.1**	Ca_19805.1|scaffold06989:1352-1717|plus|	gi|828338984|ref|XP_012567630.1|	NOT found
**Car-miR-4818d.1**	Ca_10326.1|scaffold01618:34898-36512|plus|	gi|502128571|ref|XP_004500005.1|	Cicer arietinum spermidine hydroxycinnamoyl transferase-like
**Car-novmiR2**	Ca_07861.1|Ca_LG_7:7127789-7133930|plus|	gi|502152526|ref|XP_004508966.1|	Potassium transporter 5
**Car-novmiR2**	Ca_15066.1|scaffold01141:3977-5992|minus|	gi|502174748|ref|XP_004515590.1|	Sucrose transport protein SUC2
**Car-novmiR5**	Ca_02151.1|Ca_LG_2:17055901-17060849|minus|	gi|502140324|ref|XP_004504167.1|	Cicer arietinum subtilisin-like protease-like
**Car-novmiR5**	Ca_15765.1|scaffold01422:71786-75022|minus|	gi|828300859|ref|XP_012569409.1|	Cicer arietinum eukaryotic translation initiation factor 3 subunit A-like
**Car-miR-7883b.1**	Ca_09788.1|scaffold01949:88935-99707|minus|	gi|502156314|ref|XP_004510412.1|	Cicer arietinum linoleate 9S-lipoxygenase-like
**Car-miR-7883b.1**	Ca_01532.1|Ca_LG_2:6915653-6920551|minus|	gi|502098190|ref|XP_004491175.1|	Cicer arietinum probable glutamate carboxypeptidase 2-like
**Car-miR-7883b.1**	Ca_17339.1|scaffold02371:28217-42844|plus|	gi|502178612|ref|XP_004516295.1|	Probable UDP-N-acetylglucosamine--peptide N-acetylglucosaminyltransferase SPINDLY-like
**Car-miR-7883b.1**	Ca_16156.1|scaffold01613:52932-57139|minus|	gi|502104019|ref|XP_004492413.1|	Cicer arietinum peptide transporter PTR1-like
**Car-miR-rL1-25**	Ca_07781.1|Ca_LG_7:6185328-6189240|minus|	gi|828326634|ref|XP_012573707.1|	Cicer arietinum proline-rich receptor-like protein kinase PERK4-like
**Car-miR-rL1-25**	Ca_07781.2|Ca_LG_7:6185328-6189240|minus|	gi|828326637|ref|XP_012573708.1|	Cicer arietinum proline-rich receptor-like protein kinase PERK4-like
**Car-miR-3934**	Ca_07402.1|Ca_LG_7:287167-287991|minus|	gi|357478159|ref|XP_003609365.1|	Unknown
**Car-miR-3934**	Ca_18713.1|scaffold03984:5343-10194|minus|	gi|828295971|ref|XP_012568558.1|	Medicago truncatula DNA repair and recombination protein RAD54-like protein
**Car-miR-1185**	Ca_02825.1|Ca_LG_3:10030782-10032458|minus|	gi|357509133|ref|XP_003624855.1|	Phaseolus vulgaris hypothetical protein
**Car-miR-1185**	Ca_03342.1|Ca_LG_3:17157388-17158837|minus|	gi|828286823|ref|XP_012572210.1|	Cicer arietinum magnesium-dependent phosphatase 1-like
**Car-miR-1185**	Ca_03640.1|Ca_LG_3:20055908-20057644|minus|	gi|828330165|ref|XP_004511462.2|	Cicer arietinum 12-oxophytodienoate reductase 1-like
**Car-miR-1185**	Ca_08151.1|Ca_LG_8:3491889-3492871|plus|	gi|502150569|ref|XP_004508017.1|	Cicer arietinum probable protein Pop3-like
**Car-miR-1185**	Ca_13970.1|scaffold00770:97214-103043|plus|	gi|502100308|ref|XP_004491685.1|	Cicer arietinum putative E3 ubiquitin-protein ligase LIN-like
**Car-miR-763.2**	Ca_12890.1|scaffold00495:38026-41277|minus|	gi|502123635|ref|XP_004498199.1|	Cicer arietinum probable inactive serine/threonine-protein kinase bub1-like
**Car-miR-763.2**	Ca_13882.1|scaffold00745:60771-66123|plus|	gi|502140539|ref|XP_004504251.1|	Cicer arietinum exonuclease 1-like
**Car-miR-763.2**	Ca_05123.2|Ca_LG_4:20706246-20712261|minus|	gi|828303687|ref|XP_012569889.1|	Cicer arietinum histone-lysine N-methyltransferase SUVR1-like
**Car-miR-763.2**	Ca_05123.1|Ca_LG_4:20706246-20712261|minus|	gi|828303682|ref|XP_012569887.1|	Cicer arietinum histone-lysine N-methyltransferase SUVR1-like
**Car-miR-763.2**	Ca_07064.1|Ca_LG_6:7792731-7798401|plus|	gi|502141629|ref|XP_004504570.1|	Cicer arietinum uncharacterized LOC101509424
**Car-miR-763.2**	Ca_07064.2|Ca_LG_6:7792731-7798401|plus|	gi|502141629|ref|XP_004504570.1|	Cicer arietinum uncharacterized LOC101509424
**Car-miR-5349**	Ca_10942.1|scaffold00119:278253-284455|plus|	gi|502163697|ref|XP_004512920.1|	Chloroplastic group IIA intron splicing facilitator CRS1
**Car-miR-5349**	Ca_01214.1|Ca_LG_2:2484201-2488381|plus|	gi|502093357|ref|XP_004489916.1|	Cicer arietinum choline/ethanolamine kinase-like
**Car-miR-5349**	Ca_02517.1|Ca_LG_3:4158811-4168391|minus|	gi|502112413|ref|XP_004494323.1|	Cicer arietinum gelsolin-related protein of 125 kDa-like
**Car-miR-5349**	Ca_02328.1|Ca_LG_3:1720912-1724329|minus|	gi|502095118|ref|XP_004490378.1|	
**Car-novmiR44**	Ca_20440.1|scaffold12245:2291-2973|plus|	gi|357488211|ref|XP_003614393.1|	Cicer arietinum uncharacterized LOC101496645
**Car-miR-4634**	Ca_19642.1|scaffold06354:95-310|plus|	gi|657370988|gb|KEH17019.1|	Cicer arietinum uncharacterized LOC101490042

**Car-miR-4634**	Ca_18248.1|scaffold03247:154-896|minus|	gi|700198833|gb|KGN53991.1|	Cicer arietinum 5.8S ribosomal RNA gene
**Car-novmiR49**	Ca_19444.1|scaffold05689:6112-6504|plus|	gi|828334011|ref|XP_012575071.1|	Cicer arietinum uncharacterized LOC101496645
**Car-novmiR49**	Ca_21555.1|scaffold84670:2-445|minus|	gi|748749171|ref|WP_040007287.1|	Cicer arietinum uncharacterized LOC101503288
**Car-novmiR49**	Ca_20408.1|scaffold11685:2412-3806|plus|	gi|657370959|gb|KEH16993.1|	Cicer arietinum uncharacterized LOC101496645
**Car-novmiR9**	Ca_02359.1|Ca_LG_3:2711645-2718763|minus|	gi|828301846|ref|XP_012569598.1|	Cicer arietinum tankyrase-2-like
**Car-novmiR9**	Ca_07552.1|Ca_LG_7:2665739-2667031|plus|	gi|502176502|ref|XP_004515875.1|	Cicer arietinum transcription factor MYB86-like
**Car-novmiR9**	Ca_01665.1|Ca_LG_2:8340007-8341719|plus|	gi|502145172|ref|XP_004505912.1|	Cicer arietinum transcription factor MYB86-like
**Car-novmiR9**	Ca_18136.1|scaffold03081:28277-33852|plus|	gi|828327113|ref|XP_012573803.1|	Cicer arietinum uncharacterized LOC101496266
**Car-novmiR11**	Ca_00386.1|Ca_LG_1:3803322-3820036|minus|	gi|828287350|ref|XP_012568314.1|	Cicer arietinum ABC transporter C family member 2-like
**Car-novmiR11**	Ca_00386.6|Ca_LG_1:3803322-3819754|minus|	gi|828287350|ref|XP_012568314.1|	Cicer arietinum ABC transporter C family member 2-like
**Car-novmiR11**	Ca_00386.7|Ca_LG_1:3803322-3820003|minus|	gi|828287350|ref|XP_012568314.1|	Cicer arietinum ABC transporter C family member 2-like
**Car-novmiR11**	Ca_00386.2|Ca_LG_1:3803322-3819742|minus|	gi|828287350|ref|XP_012568314.1|	Cicer arietinum ABC transporter C family member 2-like
**Car-novmiR11**	Ca_00386.3|Ca_LG_1:3803322-3819782|minus|	gi|828287350|ref|XP_012568314.1|	Cicer arietinum ABC transporter C family member 2-like
**Car-novmiR14**	Ca_18701.1|scaffold03960:17068-17244|minus|	-	Cicer arietinum deneddylase UL36-like
**Car-novmiR14**	Ca_12737.1|scaffold00467:6103-9974|minus|	gi|502107124|ref|XP_004493165.1|	Cicer arietinum uncharacterized LOC101508429
**Car-miR-5988.2**	Ca_17532.1|scaffold02503:39503-43151|minus|	gi|502084332|ref|XP_004487668.1|	Cicer arietinum uncharacterized LOC101503245
**Car-miR-5988.2**	Ca_01366.1|Ca_LG_2:4791353-4795170|plus|	gi|502093776|ref|XP_004490049.1|	Probable LRR receptor-like serine/threonine-protein kinase At1g14390-like
**Car-miR-5988.2**	Ca_00589.1|Ca_LG_1:7435086-7436621|minus|	gi|502165156|ref|XP_004513444.1|	Cicer arietinum uncharacterized LOC101510445
**Car-miR-8447**	Ca_13484.1|scaffold00626:74867-75942|plus|	gi|502142721|ref|XP_004505083.1|	Cicer arietinum 18.5 kDa class I heat shock protein-like
**Car-miR-8447**	Ca_18821.1|scaffold04183:14847-16071|minus|	gi|502127498|ref|XP_004499734.1|	Cicer arietinum uncharacterized LOC101504174
**Car-miR-8447**	Ca_10381.1|scaffold03195:89538-91270|plus|	gi|502093767|ref|XP_004490046.1|	Cicer arietinum protein FEZ-like (LOC101490349)
**Car-miR-8447**	Ca_13617.1|scaffold00666:79089-90545|plus|	gi|828321153|ref|XP_004506100.2|	Cicer arietinum vacuolar protein sorting-associated protein 41 homolog
**Car-novmiR55**	Ca_15329.1|scaffold01246:31773-34317|minus|	gi|502082097|ref|XP_004487064.1|	Cicer arietinum serine carboxypeptidase-like 48-like
**Car-novmiR55**	Ca_15329.5|scaffold01246:32211-34936|minus|	gi|502082097|ref|XP_004487064.1|	Cicer arietinum serine carboxypeptidase-like 48-like
**Car-novmiR55**	Ca_16111.1|scaffold01594:46391-47246|minus|	gi|828334164|ref|XP_012575110.1|	Cicer arietinum isolate HSP6 heat shock protein gene
**Car-novmiR55**	Ca_06781.3|Ca_LG_6:4337358-4341266|plus|	gi|502090322|ref|XP_004489191.1|	Cicer arietinum U2 small nuclear ribonucleoprotein A'-like
**Car-novmiR58**	Ca_02406.1|Ca_LG_3:3214368-3219393|plus|	gi|502113470|ref|XP_004494661.1|	Cicer arietinum probable serine/threonine-protein kinase At1g54610-like
**Car-miR-1227**	Ca_05613.1|Ca_LG_5:5088971-5117129|plus|	gi|828313565|ref|XP_012571631.1|	Cicer arietinum calcium-transporting ATPase 3, endoplasmic reticulum-type-like
**Car-miR-1227**	Ca_04283.1|Ca_LG_4:6286775-6289928|minus|	gi|502122591|ref|XP_004497820.1|	Cicer arietinum eukaryotic translation initiation factor isoform 4E-2-like
**Car-miR-2690**	Ca_21677.1|scaffold134953:65-244|plus|	gi|357488201|ref|XP_003614388.1|	Cicer arietinum uncharacterized LOC101496645
**Car-miR-2690**	Ca_08665.3|Ca_LG_8:9017488-9020259|plus|	gi|502175800|ref|XP_004515743.1|	Cicer arietinum elongation factor G, chloroplastic-like
**Car-miR-2690**	Ca_20761.1|scaffold19560:573-938|minus|	gi|828334011|ref|XP_012575071.1|	not found
**Car-miR-2690**	Ca_21555.1|scaffold84670:2-445|minus|	gi|748749171|ref|WP_040007287.1|	not found
**Car-miR-615**	Ca_15649.1|scaffold01377:47761-52435|plus|	gi|657373460|gb|AET02525.2|	Cicer arietinum cysteine-rich receptor-like protein kinase 10-like
**Car-miR-615**	Ca_15649.2|scaffold01377:47761-53830|plus|	gi|657373460|gb|AET02525.2|	Cicer arietinum cysteine-rich receptor-like protein kinase 10-like
**Car-miR-615**	Ca_11173.1|scaffold00173:110043-143798|minus|	gi|297734944|emb|CBI17178.3|	Cicer arietinum ABC transporter G family member 15-like
**Car-miR-1273g**	Ca_22927.1|scaffold175432:40-423|minus|	gi|760108097|ref|WP_043790268.1|	unknown
**Car-miR-1273g**	Ca_08540.1|Ca_LG_8:7780081-7780953|minus|	gi|502152279|ref|XP_004508851.1|	Cicer arietinum uncharacterized LOC101489558
**Car-miR-1273g**	Ca_02534.1|Ca_LG_3:4282582-4293335|minus|	gi|828302165|ref|XP_012569668.1|	Medicago truncatula Cullin-like protein1
**Car-miR-482a**	Ca_00490.1|Ca_LG_1:5716569-5724019|minus|	gi|828288179|ref|XP_012569658.1|	Cicer arietinum uncharacterized LOC101503771

**Car-miR-482a**	Ca_06753.1|Ca_LG_6:4081873-4089864|plus|	gi|502138292|ref|XP_004503351.1|	Cicer arietinum E3 ubiquitin protein ligase RIE1-like
**Car-miR-482a**	Ca_09010.1|scaffold00278:22346-24175|plus|	gi|564132509|gb|AHB79187.1|	Cicer arietinum clone Ca2 CC-NBS-LRR disease resistance protein gene
**Car-miR-482a**	Ca_03332.1|Ca_LG_3:17087533-17088942|plus|	gi|502077323|ref|XP_004485619.1|	Cicer arietinum uncharacterized LOC101498868
**Car-miR-H15**	Ca_06649.1|Ca_LG_6:2900479-2910085|minus|	gi|502138825|ref|XP_004503543.1|	Cicer arietinum isoamylase 1, chloroplastic-like
**Car-miR-H15**	Ca_11655.1|scaffold00263:14610-16907|minus|	gi|502116437|ref|XP_004495431.1|	Probable leucine-rich repeat receptor-like protein kinase At5g63930-like
**Car-miR-H15**	Ca_15529.1|scaffold01332:57021-60500|plus|	gi|502155506|ref|XP_004510096.1|	Cicer arietinum serine/threonine-protein kinase STN8, chloroplastic-like
**Car-miR-995**	Ca_11558.1|scaffold00252:85857-89448|plus|	gi|502174911|ref|XP_004515628.1|	Cicer arietinum B3 domain-containing transcription factor ABI3-like
**Car-miR-995**	Ca_01679.1|Ca_LG_2:8567158-8575971|minus|	gi|828320970|ref|XP_012572769.1|	Cicer arietinum two-component response regulator-like APRR7-like
**Car-miR-995**	Ca_00959.1|Ca_LG_1:13532834-13535512|plus|	gi|502081081|ref|XP_004486760.1|	Cicer arietinum ALA-interacting subunit 1-like
**Car-miR-395a**	Ca_09356.1|scaffold00514:216297-222395|plus|	gi|502154584|ref|XP_004509751.1|	Cicer arietinum probable transcription factor KAN2-like
**Car-miR-395a**	Ca_00574.1|Ca_LG_1:7187400-7209438|minus|	gi|502085903|ref|XP_004488044.1|	not found
**Car-miR-395a**	Ca_03155.1|Ca_LG_3:14949461-14950858|minus|	gi|502090020|ref|XP_004489095.1|	Cicer arietinum uncharacterized LOC101494006
**Car-miR-395a**	Ca_07128.1|Ca_LG_6:8656298-8662579|minus|	gi|502141797|ref|XP_004504643.1|	Cicer arietinum DNA polymerase eta-like
**Car-miR-395a**	Ca_03813.1|Ca_LG_3:22651251-22652226|minus|	gi|502164149|ref|XP_004513063.1|	Cicer arietinum uncharacterized LOC101493710
**Car-miR-H14**	Ca_16955.1|scaffold02127:3480-4991|plus|	gi|502129714|ref|XP_004500401.1|	Cicer arietinum NAD(P)H-quinone oxidoreductase subunit N-like
**Car-miR-H14**	Ca_16955.2|scaffold02127:3480-4991|plus|	gi|502129714|ref|XP_004500401.1|	Cicer arietinum NAD(P)H-quinone oxidoreductase subunit N-like
**Car-miR-9218**	Ca_06084.1|Ca_LG_5:9970938-9974092|plus|	gi|502136415|ref|XP_004502678.1|	Cicer arietinum uncharacterized LOC101490727
**Car-miR-9218**	Ca_16657.1|scaffold01914:29422-32870|plus|	gi|502118466|ref|XP_004496281.1|	Cicer arietinum aquaporin PIP2-1-like
**Car-miR-9218**	Ca_11620.1|scaffold00261:37121-70074|plus|	gi|502140407|ref|XP_004504206.1|	Cicer arietinum uncharacterized LOC101498189
**Car-miR-711**	Ca_03616.1|Ca_LG_3:19867926-19869075|minus|	gi|828329953|ref|XP_012574351.1|	Cicer arietinum transcription factor MYB39-like
**Car-miR-711**	Ca_01592.1|Ca_LG_2:7509447-7514050|minus|	gi|502098645|ref|XP_004491284.1|	Cicer arietinum transmembrane protein 56-like
**Car-miR-92b**	Ca_18788.1|scaffold04125:11117-13450|plus|	gi|657384583|gb|KEH27766.1|	Cicer arietinum uncharacterized LOC101515784
**Car-novmiR21**	Ca_00771.1|Ca_LG_1:10374927-10376723|minus|	gi|502122203|ref|XP_004497630.1|	Cicer arietinum D-tyrosyl-tRNA(Tyr) deacylase-like
**Car-novmiR21**	Ca_04936.1|Ca_LG_4:16799246-16803028|plus|	gi|502116613|ref|XP_004495515.1|	Cicer arietinum uncharacterized LOC101494889
**Car-miR-6500**	Ca_13618.1|scaffold00666:111942-117523|plus|	gi|502145565|ref|XP_004506082.1|	Cicer arietinum protein argonaute 5-like
**Car-miR-6500**	Ca_10631.1|scaffold00032:388502-391097|plus|	gi|502139181|ref|XP_004503673.1|	Cicer arietinum 1-aminocyclopropane-1-carboxylate synthase-like
**Car-miR-5032**	Ca_15595.1|scaffold01361:26694-32260|plus|	gi|828288286|ref|XP_012569856.1|	Medicago truncatula Beta-D-glucosidase
**Car-miR-5032**	Ca_18939.1|scaffold04446:13850-15471|minus|	gi|502174816|ref|XP_004515606.1|	Cicer arietinum uncharacterized LOC101495319
**Car-miR-5032**	Ca_06764.1|Ca_LG_6:4189591-4194105|minus|	gi|502139043|ref|XP_004503625.1|	Cicer arietinum long chain acyl-CoA synthetase 4-like
**Car-miR-5032**	Ca_07322.1|Ca_LG_6:10783600-10785674|minus|	gi|502142131|ref|XP_004504806.1|	Cicer arietinum metacaspase-1-like
**Car-miR-99b**	Ca_06566.1|Ca_LG_6:1884816-1892910|minus|	gi|502137516|ref|XP_004503108.1|	Cicer arietinum importin-5-like
**Car-miR-99b**	Ca_15548.1|scaffold01340:46830-52527|minus|	gi|502165795|ref|XP_004513692.1|	Cicer arietinum hydroxymethylglutaryl-CoA synthase-like
**Car-novmiR29**	Ca_07925.1|Ca_LG_7:8134678-8138050|plus|	gi|502091563|ref|XP_004489582.1|	Cicer arietinum lysine histidine transporter 1-like
**Car-novmiR29**	Ca_06678.1|Ca_LG_6:3356996-3363653|plus|	gi|502090417|ref|XP_004489221.1|	Cicer arietinum uncharacterized LOC101510100
**Car-novmiR29**	Ca_07239.1|Ca_LG_6:9944013-9945050|plus|	gi|502142320|ref|XP_004504899.1|	Cicer arietinum uncharacterized LOC101501201
**Car-miR-5352**	Ca_09221.1|scaffold00430:36718-41776|plus|	gi|502129124|ref|XP_004500191.1|	Cicer arietinum THO complex subunit 3-like
**Car-miR-5352**	Ca_05046.1|Ca_LG_4:18797021-18799968|minus|	gi|502115428|ref|XP_004495201.1|	Cicer arietinum RAN GTPase-activating protein 1-like
**Car-miR-5988.1**	Ca_17032.1|scaffold02163:15859-21248|plus|	gi|502161309|ref|XP_004512117.1|	Cicer arietinum kinesin-related protein 4-like
**Car-miR-5988.1**	Ca_04027.1|Ca_LG_4:2277651-2279159|plus|	gi|502125463|ref|XP_004498936.1|	Cicer arietinum transcription factor RAX3-like
**Car-miR-5988.1**	Ca_00653.1|Ca_LG_1:8615651-8617923|plus|	gi|502084384|ref|XP_004487679.1|	Cicer arietinum NAC domain-containing protein 19-like
**Car-novmiR33**	Ca_16708	-	Cicer arietinum uncharacterized LOC101506990

**Car-novmiR33**	Ca_00551	-	Cicer arietinum sugar transporter ERD6-like 16-like
**Car-miR-156d**	Ca_17060.1|scaffold02177:44378-48787|plus|	gi|502175726|ref|XP_004515730.1|	Cicer arietinum homeobox-leucine zipper protein ROC6-like (Glabara2)
**Car-miR-8465**	Ca_07487.1|Ca_LG_7:1583466-1588274|plus|	gi|502164748|ref|XP_004513244.1|	Cicer arietinum uncharacterized LOC101507475
**Car-miR-8465**	Ca_09775.3|scaffold00911:100522-104073|minus|	gi|502173062|ref|XP_004515245.1|	Cicer arietinum glutamate decarboxylase-like
**Car-miR-8465**	Ca_11683.1|scaffold00264:108145-111771|minus|	gi|502173415|ref|XP_004515324.1|	not found
**Car-novmiR40**	Ca_11155.1|scaffold00166:157020-159217|plus|	gi|297736783|emb|CBI25984.3|	Cicer arietinum uncharacterized LOC101496705
**Car-novmiR40**	Ca_17177.1|scaffold02257:29806-33340|plus|	gi|502176817|ref|XP_004515945.1|	Cicer arietinum probable peptide/nitrate transporter At5g28470-like
**Car-novmiR41**	Ca_15201.1|scaffold01201:6422-9052|plus|	gi|502158019|ref|XP_004510997.1|	Cicer arietinum probable receptor-like protein kinase At4g39110-like
**Car-novmiR41**	Ca_09965.1|scaffold01056:110848-115435|minus|	gi|502086652|ref|XP_004488283.1|	Cicer arietinum probable inositol transporter 2-like
**Car-novmiR41**	Ca_09364.1|scaffold00934:107744-109988|plus|	gi|502170918|ref|XP_004514946.1|	Cicer arietinum uncharacterized LOC101497173
**Car-miR-4563**	Ca_01348.1|Ca_LG_2:4483484-4488495|plus|	gi|502095022|ref|XP_004490355.1|	Cicer arietinum alpha-xylosidase 1-like
**Car-miR-4563**	Ca_15294.1|scaffold01235:44546-46466|plus|	gi|502107995|ref|XP_004493366.1|	Cicer arietinum transcription activator GLK2-like
**Car-miR-4563**	Ca_18464.1|scaffold03572:1301-1531|minus|	gi|500113881|ref|WP_011789886.1|	unknown
**Car-miR-4563**	Ca_09008.1|scaffold00278:10076-10252|plus|	-	unknown
**Car-miR-2151.2**	Ca_01932.1|Ca_LG_2:14287808-14289950|plus|	gi|502168689|ref|XP_004514446.1|	Pentatricopeptide repeat-containing protein At2g20710
**Car-miR-7037.2**	Ca_18902.1|scaffold04352:6559-7239|minus|	gi|502176156|ref|XP_004515810.1|	Cicer arietinum uncharacterized LOC101511969
**Car-miR-7037.2**	Ca_17043.2|scaffold02166:13498-20701|minus|	gi|502177366|ref|XP_004516067.1|	Cicer arietinum uncharacterized LOC101508857
**Car-miR-7687.2**	Ca_12194.1|scaffold00343:178142-179217|minus|	gi|502126076|ref|XP_004499170.1|	Cicer arietinum pentatricopeptide repeat-containing protein At5g09450
**Car-miR-7687.2**	Ca_06236.2|Ca_LG_5:11972801-11975391|plus|	gi|388515987|gb|AFK46055.1|	Glycine max proteasome subunit beta type-5-like
**Car-miR-6335**	Ca_11905.1|scaffold00297:107340-111136|minus|	gi|502096208|ref|XP_004490661.1|	Cicer arietinum laccase-3-like
**Car-miR-5473**	Ca_12311.1|scaffold00363:134135-142382|plus|	gi|502124799|ref|XP_004498677.1|	Cicer arietinum transcription factor TGA6-like
**Car-miR-5473**	Ca_21087.1|scaffold39708:31-573|minus|	gi|502150790|ref|XP_004508125.1|	Cicer arietinum cell wall / vacuolar inhibitor of fructosidase 1-like
**Car-miR-5473**	Ca_13466.1|scaffold00624:97043-107786|plus|	gi|502145954|ref|XP_004506254.1|	Cicer arietinum cellulose synthase-like protein H1-like
**Car-miR-4423.3**	Ca_11683.1|scaffold00264:108145-111771|minus|	gi|502173415|ref|XP_004515324.1|	Cicer arietinum elongator complex protein 4-like
**Car-miR-4423.3**	Ca_20816.1|scaffold22206:9-1103|minus|	gi|491102342|ref|WP_004963942.1|	unknown
**Car-miR-23a**	Ca_16567.1|scaffold01857:12001-15345|plus|	gi|502120179|ref|XP_004496874.1|	Cicer arietinum endo-1,4-beta-xylanase A-like
**Car-miR-23a**	Ca_14554.1|scaffold00943:92324-97577|minus|	gi|357445027|ref|XP_003592791.1|	Cicer arietinum protoheme IX farnesyltransferase, mitochondrial-like
**Car-miR-7635**	Ca_00618.1|Ca_LG_1:8197239-8198128|plus|	gi|653318837|gb|AIC80767.1|	Cicer arietinum cucumisin-like
**Car-miR-7635**	Ca_03056.1|Ca_LG_3:12973324-12974322|plus|	gi|828298158|ref|XP_012568972.1|	Cicer arietinum cucumisin-like
**Car-miR-96a**	Ca_02080.1|Ca_LG_2:15962809-15965222|plus|	gi|502143667|ref|XP_004505419.1|	Cicer arietinum pentatricopeptide repeat-containing protein At1g03100
**Car-miR-96a**	Ca_06722.1|Ca_LG_6:3759687-3765009|plus|	gi|828292836|ref|XP_012568022.1|	Cicer arietinum L-aspartate oxidase-like
**Car-miR-7330**	Ca_15329.5|scaffold01246:32211-34936|minus|	gi|502082097|ref|XP_004487064.1|	-
**Car-miR-7330**	Ca_15329.2|scaffold01246:32211-34936|minus|	gi|502082097|ref|XP_004487064.1|	Cicer arietinum serine carboxypeptidase-like 48-like
**Car-miR-7687.1**	Ca_00576.1|Ca_LG_1:7247874-7248611|plus|	gi|657397841|gb|AES65510.2|	Medicago truncatula Disease resistance-like protein GS4-1
**Car-miR-7687.1**	Ca_17251.1|scaffold02311:15971-19005|plus|	gi|502094637|ref|XP_004490265.1|	Cicer arietinum probable phosphatidylinositol 4-kinase type 2-beta At1g26270-like
**Car-miR-7687.1**	Ca_00572.1|Ca_LG_1:7127153-7144385|minus|	gi|502085820|ref|XP_004488018.1|	Cicer arietinum calcium-transporting ATPase 8, plasma membrane-type-like
**Car-miR-1484**	Ca_10864.1|scaffold00110:35768-41455|plus|	gi|502091345|ref|XP_004489521.1|	Cicer arietinum DNA-directed RNA polymerase III subunit RPC3-like
**Car-miR-8834a**	Ca_20882.1|scaffold26795:102-1189|plus|	gi|491107292|ref|WP_004966951.1|	unknown
**Car-miR-148a**	Ca_00159.1|Ca_LG_1:1599521-1602761|minus|	-	Cicer arietinum protein FAR1-RELATED SEQUENCE 11-like
**Car-miR-148a**	Ca_04858.1|Ca_LG_4:15995013-16001449|minus|	gi|502117164|ref|XP_004495725.1|	Cicer arietinum probable linoleate 9S-lipoxygenase 5-like

**Car-novmiR1**	Ca_05062.1|Ca_LG_4:18936815-18944575|plus|	gi|502114933|ref|XP_004495085.1|	Dolichyl-diphosphooligosaccharide--protein glycosyltransferase subunit 2-like
**Car-novmiR1**	Ca_26071.1|scaffold180456:6-644|minus|	gi|162660409|gb|EDQ48368.1|	unknown
**Car-miR-4512**	Ca_15740.1|scaffold01409:19437-25809|plus|	gi|502088088|ref|XP_004488728.1|	Probable bifunctional methylthioribulose-1-phosphate dehydratase/enolase-phosphatase E1-like
**Car-miR-502b**	Ca_12486.1|scaffold00402:161200-163115|plus|	gi|502083283|ref|XP_004487412.1|	Cicer arietinum aconitate hydratase 2, mitochondrial-like
**Car-miR-502b**	Ca_15948.1|scaffold01506:25098-27005|plus|	gi|502151901|ref|XP_004508669.1|	Cicer arietinum uncharacterized LOC101512349
**Car-miR-652**	Ca_09836.1|scaffold02835:195607-202993|minus|	gi|657401972|gb|KEH40924.1|	Cicer arietinum abscisic acid 8'-hydroxylase 2-like
**Car-miR-652**	Ca_19127.1|scaffold04865:2506-6529|minus|	gi|502103562|ref|XP_004492311.1|	Cicer arietinum solute carrier family 35 member F5-like
**Car-miR-652**	Ca_03909.1|Ca_LG_4:369874-376390|minus|	gi|502126278|ref|XP_004499247.1|	Cicer arietinum tRNA (adenine(58)-N(1))-methyltransferase non-catalytic subunit trm6-like
**Car-miR-2060c**	Ca_09875.1|scaffold02296:77520-83700|plus|	gi|828340144|ref|XP_004517208.2|	Cicer arietinum uncharacterized LOC101488971
**Car-miR-2060c**	Ca_14106.1|scaffold00805:13935-14490|minus|	gi|828291019|ref|XP_012574141.1|	Cicer arietinum uncharacterized LOC101513095
**Car-miR-2060c**	Ca_01093.1|Ca_LG_2:1039535-1043773|minus|	gi|502148795|ref|XP_004507272.1|	Cicer arietinum probable sodium-coupled neutral amino acid transporter 6-like
**Car-miR-2060c**	Ca_01434.1|Ca_LG_2:5713507-5722210|plus|	gi|502149087|ref|XP_004507387.1|	Cicer arietinum uncharacterized LOC101513307
**Car-miR-2060c**	Ca_02732.1|Ca_LG_3:7066164-7072932|minus|	gi|502122539|ref|XP_004497794.1|	Cicer arietinum serine/threonine-protein kinase Nek2-like
**Car-miR-2060c**	Ca_14080.1|scaffold00795:39926-62591|minus|	gi|502085639|ref|XP_004487964.1|	Cicer arietinum villin-4-like
**Car-novmiR7**	Ca_06069.1|Ca_LG_5:9855034-9858147|minus|	gi|502136338|ref|XP_004502650.1|	Cicer arietinum E3 ubiquitin-protein ligase SINAT3-like
**Car-novmiR7**	Ca_13675.1|scaffold00680:118370-121156|minus|	gi|502159864|ref|XP_004511556.1|	Cicer arietinum meiotic recombination protein SPO11-2-like
**Car-miR-3334**	Ca_02644.1|Ca_LG_3:6171289-6174150|plus|	gi|502111031|ref|XP_004493938.1|	Cicer arietinum ethylene-responsive transcription factor RAP2-7-like
**Car-miR-3334**	Ca_02683.1|Ca_LG_3:6552772-6572985|plus|	gi|502110141|ref|XP_004493809.1|	Cicer arietinum phospholipid--sterol O-acyltransferase-like
**Car-miR-727.1**	Ca_16525.1|scaffold01839:29331-33064|plus|	gi|828295279|ref|XP_012568451.1|	Cicer arietinum isolate Ca_14301 NBS-LRR protein gene
**Car-miR-727.1**	Ca_10013.1|scaffold01129:51957-59585|minus|	gi|502128720|ref|XP_004500058.1|	Cicer arietinum uncharacterized LOC101508559
**Car-miR-727.1**	Ca_09735.1|scaffold16831:70298-74060|minus|	gi|502084866|ref|XP_004487779.1|	Cicer arietinum dihydrolipoyl dehydrogenase-like
**Car-miR-727.1**	Ca_06443.1|Ca_LG_5:15503389-15508806|minus|	gi|823170749|ref|XP_012484577.1|	Cicer arietinum dihydrolipoyl dehydrogenase-like
**Car-miR-4989**	Ca_11760.1|scaffold00274:30613-39058|plus|	gi|674932856|emb|CDY00466.1|	Cicer arietinum uncharacterized LOC101500603
**Car-miR-4989**	Ca_09732.1|scaffold16831:56998-59857|minus|	gi|502084892|ref|XP_004487786.1|	Cicer arietinum GTP-binding protein SAR1A-like
**Car-miR-4989**	Ca_15122.1|scaffold01167:17683-18270|minus|	gi|502102570|ref|XP_004492118.1|	Cicer arietinum heat shock cognate 70 kDa protein 2-like
**Car-novmiR8**	Ca_01537.1|Ca_LG_2:6975139-6979189|minus|	gi|502098209|ref|XP_004491180.1|	Cicer arietinum probable inactive receptor kinase At5g10020-like
**Car-novmiR8**	Ca_03179.1|Ca_LG_3:15195489-15207617|minus|	gi|502078671|ref|XP_004486019.1|	Cicer arietinum cullin-4-like
**Car-novmiR8**	Ca_17018.1|scaffold02157:12044-13899|plus|	gi|502180402|ref|XP_004516631.1|	Cicer arietinum uncharacterized LOC101514780
**Car-novmiR8**	Ca_00182.1|Ca_LG_1:1769121-1771542|minus|	gi|502077119|ref|XP_004485556.1|	Cicer arietinum F-box/kelch-repeat protein At1g22040-like
**Car-miR-1730**	Ca_20063.1|scaffold08587:803-3648|plus|	gi|356509753|ref|XP_003523610.1|	Cicer arietinum putative receptor protein kinase ZmPK1-like
**Car-miR-763.1**	Ca_06902.2|Ca_LG_6:5940419-5952763|plus|	gi|828317678|ref|XP_012572228.1|	Cicer arietinum uncharacterized LOC101494820
**Car-miR-763.1**	Ca_06902.1|Ca_LG_6:5940169-5953020|plus|	gi|828317678|ref|XP_012572228.1|	Cicer arietinum uncharacterized LOC101494820
**Car-miR-763.1**	Ca_20360.1|scaffold11101:691-3671|minus|	gi|502093461|ref|XP_004489950.1|	Cicer arietinum uncharacterized LOC101513328
**Car-miR-138.1**	Ca_01811.1|Ca_LG_2:12375741-12383481|plus|	gi|828322897|ref|XP_012573113.1|	Cicer arietinum mannosyl-oligosaccharide 1,2-alpha-mannosidase MNS1-like
**Car-miR-138.1**	Ca_01247.1|Ca_LG_2:2952224-2954935|minus|	gi|502093180|ref|XP_004489864.1|	Cicer arietinum uncharacterized LOC101507460
**Car-novmiR13**	Ca_12038.1|scaffold00322:119304-121770|minus|	gi|502117770|ref|XP_004495937.1|	Cicer arietinum exopolygalacturonase clone GBGE184-like
**Car-miR-7907b.1**	Ca_02860.1|Ca_LG_3:10474933-10476163|plus|	gi|502106237|ref|XP_004492895.1|	Cicer arietinum putative DNA-binding protein ESCAROLA-like
**Car-miR-7907b.1**	Ca_16533.1|scaffold01841:37344-41283|plus|	gi|502083601|ref|XP_004487508.1|	Cicer arietinum vacuolar protein sorting-associated protein 4-like
**Car-miR-7907b.1**	Ca_10743.1|scaffold00079:99491-107175|minus|	gi|502145742|ref|XP_004506158.1|	Cicer arietinum vacuolar protein sorting-associated protein 4B-like

**Car-miR-7907b.1**	Ca_02860.1|Ca_LG_3:10474933-10476163|plus|	gi|502106237|ref|XP_004492895.1|	Cicer arietinum DEAD-box ATP-dependent RNA helicase 7-like
**Car-miR-7907b.1**	Ca_16533.1|scaffold01841:37344-41283|plus|	gi|502083601|ref|XP_004487508.1|	-
**Car-miR-7907b.1**	Ca_10743.1|scaffold00079:99491-107175|minus|	gi|502145742|ref|XP_004506158.1|	-
**Car-miR-7907b.1**	Ca_12368.1|scaffold00381:84291-88784|plus|	gi|502119517|ref|XP_004496646.1|	-
**Car-miR-361.2**	Ca_04324.1|Ca_LG_4:7256116-7279797|plus|	gi|502121590|ref|XP_004497380.1|	Cicer arietinum callose synthase 3-like
**Car-miR-361.2**	Ca_19915.1|scaffold07500:2522-5566|plus|	gi|502169718|ref|XP_004514671.1|	Cicer arietinum transmembrane 9 superfamily member 4-like
**Car-miR-361.2**	Ca_13062.1|scaffold00526:121122-128408|minus|	gi|502142765|ref|XP_004505099.1|	Cicer arietinum myosin-2 heavy chain-like
**Car-miR-7263**	Ca_17542.1|scaffold02512:11804-17365|minus|	gi|502086591|ref|XP_004488265.1|	Cicer arietinum polygalacturonase-like
**Car-miR-7263**	Ca_19528.2|scaffold05951:7183-10435|plus|	gi|502148289|ref|XP_004507108.1|	Cicer arietinum uncharacterized LOC101496135
**Car-miR-7263**	Ca_19528.1|scaffold05951:7137-10435|plus|	gi|502148289|ref|XP_004507108.1|	Cicer arietinum uncharacterized LOC101496135
**Car-miR-345**	Ca_06322.1|Ca_LG_5:12911432-12944689|minus|	gi|502130045|ref|XP_004500511.1|	Cicer arietinum probable LRR receptor-like serine/threonine-protein kinase MRH1-like
**Car-miR-4661**	Ca_09203.1|scaffold00415:223377-230623|plus|	gi|502171814|ref|XP_004515107.1|	Cicer arietinum S-type anion channel SLAH3-like
**Car-miR-4661**	Ca_09203.2|scaffold00415:223377-230623|plus|	gi|502171814|ref|XP_004515107.1|	Cicer arietinum S-type anion channel SLAH3-like
**Car-miR-4661**	Ca_09203.3|scaffold00415:223377-230623|plus|	gi|502171814|ref|XP_004515107.1|	Cicer arietinum S-type anion channel SLAH3-like
**Car-miR-6222**	Ca_00635.1|Ca_LG_1:8364355-8368379|plus|	gi|502084459|ref|XP_004487694.1|	Cicer arietinum nucleolar protein 58-like
**Car-miR-6222**	Ca_00320.1|Ca_LG_1:3153713-3158240|minus|	gi|657396699|gb|KEH36609.1|	Medicago truncatula Protein STRUBBELIG-RECEPTOR FAMILY
**Car-miR-9279a**	Ca_10136.1|scaffold01534:33600-48795|plus|	gi|828297869|ref|XP_012568922.1|	Cicer arietinum heat shock cognate 70 kDa protein-like
**Car-miR-9279a**	Ca_03911.1|Ca_LG_4:397826-401556|plus|	gi|502126342|ref|XP_004499271.1|	Cicer arietinum hexokinase-2, chloroplastic-like
**Car-novmiR22**	Ca_02459.1|Ca_LG_3:3650109-3653211|minus|	gi|502113323|ref|XP_004494613.1|	Cicer arietinum protein AUXIN RESPONSE 4-like
**Car-novmiR22**	Ca_17063.1|scaffold02178:38967-43050|minus|	gi|502134043|ref|XP_004501980.1|	Cicer arietinum neutral ceramidase-like
**Car-miR-192b**	Ca_18965.1|scaffold04474:6929-9552|minus|	gi|502154756|ref|XP_004509821.1|	Cicer arietinum probable polygalacturonase non-catalytic subunit JP650-like
**Car-miR-192b**	Ca_17851.1|scaffold02781:2959-8335|plus|	gi|502094018|ref|XP_004490119.1|	Cicer arietinum protein UXT homolog
**Car-novmiR27**	Ca_20688.1|scaffold17111:46-564|minus|	gi|828334013|ref|XP_012575072.1|	Cicer arietinum uncharacterized LOC101513994
**Car-novmiR31**	Ca_06023.1|Ca_LG_5:9304547-9311189|plus|	gi|502136864|ref|XP_004502869.1|	Cicer arietinum developmentally-regulated GTP-binding protein 1-like
**Car-novmiR32**	Ca_05835.1|Ca_LG_5:7454623-7460736|plus|	gi|502087379|ref|XP_004488515.1|	Cicer arietinum origin recognition complex subunit 3-like
**Car-miR-4423.2**	Ca_10229.1|scaffold01413:23695-28002|plus|	gi|502123809|ref|XP_004498268.1|	Cicer arietinum glucan endo-1,3-beta-glucosidase 3-like
**Car-miR-4423.2**	Ca_09898.1|scaffold00995:13539-17360|plus|	gi|502123247|ref|XP_004498046.1|	Cicer arietinum glycerol-3-phosphate dehydrogenase [NAD(+)] GPDHC1, cytosolic-like
**Car-miR-4423.2**	Ca_05722.1|Ca_LG_5:6268159-6271303|minus|	gi|502133361|ref|XP_004501736.1|	Cicer arietinum uncharacterized LOC101514151
**Car-miR-4423.2**	Ca_14561.1|scaffold00944:86957-89761|minus|	gi|828322582|ref|XP_012573061.1|	Cicer arietinum uncharacterized LOC101490196
**Car-miR-4423.2**	Ca_13279.1|scaffold00582:3846-8371|plus|	gi|502139570|ref|XP_004503819.1|	Cicer arietinum (+)-neomenthol dehydrogenase-like
**Car-miR-1676**	Ca_01262.1|Ca_LG_2:3165094-3175135|plus|	gi|828293284|ref|XP_012568097.1|	Cicer arietinum uncharacterized LOC101514313
**Car-miR-1676**	Ca_01321.1|Ca_LG_2:3908050-3913092|plus|	gi|502167673|ref|XP_004514213.1|	Cicer arietinum leucoanthocyanidin dioxygenase-like
**Car-miR-1676**	Ca_02364.1|Ca_LG_3:2755615-2760950|plus|	gi|502113654|ref|XP_004494717.1|	Cicer arietinum uncharacterized LOC101506946
**Car-miR-1676**	Ca_08376.1|Ca_LG_8:5659715-5664090|plus|	gi|502151404|ref|XP_004508424.1|	Cicer arietinum pyrophosphate-energized vacuolar membrane proton pump-like
**Car-miR-252b**	Ca_16279.1|scaffold01684:45664-49338|minus|	gi|502147747|ref|XP_004506899.1|	Cicer arietinum probable peptide transporter At1g52190-like
**Car-miR-998**	Ca_12846.1|scaffold00487:131054-139128|plus|	gi|502121707|ref|XP_004497417.1|	Cicer arietinum prolyl 4-hydroxylase subunit alpha-1-like
**Car-miR-998**	Ca_14140.1|scaffold00810:98158-98625|plus|	gi|502147388|ref|XP_004506764.1|	Cicer arietinum uncharacterized LOC101513303
**Car-miR-1532**	Ca_15013.1|scaffold01122:66189-69771|plus|	gi|502148387|ref|XP_004507147.1|	Cicer arietinum UDP-glycosyltransferase 76E12-like

**Car-miR-1532**	Ca_10841.1|scaffold00105:62387-70033|minus|	gi|502161785|ref|XP_004512283.1|	Cicer arietinum serine/threonine-protein kinase ppk4-like
**Car-miR-1532**	Ca_17687.1|scaffold02635:34406-38738|minus|	gi|502146607|ref|XP_004506527.1|	Cicer arietinum arginase-like
**Car-miR-1532**	Ca_15013.1|scaffold01122:66189-69771|plus|	gi|502148387|ref|XP_004507147.1|	-
**Car-miR-1532**	Ca_10841.2|scaffold00105:62387-69986|minus|	gi|502161785|ref|XP_004512283.1|	-
**Car-miR-85**	Ca_17144.1|scaffold02237:3990-5835|plus|	gi|502155984|ref|XP_004510268.1|	icer arietinum uncharacterized LOC101497805
**Car-miR-85**	Ca_06128.1|Ca_LG_5:10728962-10730289|plus|	gi|502134362|ref|XP_004502091.1|	Cicer arietinum uncharacterized LOC101492663
**Car-miR-85**	Ca_03809.1|Ca_LG_3:22567637-22586053|plus|	gi|734419865|gb|KHN40371.1|	Cicer arietinum probable serine/threonine-protein kinase vps15-like
**Car-miR-7962**	Ca_10244.1|scaffold01829:74088-79689|plus|	gi|828307336|ref|XP_012570484.1|	Cicer arietinum transcription factor bHLH60-like
**Car-miR-7962**	Ca_13939.1|scaffold00761:66423-67927|minus|	gi|502104028|ref|XP_004492416.1|	Cicer arietinum protein SUPPRESSOR OF npr1-1, CONSTITUTIVE 1-like
**Car-miR-3375.1**	Ca_12465.1|scaffold00400:25433-27870|plus|	gi|828328489|ref|XP_012574064.1|	Cicer arietinum IAA-amino acid hydrolase ILR1-like 4-like
**Car-miR-3375.1**	Ca_05015.1|Ca_LG_4:18459053-18460700|plus|	gi|828302620|ref|XP_012569723.1|	Cicer arietinum uncharacterized LOC101500639
**Car-miR-3375.1**	Ca_18358.1|scaffold03421:19114-19735|plus|	gi|502082278|ref|XP_004487118.1|	Cicer arietinum uncharacterized LOC101515271
**Car-miR-3375.1**	Ca_13767.1|scaffold00716:102077-104252|plus|	gi|502102987|ref|XP_004492168.1|	Cicer arietinum L-type lectin-domain containing receptor kinase IV.1-like
**Car-miR-146**	Ca_25630.1|scaffold179751:44-746|minus|	gi|512585522|ref|WP_016451337.1|	unknown
**Car-miR-8623b**	Ca_16118.1|scaffold01600:29356-33883|plus|	gi|502155782|ref|XP_004510190.1|	-
**Car-miR-8623b**	Ca_08618.1|Ca_LG_8:8498383-8502815|minus|	gi|502176161|ref|XP_004515811.1|	Cicer arietinum deoxycytidylate deaminase-like
**Car-miR-8623b**	Ca_02387.1|Ca_LG_3:2951982-2954595|plus|	gi|502113510|ref|XP_004494673.1|	Cicer arietinum proteasome subunit beta type-4-like
**Car-miR-8623b**	Ca_13926.1|scaffold00752:33627-39846|minus|	gi|502108242|ref|XP_004493424.1|	Cicer arietinum uncharacterized LOC101511202
**Car-novmiR38**	Ca_07917.1|Ca_LG_7:8009708-8013312|plus|	gi|502156540|ref|XP_004510525.1|	Cicer arietinum transmembrane protein 87A-like
**Car-miR-252**	Ca_20455.1|scaffold12407:2300-3475|minus|	gi|502146535|ref|XP_004506495.1|	Cicer arietinum chaperone protein DnaJ-like
**Car-miR-252**	Ca_14347.1|scaffold00872:62380-64177|minus|	gi|502152669|ref|XP_004509035.1|	Cicer arietinum pentatricopeptide repeat-containing protein At3g29230-like
**Car-miR-888**	Ca_15107.1|scaffold01158:73824-78268|minus|	gi|502127752|ref|XP_004499821.1|	G-type lectin S-receptor-like serine/threonine-protein kinase At4g27290-like
**Car-miR-8885**	Ca_05482.1|Ca_LG_5:3306800-3310530|minus|	gi|629099892|gb|KCW65657.1|	Cicer arietinum 60S acidic ribosomal protein P0-like
**Car-miR-7915**	Ca_05959.1|Ca_LG_5:8725956-8728882|minus|	gi|502136647|ref|XP_004502769.1|	Cicer arietinum caffeic acid 3-O-methyltransferase-like
**Car-miR-7915**	Ca_00630.1|Ca_LG_1:8335043-8338015|plus|	gi|502084474|ref|XP_004487697.1|	Cicer arietinum uncharacterized LOC101512675
**Car-miR-7915**	Ca_16796.3|scaffold02017:19733-23184|plus|	gi|502145546|ref|XP_004506073.1|	RING finger and CHY zinc finger domain-containing protein 1-like
**Car-miR-7915**	Ca_17117.1|scaffold02215:17873-34573|plus|	gi|828338429|ref|XP_004516223.2|	Cicer arietinum glutamic acid-rich protein-like
**Car-miR-1144a.2**	Ca_02659.1|Ca_LG_3:6322855-6326322|plus|	gi|502105032|ref|XP_004492706.1|	Cicer arietinum bifunctional protein FolD-like
**Car-miR-1144a.2**	Ca_14445.1|scaffold00906:85863-101073|plus|	gi|502123652|ref|XP_004498207.1|	Cicer arietinum helicase domino-like
**Car-miR-5939**	Ca_18472.1|scaffold03589:16464-26087|minus|	gi|502080226|ref|XP_004486499.1|	Cicer arietinum uncharacterized LOC101510295
**Car-miR-5939**	Ca_09951.2|scaffold01039:37484-42128|plus|	gi|502090149|ref|XP_004489136.1|	Cicer arietinum probable peptide/nitrate transporter At5g13400-like
**Car-miR-6550**	Ca_21269.1|scaffold52276:255-640|plus|	gi|567195017|ref|XP_006405916.1|	Eutrema salsugineum hypothetical protein
**Car-miR-6550**	Ca_09931.1|scaffold01023:241971-242753|plus|	gi|703137890|ref|XP_010106597.1|	Astragalus lehmannianus 18S ribosomal RNA gene, partial sequence
**Car-miR-140**	Ca_19332.1|scaffold05386:1660-8801|plus|	gi|502168944|ref|XP_004514500.1|	Cicer arietinum probable nitrite transporter At1g68570-like
**Car-miR-140**	Ca_00229.1|Ca_LG_1:2213493-2218203|minus|	gi|828287668|ref|XP_012568770.1|	Cicer arietinum uncharacterized LOC101498769
**Car-novmiR45**	Ca_18675.1|scaffold03919:13336-21221|minus|	gi|356550846|ref|XP_003543794.1|	Medicago truncatula Aminoacylase-1
**Car-novmiR45**	Ca_08424.1|Ca_LG_8:6136080-6142053|minus|	gi|502151294|ref|XP_004508370.1|	Cicer arietinum UPF0613 protein PB24D3.06c-like
**Car-novmiR45**	Ca_02897.1|Ca_LG_3:11007862-11008940|minus|	gi|502105763|ref|XP_004492881.1|	Cicer arietinum uncharacterized LOC101494553
**Car-miR-2846**	Ca_03983.1|Ca_LG_4:1316344-1319734|plus|	gi|502125781|ref|XP_004499061.1|	Cicer arietinum UDP-glucuronate:xylan alpha-glucuronosyltransferase 2-like
**Car-miR-2846**	Ca_03983.2|Ca_LG_4:1316344-1319734|plus|	gi|502125781|ref|XP_004499061.1|	Cicer arietinum UDP-glucuronate:xylan alpha-glucuronosyltransferase 2-like
**Car-miR-2584**	Ca_12382.1|scaffold00382:86038-97021|plus|	gi|502089723|ref|XP_004488999.1|	Cicer arietinum probable LRR receptor-like serine/threonine-protein kinase

			At1g53430-like
**Car-miR-2584**	Ca_07545.1|Ca_LG_7:2433053-2434893|minus|	gi|502155341|ref|XP_004510038.1|	Cicer arietinum BAH and coiled-coil domain-containing protein 1-like
**Car-miR-39**	Ca_19359.1|scaffold05450:5107-12272|plus|	gi|828311252|ref|XP_004500172.2|	Cicer arietinum proteasome activator complex subunit 4-like
**Car-miR-39**	Ca_09223.1|scaffold00430:80054-89928|plus|	gi|828311339|ref|XP_012571271.1|	Cicer arietinum proteasome activator complex subunit 4-like
**Car-novmiR57**	Ca_19638.1|scaffold06333:1544-5122|minus|	gi|502165037|ref|XP_004513385.1|	Cicer arietinum scarecrow-like protein 3-like
**Car-novmiR57**	Ca_01919.1|Ca_LG_2:14110400-14115878|plus|	gi|502143792|ref|XP_004505467.1|	Cicer arietinum probable alpha,alpha-trehalose-phosphate synthase [UDP-forming] 7-like
**Car-novmiR59**	Ca_12015.1|scaffold00316:165262-168282|plus|	gi|502155558|ref|XP_004510119.1|	Cicer arietinum sterol 14-demethylase-like (
**Car-miR-2838**	Ca_17286.1|scaffold02335:12767-15735|minus|	gi|388516095|gb|AFK46109.1|	Cicer arietinum U-box domain-containing protein 4-like
**Car-miR-2838**	Ca_07913.1|Ca_LG_7:7944849-7950278|minus|	gi|502156512|ref|XP_004510511.1|	Cicer arietinum uncharacterized LOC101498678

Supplementary Table S5- Comparative expression profile of predicted miRNAs and their respective target genes as available in root transcriptome data under

**miRNA Name**	**C**	**PEG**		**NaCl**			**CDS Name**	**C**	**PEG**	**NaCl**
**Car-miR-148a**	0.75751	0.36967	1	0.6005	0.22627		**Ca_00159**	1	0.2009	1.07963
**Car-novmiR8**	1	0	0	0	0		**Ca_00182**	1	-0.8328	0.9292
**Car-miR-9041**	1	0.26619	0.44004	0	0.59741		**Ca_00185**	1	0.50233	2.55232
**Car-miR-B14RC**	0	0.89543	0.37007	1	0.75362		**Ca_00198**	1	0.01055	-0.3519
**Car-miR-140**	0.58222	0.58117	0.8967	0.51923	1		**Ca_00229**	1	-1.0002	1.45611
**Car-miR-7883b.2**	0.21042	0.42568	1	0.50041	0.62854		**Ca_00240**	1	0.24891	0.7682
**Car-miR-2575**	0	1	0	0	0		**Ca_00241**	1	-0.1878	2.28939
**Car-miR-6612.2**	0	1	0.82656	0	0		**Ca_00267**	1	0.3207	0.69157
**Car-miR-8322**	1	0.26619	0.44004	0	0		**Ca_00436**	1	1.38741	2.2435
**Car-miR-9055.2**	0.22725	0.6775	1	0.4864	0.44802		**Ca_00453**	1	-1.5374	1.1222
**Car-novmiR53**	0	1	0	0	0		**Ca_00523**	1	-0.3514	0.46252
**Car-miR-172b.1**	1	0.79855	0	0	0.39827		**Ca_00572**	1	-0.0632	0.25073
**Car-miR-172b.1**	1	0.79855	0	0	0.39827		**Ca_00572**	1	-0.0632	0.25073
**Car-miR-5988.2**	1	0.53237	0.88008	0.59454	0.29871		**Ca_00589**	1	4.42783	4.54517
**Car-miR-495**	0	1	0	0	0		**Ca_00600**	1	-0.3263	2.20313
**Car-miR-5988.1**	0	0	0	1	0		**Ca_00653**	1	1.33892	2.38235
**Car-miR-4412**	0.36852	0.8011	1	0.77207	0.65523		**Ca_00893**	1	1.78622	2.24709
**Car-miR-995**	0	0	0	0	1		**Ca_00959**	1	1.80E+308	1.80E+308
**Car-miR-2948**	0	1	0.62705	0.61616	0.23217		**Ca_01012**	1	-1.2035	0.45427
**Car-miR-8780.2**	1	0.26619	0	0	0.59741		**Ca_01107**	1	-0.8984	-1.7385
**Car-miR-3109**	0	1	0.62705	0.61616	0.23217		**Ca_01175**	1	1.08021	4.16105
**Car-miR-5349**	0.11544	0.19974	0.0254	0.01373	1		**Ca_01214**	1	0.03318	1.66122
**Car-miR-138.1**	0.75751	0.90737	1	0.45037	0.90509		**Ca_01247**	1	-3.8197	-1.3464
**Car-miR-1676**	0.32929	0.60626	0.02415	0.13052	1		**Ca_01321**	1	3.05461	4.36315
**Car-miR-4563**	0	0	0	0	1		**Ca_01348**	1	1.36958	0.78705
**Car-miR-7883b.1**	0.23314	0.22652	0.37446	1	0.51037		**Ca_01532**	1	0.5033	-1.2426
**Car-novmiR8**	1	0	0	0	0		**Ca_01537**	1	0.86806	-0.0095
**Car-miR-711**	0	0	0	0	1		**Ca_01592**	1	0.66568	0.97951
**Car-novmiR9**	0.94683	1	0.01344	0.36318	0.45617		**Ca_01665**	1	-0.6578	2.14217
**Car-miR-4865**	1	0.53237	0	0	0		**Ca_01845**	1	1.21131	0.06497

**Car-miR-2661**	1	0.26723	0.18277	0.35345	0.61505		**Ca_01915**	1	1.02286	1.86468
**Car-miR-932**	0.81237	0.22301	0.37776	1	0.50431		**Ca_01916**	1	1.42388	0.10402
**Car-novmiR57**	0.35441	1	0	0.16857	0.59285		**Ca_01919**	1	2.10024	2.63646
**Car-miR-2151.2**	0	0	0	0	1		**Ca_01932**	1	-0.1731	-0.4165
**Car-novmiR5**	0.28837	0.3441	0.35589	0.39808	1		**Ca_02151**	1	0.42226	0.49637
**Car-miR-8322**	1	0.26619	0.44004	0	0		**Ca_02170**	1	2.02266	0.96048
**Car-miR-215**	0	0.89113	0	0	1		**Ca_02328**	1	-0.8318	-1.5946
**Car-miR-12**	0.18599	0.99014	0.49105	0.66346	1		**Ca_02549**	1	1.55646	0.57053
**Car-miR-15c**	0	0	0.73658	0	1		**Ca_02635**	1	1.10379	0.48022
**Car-miR-3334**	1	0.53237	0.88008	0	0		**Ca_02683**	1	0.24635	1.81386
**Car-miR-2060c**	0.56066	0.82081	0.24671	1	0.41868		**Ca_02732**	1	0.3286	1.08492
**Car-miR-7907b.1**	0.58627	0.65318	1	0.46714	0.41162		**Ca_02860**	1	2.0342	2.4348
**Car-miR-6612.2**	0	1	0.82656	0	0		**Ca_02969**	1	-0.2799	-1.131
**Car-miR-4882b**	0	0.22278	0	0	1		**Ca_03097**	1	0.58277	-0.414
**Car-miR-482a**	0	0	0	0	1		**Ca_03332**	1	-1.6595	-1.9282
**Car-novmiR36**	0	1	0	0	0		**Ca_03362**	1	0.58438	-1.3086
**Car-miR-1271**	0	0.44556	0	0	1		**Ca_03423**	1	0.73047	-0.6789
**Car-miR-1144a.1**	0.19773	1	0.52204	0.70534	0.94499		**Ca_03455**	1	-0.8755	-1.9649
**Car-miR-5258**	0	1	0	0	0		**Ca_03484**	1	-0.8933	-0.2331
**Car-miR-395a**	0	0	0	0	1		**Ca_03813**	1	1.08413	3.52843
**Car-miR-652**	0.55796	0.70548	0.12276	0.82933	1		**Ca_03909**	1	-0.3467	-0.881
**Car-miR-2846**	0.64283	0.60371	1	0.13457	0.58416		**Ca_03983**	1	-1.1325	1.13679
**Car-miR-2846**	0.64283	0.60371	1	0.13457	0.58416		**Ca_03983**	1	-1.1325	1.13679
**Car-miR-8013**	1	0.38449	0	0.06606	0.59741		**Ca_04059**	1	-1.0978	-0.6908
**Car-miR-550a**	0.22018	1	0.79932	0.6218	0.55903		**Ca_04062**	1	-2.878	-2.1224
**Car-miR-1227**	0	0	0	0	1		**Ca_04283**	1	0.18046	-1.6165
**Car-miR-361.2**	0.56066	0.22386	0.98684	1	0.50241		**Ca_04324**	1	0.72054	0.76694
**Car-miR-2151.1**	1	0.26619	0.29336	0.39636	0.99568		**Ca_04410**	1	0.1199	0.34233
**Car-miR-5033**	0	0.07561	1	0.33778	0.33941		**Ca_04643**	1	-0.1382	0.501
**Car-miR-422a**	0	0	1	0	0		**Ca_04704**	1	0.42521	-0.3324
**Car-miR-6988**	0	0.44556	0	0	1		**Ca_04735**	1	-0.3343	-0.2392
**Car-novmiR12**	0	1	0	0	0		**Ca_04753**	1	0.03744	0.18497
**Car-novmiR52**	1	0.26883	0.45139	0.8116	0.61282		**Ca_04807**	1	-0.1708	0.80752
**Car-miR-7031**	0.12523	1	0.76298	0.06873	0.16689		**Ca_04845**	1	2.63607	3.06946

**Car-miR-3375.1**	0.47826	0.31826	0.4209	0.56868	1		**Ca_05015**	1	0.91374	-1.4026
**Car-novmiR43**	0	0	1	0	0.67882		**Ca_05478**	1	0.61529	1.00283
**Car-miR-3079**	0	1	0	0	0		**Ca_05955**	1	-1.5669	1.8352
**Car-novmiR36**	0	1	0	0	0		**Ca_05994**	1	-1.0594	-2.9459
**Car-miR-6830**	0.83341	0.22547	0.3894	1	0.51328		**Ca_06039**	1	-1.3567	0.80506
**Car-novmiR7**	0.80864	0.63713	0.66896	1	0.4058		**Ca_06069**	1	-0.0338	-0.4999
**Car-miR-85**	1	0	0	0	0		**Ca_06128**	1	2.66781	0.66582
**Car-miR-345**	0.52132	1	0.25489	0.45262	0.93432		**Ca_06322**	1	0.97634	-0.3897
**Car-miR-874**	1	0.26831	0.43476	0.34008	0.34052		**Ca_06399**	1	-1.2544	-1.2197
**Car-miR-727.1**	1	0	0	0	0		**Ca_06443**	1	0.02631	1.02156
**Car-miR-938**	1	0.06655	0.22002	0	0.74676		**Ca_06571**	1	3.69136	3.00794
**Car-miR-821d**	0	1	0	0	0		**Ca_06597**	1	1.14151	0.46569
**Car-miR-7031**	0.12523	1	0.76298	0.06873	0.16689		**Ca_06634**	1	-0.4288	-1.3822
**Car-miR-96a**	0.31386	0.16709	0.09207	0.3732	1		**Ca_06722**	1	0.14128	1.60473
**Car-miR-5032**	0	0	0	0	1		**Ca_06764**	1	-2.0185	-0.3107
**Car-miR-171**	0	0.75614	1	0.67556	0		**Ca_06889**	1	1.19514	0.22286
**Car-miR-821d**	0	1	0	0	0		**Ca_07015**	1	0.36551	-0.3101
**Car-miR-495**	0	1	0	0	0		**Ca_07116**	1	-1.5048	-0.1729
**Car-miR-5032**	0	0	0	0	1		**Ca_07322**	1	0.1604	0.74193
**Car-miR-4412**	0.36852	0.8011	1	0.77207	0.65523		**Ca_07364**	1	-0.7816	0.52266
**Car-novmiR54**	0.36852	0.8011	1	0.77207	0.65523		**Ca_07364**	1	-0.7816	0.52266
**Car-miR-2672**	0	0.10082	1	0.15012	0		**Ca_07364**	1	-0.7816	0.52266
**Car-miR-7188**	1	0.36383	0.49265	0.68178	0.82793		**Ca_07466**	1	-0.0729	0.09382
**Car-miR-2584**	0.50501	0.60491	1	0.30025	0.60339		**Ca_07545**	1	0.47744	-1.0527
**Car-novmiR9**	0.94683	1	0.01344	0.36318	0.45617		**Ca_07552**	1	-1.4988	0.99298
**Car-novmiR52**	1	0.26883	0.45139	0.8116	0.61282		**Ca_07573**	1	1.85835	-0.6775
**Car-miR-7154**	0	1	0	0	0		**Ca_07738**	1	1.13738	-0.1753
**Car-miR-rL1-25**	0.11956	0.17504	0.13153	0.07109	1		**Ca_07781**	1	0.0428	1.25027
**Car-novmiR2**	0.17081	0.25461	0.12026	0.12186	1		**Ca_07861**	1	-1.2521	-1.1914
**Car-miR-4864**	0	1	0	0	0		**Ca_07900**	1	1.08922	-0.5847
**Car-miR-2838**	1	0.53237	0.88008	0	0		**Ca_07913**	1	0.07804	1.77815
**Car-novmiR29**	0	0	0	0	1		**Ca_07925**	1	1.76808	-1.294
**Car-miR-5258**	0	1	0	0	0		**Ca_08071**	1	-0.5836	0.04711
**Car-miR-1185**	0.34873	0.30633	0.70588	0.53907	1		**Ca_08151**	1	-0.427	-1.771

**Car-miR-398c**	0.28033	0.89543	0.24671	1	0.33494		**Ca_08222**	1	0.86293	0.87106
**Car-miR-2151.1**	1	0.26619	0.29336	0.39636	0.99568		**Ca_08224**	1	-0.153	0.01925
**Car-novmiR19**	0.12523	1	0.76298	0.06873	0.16689		**Ca_08256**	1	0	1.80E+308
**Car-miR-1273g**	0	0	0	0	1		**Ca_08540**	1	0.77363	-0.0487
**Car-miR-8623b**	0.56066	0.67157	0.37007	1	0.66989		**Ca_08618**	1	0.38481	0.17129
**Car-novmiR43**	0	0	1	0	0.67882		**Ca_08658**	1	0.21605	1.23482
**Car-miR-2690**	0	0	0	0	1		**Ca_08665**	1	-2.4016	-1.6266
**Car-miR-4412**	0.36852	0.8011	1	0.77207	0.65523		**Ca_08669**	1	0.09159	-0.0293
**Car-miR-4412**	0.36852	0.8011	1	0.77207	0.65523		**Ca_08669**	1	0.09159	-0.0293
**Car-miR-887**	1	0.39928	0	0	0.29871		**Ca_08698**	1	0.46631	-1.0016
**Car-miR-361.3**	0	0.27982	0.18503	1	0		**Ca_08774**	1	1.88208	-2.5678
**Car-novmiR52**	1	0.26883	0.45139	0.8116	0.61282		**Ca_08937**	1	-0.3092	-1.2914
**Car-miR-7362**	0	1	0	0	0		**Ca_08994**	1	0.19296	1.14485
**Car-miR-727.2**	0	1	0	0	0		**Ca_09099**	1	1.52605	2.1132
**Car-miR-6988**	0	0.44556	0	0	1		**Ca_09127**	1	-0.2327	-2.2559
**Car-miR-4661**	0.51504	0.44556	0.90655	0.61243	1		**Ca_09203**	1	1.93699	0.34255
**Car-miR-6718**	1	0.53937	0.4246	0.52153	0.2201		**Ca_09234**	1	0.83602	-0.4447
**Car-miR-138.2**	0.0101	0.00538	1	0.006	0.26549		**Ca_09486**	1	-1.0038	0.09974
**Car-miR-727.1**	1	0	0	0	0		**Ca_09735**	1	-0.1082	-0.2178
**Car-miR-6718**	1	0.53937	0.4246	0.52153	0.2201		**Ca_09788**	1	0.23472	2.01482
**Car-miR-7883b.1**	0.23314	0.22652	0.37446	1	0.51037		**Ca_09788**	1	0.23472	2.01482
**Car-miR-4423.2**	0.32929	0.60626	0.02415	0.13052	1		**Ca_09898**	1	1.37056	0.05441
**Car-miR-6550**	0.27898	0.33417	0	0	1		**Ca_09931**	1	-0.6058	-1.1072
**Car-miR-5939**	0.6414	1	0.64512	0.65373	0.71162		**Ca_09951**	1	1.80E+308	1.80E+308
**Car-novmiR41**	0	0	0	0	1		**Ca_09965**	1	-1.5374	-1.3462
**Car-novmiR15**	0	1	0	0	0		**Ca_10078**	1	0.83874	0.74161
**Car-miR-9279a**	1	0	0	0	0		**Ca_10136**	1	4.17498	2.73694
**Car-miR-4423.2**	0.32929	0.60626	0.02415	0.13052	1		**Ca_10229**	1	0.37832	0.99543
**Car-miR-4818d.1**	1	0.26619	0.44004	0	0.59741		**Ca_10326**	1	-0.9337	-0.7242
**Car-novmiR19**	0.12523	1	0.76298	0.06873	0.16689		**Ca_10338**	1	0.06163	-0.6815
**Car-miR-6930**	0.95035	0.25397	0.42317	1	0.57338		**Ca_10515**	1	0.75948	-0.5703
**Car-miR-6500**	0	0	0	0	1		**Ca_10631**	1	0.78946	0.7644
**Car-miR-967**	0	1	0.33063	0	0		**Ca_10841**	1	0.27703	2.05635
**Car-miR-1532**	1	0	0	0	0		**Ca_10841**	1	0.27703	2.05635

**Car-miR-1532**	1	0	0	0	0		**Ca_10841**	1	0.27703	2.05635
**Car-miR-967**	0	1	0.33063	0	0		**Ca_10841**	1	0.27703	2.05635
**Car-miR-1532**	1	0	0	0	0		**Ca_10841**	1	0.27703	2.05635
**Car-novmiR17**	1	0.93165	0	0	0.59741		**Ca_10932**	1	0.38325	1.61336
**Car-miR-5349**	0.11544	0.19974	0.0254	0.01373	1		**Ca_10942**	1	0.03475	1.34444
**Car-miR-5258**	0	1	0	0	0		**Ca_11043**	1	1.24521	0.37222
**Car-miR-615**	0	0	0	0	1		**Ca_11173**	1	2.16138	0.28538
**Car-miR-7241.2**	0.23913	0.63652	0.52613	0.85303	1		**Ca_11196**	1	-0.6852	0.04098
**Car-miR-5466**	0	1	0.82656	0	0		**Ca_11378**	1	-0.5255	0.71713
**Car-novmiR43**	0	0	1	0	0.67882		**Ca_11562**	1	0.0495	0.76477
**Car-miR-H15**	0	0	0	0	1		**Ca_11655**	1	1.52776	0.36426
**Car-miR-4882b**	0	0.22278	0	0	1		**Ca_11689**	1	1.61169	1.92097
**Car-miR-6335**	0	0	0	0	1		**Ca_11905**	1	0.99909	-1.528
**Car-miR-3375.2**	0	0.08911	0.29463	0	1		**Ca_12065**	1	0.22349	0.01766
**Car-novmiR43**	0	0	1	0	0.67882		**Ca_12123**	1	0.82898	1.92657
**Car-miR-5473**	0	0	0	1	0		**Ca_12311**	1	-1.3469	-0.8956
**Car-miR-2584**	0.50501	0.60491	1	0.30025	0.60339		**Ca_12382**	1	-0.1073	0.36178
**Car-miR-3375.1**	0.47826	0.31826	0.4209	0.56868	1		**Ca_12465**	1	0.66565	1.88363
**Car-miR-1304**	0.83605	0.22692	0.37731	1	0.51028		**Ca_12578**	1	-0.8026	-1.3608
**Car-miR-172b.2**	1	0.38449	0	0.06606	0.59741		**Ca_12595**	1	-0.2251	1.67653
**Car-miR-172b.2**	1	0.38449	0	0.06606	0.59741		**Ca_12595**	1	-0.2251	1.67653
**Car-miR-763.2**	0.11956	0.17504	0.13153	0.07109	1		**Ca_12890**	1	1.58819	0.57326
**Car-miR-K12-12**	0.3237	0.58557	1	0.57344	0.49727		**Ca_13127**	1	-0.1292	-0.3811
**Car-miR-4423.2**	0.32929	0.60626	0.02415	0.13052	1		**Ca_13279**	1	-2.5277	-2.6283
**Car-novmiR19**	0.12523	1	0.76298	0.06873	0.16689		**Ca_13376**	1	0.80409	-1.5094
**Car-miR-5473**	0	0	0	1	0		**Ca_13466**	1	0.29751	1.12628
**Car-miR-8447**	1	0.53237	0.88008	0.59454	0.29871		**Ca_13484**	1	1.22335	1.49444
**Car-miR-2151.1**	1	0.26619	0.29336	0.39636	0.99568		**Ca_13505**	1	-0.9708	0.82327
**Car-miR-6500**	0	0	0	0	1		**Ca_13618**	1	1.70552	0.94812
**Car-miR-967**	0	1	0.33063	0	0		**Ca_13914**	1	-2.2719	-1.1649
**Car-miR-8623b**	0.56066	0.67157	0.37007	1	0.66989		**Ca_13926**	1	0.55545	1.48034
**Car-miR-7962**	0.56066	0.82081	0.24671	1	0.41868		**Ca_13939**	1	0.34671	-1.9993
**Car-miR-130**	1	0.79855	0	0	0.29871		**Ca_13958**	1	1.06203	-0.445
**Car-miR-1185**	0.34873	0.30633	0.70588	0.53907	1		**Ca_13970**	1	1.9438	0.48644

**Car-miR-2661**	1	0.26723	0.18277	0.35345	0.61505		**Ca_14162**	1	-2.8263	-0.019
**Car-miR-4423.2**	0.32929	0.60626	0.02415	0.13052	1		**Ca_14561**	1	-1.80E+308	-3.0907
**Car-novmiR2**	0.17081	0.25461	0.12026	0.12186	1		**Ca_15066**	1	-0.6209	0.41021
**Car-miR-888**	0.55796	0.29704	0.49105	0	1		**Ca_15107**	1	-0.2099	1.52168
**Car-miR-4989**	0.49456	0.46582	0.06696	0	1		**Ca_15122**	1	1.4613	2.74613
**Car-novmiR55**	0.9392	1	0.82656	0	0.56109		**Ca_15329**	1	-1.656	-1.4006
**Car-miR-7330**	0.15217	0.04051	0	0	1		**Ca_15329**	1	-1.656	-1.4006
**Car-novmiR55**	0.9392	1	0.82656	0	0.56109		**Ca_15329**	1	-1.656	-1.4006
**Car-miR-7330**	0.15217	0.04051	0	0	1		**Ca_15329**	1	-1.656	-1.4006
**Car-miR-99b**	0	0	0	0	1		**Ca_15548**	1	-0.5782	-1.6597
**Car-miR-5032**	0	0	0	0	1		**Ca_15595**	1	-0.123	0.47933
**Car-miR-615**	0	0	0	0	1		**Ca_15649**	1	-0.7817	-0.8428
**Car-miR-615**	0	0	0	0	1		**Ca_15649**	1	-0.7817	-0.8428
**Car-novmiR54**	0.36852	0.8011	1	0.77207	0.65523		**Ca_15730**	1	0.82924	1.09276
**Car-novmiR37**	0	0.44556	0.73657	0	1		**Ca_15787**	1	1.41856	2.12178
**Car-miR-502b**	1	0	0	0	0		**Ca_15948**	1	0.31141	1.58789
**Car-miR-3478**	0	0.30246	1	0	0		**Ca_15962**	1	-1.80E+308	-1.2955
**Car-miR-8492**	0	1	0	0	0		**Ca_16016**	1	-0.0698	1.32614
**Car-miR-252b**	1	0	0	0	0		**Ca_16279**	1	2.30965	3.05436
**Car-miR-4882b**	0	0.22278	0	0	1		**Ca_16512**	1	3.58318	5.39126
**Car-miR-727.1**	1	0	0	0	0		**Ca_16525**	1	0.1368	-1.7339
**Car-miR-23a**	0	0	0	1	0		**Ca_16567**	1	-1.8515	-2
**Car-miR-6722**	0	0.22386	0	1	0		**Ca_16601**	1	-2.0597	-1.7948
**Car-miR-6722**	0	0.22386	0	1	0		**Ca_16601**	1	-2.0597	-1.7948
**Car-miR-7907b.2**	0	1	0	0	0		**Ca_16618**	1	1.82596	2.29361
**Car-miR-871**	1	0.34299	0.5703	0.79272	0.77128		**Ca_16626**	1	-0.5964	-1.6359
**Car-miR-871**	1	0.34299	0.5703	0.79272	0.77128		**Ca_16626**	1	-0.5964	-1.6359
**Car-miR-9218**	0	0	0	0	1		**Ca_16657**	1	0.89745	1.51576
**Car-miR-7031**	0.12523	1	0.76298	0.06873	0.16689		**Ca_16744**	1	-0.7934	-2.6628
**Car-miR-7915**	0.54216	0.60131	1	0.12893	0.47234		**Ca_16796**	1	0.44289	-0.9798
**Car-miR-6612.2**	0	1	0.82656	0	0		**Ca_16809**	1	0.45052	-1.5346
**Car-miR-1304**	0.83605	0.22692	0.37731	1	0.51028		**Ca_17044**	1	-2.026	-2.1396
**Car-miR-85**	1	0	0	0	0		**Ca_17144**	1	-0.0884	0.0113
**Car-miR-7687.1**	1	0.31942	0	0.71345	0.23896		**Ca_17251**	1	0.76231	-0.5182

**Car-miR-5988.2**	1	0.53237	0.88008	0.59454	0.29871		**Ca_17532**	1	-1.0101	0.38508
**Car-miR-7263**	0.75136	1	0	0	0		**Ca_17542**	1	4.87055	2.13588
**Car-miR-7031**	0.12523	1	0.76298	0.06873	0.16689		**Ca_17556**	1	2.12163	0.06668
**Car-miR-1532**	1	0	0	0	0		**Ca_17687**	1	-0.0146	-1.3592
**Car-miR-821d**	0	1	0	0	0		**Ca_17787**	1	1.13844	1.12006
**Car-novmiR53**	0	1	0	0	0		**Ca_17898**	1	1.3839	-1.3765
**Car-novmiR9**	0.94683	1	0.01344	0.36318	0.45617		**Ca_18136**	1	-0.4917	0.26446
**Car-novmiR34**	0.31142	0.83932	0.99352	0.50917	1		**Ca_18187**	1	0.04633	-0.934
**Car-novmiR34**	0.31142	0.83932	0.99352	0.50917	1		**Ca_18187**	1	0.04633	-0.934
**Car-miR-4634**	0.13949	0.3218	0.08184	0.11058	1		**Ca_18248**	1	-0.5666	-0.5795
**Car-miR-172b.2**	1	0.38449	0	0.06606	0.59741		**Ca_18316**	1	-3.9941	-3.264
**Car-miR-5507**	0	0.44556	0	0	1		**Ca_18410**	1	0.01661	-1.4628
**Car-miR-92b**	0	0	0	0	1		**Ca_18788**	1	0.05738	-2.392
**Car-novmiR17**	1	0.93165	0	0	0.59741		**Ca_19193**	1	-1.2851	0.57519
**Car-miR-140**	0.58222	0.58117	0.8967	0.51923	1		**Ca_19332**	1	-0.1532	-0.2008
**Car-miR-2057**	0.62771	0.23671	0.09207	0	1		**Ca_19444**	1	-0.5267	-0.6142
**Car-novmiR49**	0.07609	0.18228	0.03348	0.09047	1		**Ca_19444**	1	-0.5267	-0.6142
**Car-miR-7263**	0.75136	1	0	0	0		**Ca_19528**	1	-1.7571	-1.5141
**Car-miR-7263**	0.75136	1	0	0	0		**Ca_19528**	1	-1.7571	-1.5141
**Car-miR-8301**	0	0.44556	0.36829	0	1		**Ca_19889**	1	-0.4765	-2.085
**Car-miR-171**	0	0.75614	1	0.67556	0		**Ca_20014**	1	0.48413	0.97525
**Car-novmiR16**	0	0	1	0	0		**Ca_20109**	1	-3.7456	0.63225
**Car-novmiR49**	0.07609	0.18228	0.03348	0.09047	1		**Ca_20408**	1	-0.3381	-0.4564
**Car-novmiR44**	0.07609	0.18228	0.03348	0.09047	1		**Ca_20440**	1	-0.2902	-0.3056
**Car-novmiR47**	1	0.26618	0.35203	0	0.23896		**Ca_20573**	1	1.9533	0.54375
**Car-novmiR19**	0.12523	1	0.76298	0.06873	0.16689		**Ca_20666**	1	-0.3087	-1.7451
**Car-novmiR27**	0.37198	0.17327	0.32737	0.11058	1		**Ca_20688**	1	-0.2781	-0.0798
**Car-miR-2057**	0.62771	0.23671	0.09207	0	1		**Ca_20761**	1	-0.3489	-0.4343
**Car-miR-2690**	0	0	0	0	1		**Ca_20761**	1	-0.3489	-0.4343
**Car-miR-6550**	0.27898	0.33417	0	0	1		**Ca_21269**	1	-0.526	-1.2129
**Car-miR-2057**	0.62771	0.23671	0.09207	0	1		**Ca_21555**	1	-0.3417	-0.3522
**Car-novmiR49**	0.07609	0.18228	0.03348	0.09047	1		**Ca_21555**	1	-0.3417	-0.3522
**Car-miR-2690**	0	0	0	0	1		**Ca_21555**	1	-0.3417	-0.3522
**Car-miR-2690**	0	0	0	0	1		**Ca_21677**	1	0.05497	-0.0456

Supplementary Table S6- *Cis*-elements identified in promoter region of stress responsive miRNAs.

Promoter element	Function
ABRELATERED1	ABA responsive
ABRERATCAL	ABA responsive
ACGTATERD1	Dehydration responsive
ARR1AT	Cytokinin responsive
MYB1AT	Dehydration responsive
MYCCONSENSUSAT	Dehydration responsive
DRECRTCOREAT	Dehydration responsive
NODCON1GM	Nodulation
RAV1AAT	Root specific
ROOTMOTIFTAPOX1	Root specific
WRKY71OS	Pathogenesis
CURECORECR	metal stress responsive
OSE1ROOTNODULC	Nodulation
CI-CADIANLELHC	Circardian related
AMYBOX-1	Dehydration responsive
MYB2AT	Dehydration responsive
MYBCORE	Dehydration responsive
MYB2CONSENSUSAT	Dehydration responsive
AUXRR CORE	Auxin responsive
TGA ELEMENT	Auxin responsive

Supplementary Table S7- List of probes and oligos used in this study

**Oligos for expression analysis**	
Oligo Name	Sequence 5'-3'
CaCyclinA2_F	CGCGTCAAAGGATCCAGATG
CaCyclinA2_R	CTTCTCGACAGCTCAGCAAC
CaEF1α_F	TCCACCACTTGGTCGTTTTG
CaEF1α_R	CTTAATGACACCGACAGCAACAG
Car-miR156_AS	TGTGCTCACTCTCTTCTGTCA
Car-miR159_AS	TAGAGCTCCCTTCAATCCAAA
Car-miR160_AS	GGCATACAGGGAGCCAGGCA
Car-miR162_AS	CTGGATGCAGAGGTTTATCGA
Car-miR164_AS	TTGAATTGCTAAGAGAATCCA
Car-miR166_AS	GGGGAATGAAGCCTGGTCCGA
Car-miR167_AS	AAGTTAAGATCATGCTGGCA
Car-miR168_AS	ATCCCGCCCTTGCATCAACTGA
Car-miR169_AS	CGGCAAGTCATCCTTGGCTGC
Car-miR171_AS	CGTGATATTGGCACGGCTCAATCA
Car-miR172_AS	ATGCAGCATCAAGATTCT
Car-miR390_AS	GGCGCTATCCCTCCTGAGCTT
Car-miR396_AS	AAGTTCAAGAAAGCTGTGGAA
Car-miR397_AS	CGTTGACGCTGCACTTAATCA
Car-miR398_AS	CAGGGTCGTCCTTAGATCACA
Car-miR399_AS	CAGGGCAACTCTCCTTTGGCA
Car-miR530_AS	AAAGTGCAGGTGCAAATGCA
Car-miR598_AS	TGATCATGATCATGATCACG
Car-miR828_AS	TGGAATACTCATTTGAGCAAGA
Car-miR1122_AS	CTGAAAAGTTGATGTATCTG

SnoRD24_AS	GCCTCAGCGATCTTGGTGGTT
Car-novmiR1_AS	GCACCAAGTGGAAGTCGTCGC
Car-novmiR2_AS	GTTGCTGTAGGCACCACTGT
Car-novmiR11_AS	AGAACTTCCTATTTGGTAGCCCTA
Car-novmiR34_AS	GAAGCACCAATGGAATTTTGG
Car-miR1144a.1_AS	ATGGGTGCCCAAGAAATTCC
Car-miR5507_AS	GGACGAAAAGGTCATCTATCT
Ca_02409.2_F	GGCAGTTCTTGGTAGCAGTG
Ca_02409.2_R	AATCGCCAGCATGTCGAATC
Ca_00238.1_F	ATGTCGTCTGCTCCATGTCA
Ca_00238.1_R	CCACCTTTTGAACCTGACCG
Ca_14461.1_F	CTGCCAGAGTTGTTGCAAGT
Ca_14461.1_R	AGGAGCACGAACTGACAGAA
Ca_05062.1_F	GCGACCACACCAGAAACTTT
Ca_05062.1_R	GCCAAATGTAGCAGGTACCG
Ca_18187.1_F	TTCATCTTCCTCCCTCGCTC
Ca_18187.1_R	TCTCCAAGTGCAACCTGAGA
Ca_01308.1_F	GTTTTCACTGATGATTTTCCTGCGA
Ca_01308.1_R	ATCTGATAGCAATCCAGCAT
Ca_03455.1_F	CGAGAAATTCGACACGAACA
Ca_03455.1_R	AAGGCTGCAACTCTCTCCAA
Ca_00386.1_F	GTGGTCTGCCAGGTTTTGTT
Ca_00386.1_R	TGCTCTCAAAAGCCAAGGTT
Ca_07861.1_F	TACAATGATGCCGTTGAGGA
Ca_07861.1_R	TTGTCACACTTCCAGGCAAC
Ca_10942.1_F	TTGTCGAAGACACAGCAAGG
Ca_10942.1_R	CATGGCTACAGAACGCTTCA

miR164_SL	GTTGGCTCTGGTGCAGGGTCCGAGGTATTCGCACCAGAGCCAACTTGAAT
miR397_SL	GTTGGCTCTGGTGCAGGGTCCGAGGTATTCGCACCAGAGCCAACCGTTGA
miR398_SL	GTTGGCTCTGGTGCAGGGTCCGAGGTATTCGCACCAGAGCCAACCAGGGT
miR399_SL	GTTGGCTCTGGTGCAGGGTCCGAGGTATTCGCACCAGAGCCAACCAGGGCA
novmiR1_SL	GTTGGCTCTGGTGCAGGGTCCGAGGTATTCGCACCAGAGCCAACGCACCA
novmiR2_SL	GTTGGCTCTGGTGCAGGGTCCGAGGTATTCGCACCAGAGCCAACGTTGCT
novmiR11_SL	GTTGGCTCTGGTGCAGGGTCCGAGGTATTCGCACCAGAGCCAACAGAACTT
novmiR34_SL	GTTGGCTCTGGTGCAGGGTCCGAGGTATTCGCACCAGAGCCAACGAAGCA
miR1144a.1_SL	GTTGGCTCTGGTGCAGGGTCCGAGGTATTCGCACCAGAGCCAACATGGGT
miR5507_SL	GTTGGCTCTGGTGCAGGGTCCGAGGTATTCGCACCAGAGCCAACGGACGA
miR164_F	GCGGCGGTGGATTCTCTTAGCA
miR397_F	GCGGCGGTGATTAAGTGCAGCG
miR398_F	GCGGCGGTGTGATCTAAGGACG
miR399_F	GCGGCGGTGCCAAAGGAGAGTT
novmiR1_F	GCGGCGGGCGACGACTTCCACT
novmiR2_F	GCGGCGGACAGTGGTGCCTAC
novmiR11_F	GCGGCGGTAGGGCTACCAAATAG
novmiR34_F	GCGGCGGCCAAAATTCCATTGG
miR1144a.1_F	GCGGCGGGGAATTTCTTGGGC
miR5507_F	GCGGCGGAGATAGATGACCTTT
Universal Reverse	GTGCAGGGTCCGAGGT
**Oligos for degradome preparation**	
RNA_oligo_sequence	CGACUGGAGCACGAGGACACUGACAUGGACUGAAGGAGUAGAAA
Oligo_dT_primer	GCTGTCAACGATACGCTACGTAACGGCATGACAGTG(T)24
GeneRacer5'_primer	CGACTGGAGCACGAGGACAACTGA
GeneRacer5'nested_primer	GGACACTGACATGGACTGAAGGAGTA
GeneRacer3'_primer	GCTGTCAACGATACGCTACGTAACG

GeneRacer3'nested_primer	CGCTACGTAACGGCATGACAGTG
**Oligos for Cloning**	
Ca397_F_gty	CACCAAAAaCATGATTAAGTGCAGCGT
Ca397_R_gty	TGGAGAAACATCATTGAGTGCAG
Ca5507_F_gty	CACCCTACAGATAGATGACCTTTTC
Ca5507_R_gty	TATGATAATGACTTCTTGTTAAGTTTCTC
novmir2_gty_F	CACCTGGCTGTTGACAACTGATAAC
novmir2_gty_R	CTGTTGCTGTAGGCACCACTGTTTTTTGA

Supplementary text S1- Material and methods in detail

Expression analysis

Target gene expression analysis was done by quantitative RT-PCR (qRT-PCR). First strand cDNAs were synthesized using SuperScript® III Reverse Transcriptase (Thermo Fisher Scientific, Waltham, MA) according to manufacturer's protocol. Primers for quantitative real-time PCR(qRT-PCR) analysis were designed from unique regions of transcripts. For miRNA, stem-loop primer was designed by using a 45 nts long universal stem-loop attached with reverse complement of last 5 nts of individual miRNA sequence, which provides binding region to mature miRNA in the pool. cDNA was synthesized by using pulse RT reaction. In brief, 20µl reaction containing 500ng total RNA and 2µm of stem-loop primer was loaded in thermal cycler and incubated for 30min at 16°C, followed by pulsed RT of 60 cycles at 30°C for 30 sec, 42°C for 30 sec and 50°C for 1 Sec. Reaction was incubated at 85°C for 5 min to inactive the reverse transcriptase. This product was used as template for qRT-PCR using an universal reverse primer designed from stem-loop primer. Forward primer was specific to a particular miRNA sequence but the last six nucleotides at 3'-end of miRNA were excluded. At 5'-end, extension of seven nucleotides was added to each forward primer to increase the melting temperature. Primers used in this study are provided in Supplementary table 1. qRT-PCR experiments were performed in Vii A7 Real-Time PCR System (Thermo Fisher Scientific, MA, USA) using three technical and three biological replicates. Each qRT-PCR reaction was performed in10µl reaction volume containing diluted cDNA as template, 225nM of each forward and reverse primer, and 2X Power SYBRGreen PCR master mix (Applied Biosystems CA, USA). Thermal cycling conditions were 95°C for 2 min followed by 40 cycles of 95°C for 15sec, 60°C for 1 min. One cycle for melt curve included in the last cycle of the program. Cycling condition for melt curve was 95°C for 15sec, 60°C for 1 min and 95°C for 15sec. *ELONGATION FACTOR 1-α* (*EF-1α*)gene was used as internal control to normalize the variation in amount of cDNA template. Relative expressions of genes were calculated according to delta-deltaCt method of the system.

Degradome analysis

Validation of miRNA mediated cleavage of target mRNA was done by RNA Ligase mediated- Rapid amplification of cDNA ends (RLM-RACE) method as described by Llave *et al*. [94] In brief, a RNA oligo was ligated at decapped 5'-end of cleaved mRNA using T4 RNA ligase (NEB, USA). Ligated mRNA was reverse transcribed using SuperScript™ III RT by using oligoDT primers to create RACE ready first strand cDNA with known priming sites at the 5' and 3' ends. This cDNA was amplified with 5' and 3' adapter specific primers to obtain

a population of cleaved products strictly ligated to both 5' and 3' adapters. RNA oligo sequence, adapter sequences and used primers are provided in Supplementary table 1. PCR product was directly ligated to pGEMT-Easy vector (Promega., Madison, WI). Positive clones containing fragments of different size, ranging from 250- 1000bp were selected and sequenced. Obtained sequences were mapped to chickpea transcripts and annotated by NCBI-BLAST tool (http://blast.ncbi.nlm.nih.gov/Blast.cgi).

Validation of candidate miRNA-target relationship

As described by Sparkes et al.,[95] a single colony of recombinant agrobacterium strain was cultured in 5 ml of LB media containing antibiotics kanamycin (50 mg/ml) and rifampicin (25 mg/ml) and grown overnight (28°C at 225 rpm). A 15ml LB media suspension was then inoculated with the overnight culture and grown at 28°C to an OD600 of ∼1.0. The cells were harvested by centrifugation at 5000 rpm for 2 min and resuspended in infiltration buffer (10 mM MgCl2 and 100 µM acetosyringone). Cells were washed once with infiltration buffer and then concentration of bacterial suspension was adjusted to a final desired concentration with the same buffer and left at room temperature for at least 2 h. An OD600 of 0.8 was adjusted for transient expression studies. The bacterial suspension was taken in a syringe and infiltrated through the abaxial surface of the leaf. Before infiltration, a small incision was made at the site of infiltration using a sterile needle to enhance the efficiency of infiltration. Plant was kept overnight in dark and next day it was transferred to light. Three days after infiltration, samples were harvested in triplicate for control (non-infiltrated), empty vector (agrobacterium containing empty vector plasmid) and experimental (infiltration media containing desired construct in agarobacterium) and immediately frozen in liquid nitrogen. RNA isolation and expression studies was done as described earlier in text.
